# Proteomic Analysis of Plasma Markers in Patients Maintained on Antipsychotics: Comparison to Patients Off Antipsychotics and Normal Controls

**DOI:** 10.3389/fpsyt.2022.809071

**Published:** 2022-04-25

**Authors:** Rudolf Engelke, Sami Ouanes, Suhaila Ghuloum, Rifka Chamali, Nancy Kiwan, Hina Sarwath, Frank Schmidt, Karsten Suhre, Hassen Al-Amin

**Affiliations:** ^1^Proteomics Core, Research Department, Weill Cornell Medicine in Qatar, Doha, Qatar; ^2^Psychiatry Department, Hamad Medical Corporation, Doha, Qatar; ^3^Psychiatry Department, Weill Cornell Medicine, Doha, Qatar; ^4^Bioinformatics Core, Research Department, Weill Cornell Medicine in Qatar, Doha, Qatar

**Keywords:** proteomics, antipsychotics, schizophrenia, bipolar disorder, biomarkers, metabolic syndrome

## Abstract

**Background:**

Schizophrenia (SZ) and bipolar disorder (BD) share many features: overlap in mood and psychotic symptoms, common genetic predisposition, treatment with antipsychotics (APs), and similar metabolic comorbidities. The pathophysiology of both is still not well defined, and no biomarkers can be used clinically for diagnosis and management. This study aimed to assess the plasma proteomics profile of patients with SZ and BD maintained on APs compared to those who had been off APs for 6 months and to healthy controls (HCs).

**Methods:**

We analyzed the data using functional enrichment, random forest modeling to identify potential biomarkers, and multivariate regression for the associations with metabolic abnormalities.

**Results:**

We identified several proteins known to play roles in the differentiation of the nervous system like NTRK2, CNTN1, ROBO2, and PLXNC1, which were downregulated in AP-free SZ and BD patients but were “normalized” in those on APs. Other proteins (like NCAM1 and TNFRSF17) were “normal” in AP-free patients but downregulated in patients on APs, suggesting that these changes are related to medication's effects. We found significant enrichment of proteins involved in neuronal plasticity, mainly in SZ patients on APs. Most of the proteins associated with metabolic abnormalities were more related to APs use than having SZ or BD. The biomarkers identification showed specific and sensitive results for schizophrenia, where two proteins (PRL and MRC2) produced adequate results.

**Conclusions:**

Our results confirmed the utility of blood samples to identify protein signatures and mechanisms involved in the pathophysiology and treatment of SZ and BD.

## Introduction

Schizophrenia (SZ) and bipolar disorder (BD) are the most common chronic psychiatric disorders. Research suggests a possible continuum between SZ and BD, as they share many etiological, phenotypic, and genotypic features (1). However, SZ and BD are still identified using diagnostic criteria based on clinical symptoms (Diagnostic and Statistical Manual of Mental Disorders, DSM) (2) independent of potential etiological or pathophysiological mechanisms. The phenomenological approach resulted in a clear symptom overlap between these conditions and a significant within-group heterogeneity (3, 4). This variability is primarily due to the absence of reliable biomarkers in clinical practice (5, 6). In addition, biomarkers can add to the current knowledge about the mechanism of action of antipsychotics (APs) (7).

The search for reliable biomarkers to guide the diagnosis, prognosis, and management of SZ and BD, has become increasingly important (8). Biomarker panels could completely revolutionize how these psychiatric disorders are diagnosed and managed. Proteomic-based panels might offer certain advantages over genomics. Indeed, while the genome is constant, protein levels change depending on the physiological state, thereby providing more accurate information about the dynamic physiological changes associated with the disease. Also, genes are not always expressed, and the expressed ones can produce different proteins with different physiological actions through post-transcriptional, translational, and post-translational regulatory mechanisms. Proteomics might better reflect the functional status than genomics (9).

Previous blood/plasma/serum proteomic studies showed that 202 proteins were significantly altered in SZ and 99 in BD (6). Most of these proteins are involved in glucose homeostasis, lipid metabolism, inflammatory and immune processes, or the role of growth factors (6, 10, 11). While some proteins are likely associated with the disease pathophysiology, others might be related to the non-therapeutic effects of medications. Other proteins might also predict the response to individual pharmacological interventions (6, 12). Thus, proteomic studies might be a step toward personalized psychiatry treatment (13). A biomarker-based stratification and management of SZ and BD can improve the effectiveness of the therapeutic interventions and thus greatly enhance the prognosis of these conditions (14).

However, one of the main challenges in interpreting previous studies' findings is to disentangle the effects of the psychiatric condition from those of the medications, as the vast majority of studies lacked a comparison between the drug-free and treated patients with SZ or BD (6, 15). Moreover, pharmacological agents (including mood stabilizers and APs) might reverse specific abnormalities found in untreated patients, thus masking their presence in studies involving only the patients on medications (16, 17). Hence, examining the differences between medicated patients, medication-free patients, and healthy controls (HCs) might resolve some of these issues.

In the present study, we aimed to investigate whether plasma proteins differ in SZ and BD compared to HCs and whether there were any differences between patients (SZ and BD) on APs and those who were AP-free. We also examined the associations between plasma protein levels and the components of metabolic syndrome in HCs, patients with SZ, and BD patients, both AP-free and treated with APs.

## Materials and Methods

This cross-sectional study is part of a project to assess the biopsychosocial profiles of patients maintained on APs in Qatar. The institutional review boards approved the study at Hamad Medical Corporation (HMC) and Weill Cornell Medicine in Qatar. All participants signed a written informed consent after explaining all the study procedures. This part focused on the proteomic analysis of patients with SZ or BD and maintained on APs for at least 6 months (*N* = 66). We compared the results to normal controls (no psychiatric illness and no psychotropics) (*N* = 102) and patients with similar diagnoses but were not taking APs for at least 6 months (*N* = 29). Please refer to further details on the population of Qatar, the recruitment process, the protocols, and the design for the various objectives from other published papers from this project (18, 19).

### Participants

Patients were recruited from the Mental Health Hospital at Doha, Qatar, the only Qatar psychiatric facility. The inclusion criteria for the patients on APs were age 18–65 years, no active medical problems, maintenance on APs for at least 6 months, and being able to sign informed consent. Those off APs had the same inclusion except that they were off APs for at least 6 months. The control group was recruited from the primary care clinics and the visitors of the Mental Health Hospital. The inclusion criteria included the same age group but with no mental illness or intake of APs. The study was done between 2014 and 2016. This study analyzed the data from the subjects with SZ and BD (on or off APs) only and compared them to the control subjects. It is worth noting that the patients chosen for this proteomic study were only taking APs, and the ones off APs were also not taking any other psychotropics. Further, all patients were competent to sign informed consent as per their independent psychiatrist, and patients off antipsychotics were stable enough to collaborate with the protocols and procedures of the study.

### Clinical Data Collection

All the subjects were screened using the Mini International diagnostic interview (20) to determine the presence or absence of the psychiatric diagnosis. After signing the informed consent, all subjects answered validated questionnaires on sociodemographic factors (age, gender, etc.), history of chronic medical problems and medication intake, and psychiatric history (duration of illness, type, and number of APs, etc.). The intake of APs and the medical and psychiatric information specifics were confirmed from the medical records. After fasting for 10–12 h, all subjects underwent blood testing within 1–2 days from recruitment. All the blood tests (CBC, lipid profile, HbA1c, fasting glucose, liver and kidney function tests) were done according to the same standardized procedures of Rumailah Hospital laboratories, Doha, Qatar. We also collected the vital signs (blood pressure and heart rate using General Electric Critikon Dinamap Pro 400 V2) and anthropometric measurements (height, weight, and waist and hip circumferences using Seca instruments). The waist circumference was measured at the level midway between the lowest rib and the iliac crest. Four raters were trained on administering the procedures and were randomly assigned to work with the control and patient's groups.

To evaluate the metabolic syndrome phenotype in this cohort, we collected information on the diagnostic criteria of metabolic syndrome as defined by the National Cholesterol Education Program's Adult Treatment Panel III report (ATP III) (21). Besides the proposed risk factors, waist circumference, elevated triglycerides, reduced HDL cholesterol, elevated blood pressure, and elevated fasting glucose, we also looked at the Body Mass Index (BMI) and LDL-cholesterol levels. We have combined BD and SZ patients into a single mental disorder (MD) group for simplicity reasons. Accordingly, BD+AP and SZ+AP were grouped as MD+AP.

### SOMAscan Assay

The SOMAscan assay was used to quantify 1301 proteins in 197 plasma samples. It was performed on an in-house Tecan Freedom EVO liquid handling system (Tecan Group, Maennedorf, Switzerland) utilizing the SOMAscan HTS Assay 1.3K plasma kit (SomaLogic, Boulder, CO) according to the manufacturer's instructions and as described previously (22, 23). The assay was performed in 96-well plates containing 85 plasma samples, three quality controls, and five calibrator plasma samples. Briefly, EDTA plasma samples were diluted into bins of 40, 1, and 0.05% and incubated with streptavidin-coated beads immobilized with dilution-specific SOMAmers via a photocleavable linker and biotin. After washing, bound proteins were first biotinylated and then released from beads by photocleaving the SOMAmer-bead linker. The released SOMAmer-protein complex was treated with a polyanionic competitor to disrupt unspecific interactions and recaptured on the second set of streptavidin-coated beads. Thorough washing was performed before 5′ Cy3 fluorophore-labeled SOMAmers were released under denaturing conditions, hybridized on microarray chips with SOMAmer-complementary sequences, and scanned using the SureScan G2565 Microarray Scanner (Agilent, Santa Clara, CA).

### SOMAscan Data Processing

Relative Fluorescence Units (RFUs) were obtained from microarray intensity images using the Agilent Feature Extraction Software (Agilent, Santa Clara, CA). Raw RFUs were normalized and calibrated using the software pipeline provided by SomaLogic. This calibration included (a) microarray hybridization normalization based on spiked-in hybridization controls, (b) plate-specific intensity normalization, (c) median signal normalization, and (d) analyte level median calibrator scaling of single RFU intensities according to calibrator reference values.

### Statistical Analysis

Statistical analyses were performed in R version 3.5.2 (R Foundation for Statistical Computing, Vienna, Austria) using the autonomics package (24) for data import and R base package for uni- and multivariate analysis, including Principal Component Analysis (PCA). Analysis of covariance (ANCOVA) and Tukey's *post-hoc* tests were used for differential protein analysis on single protein Log2 transformed and standardized RFUs from the SOMAscan assay with age, gender, ethnicity, and the first and second principal component scores as covariates. To determine which covariate to include in the model on a single-protein level, we have performed Lasso regression using the glmnet package (25). When performing ANCOVA, we incorporated only those covariates with a beta coefficient larger than zero. The False Discovery Rate was controlled using the Benjamini-Hochberg multiple-testing adjustment of *p*-values. The confusion matrix was derived from predicted and observed values where applicable. Random forest (RF) algorithm, as implemented in the caret package (26), was applied to develop a classification model between the groups of interest based on protein levels. Ten-fold cross-validation was used to evaluate the prediction accuracy and obtain the receiver operating characteristic (ROC).

### Functional Enrichment Analysis

Disease annotation terms and biological function enrichment analyses were performed using Ingenuity Pathway Analysis (IPA) software (v. 57662101; QIAGEN Bioinformatics, Aarhus, Denmark). Enrichment analysis was performed on differential proteins (ANCOVA *P*-value < 0.05) using 1301 proteins from the SOMAscan platform as background proteome. Annotation groups related to cancer and embryology were not considered and thus removed from further analysis. Terms with a Fisher's exact *p*-value <0.005 (arbitrary cut-off to select top 15 enriched terms) and a protein count > 18 (arbitrary cut-off to choose 10 out of 15 enriched terms) were determined and used for repeated enrichment analysis where sets of differential proteins were split according to differential expression in one of the patient groups and whether they were upregulated or downregulated.

## Results

### Uncovering the Circulating Proteome Signature

To identify plasma protein alterations associated with mental disorders (MDs) and APs treatment, we used a discovery cohort of 197 individuals. Patients diagnosed with SZ or BD, either taking AP or who stopped taking AP for more than 6 months, were recruited for this study (Table 1). The cohort had a larger fraction of males (62%) and was recruited from the Arab (57%) and South Asian (40%) population. Patients diagnosed with an MD taking APs had a higher mean BMI of 29.5 kg/m^2^ than patients who discontinued medication and HCs (Table 2).

**Table 1:** Demographic and clinical characteristics by diagnosis.

	**HC**	**MD**	**MD + AP**
Participants (*n*)	102	29 (BD:11; SZ: 18)	66 (BD: 38, SZ: 28)
Male / Female (*n*)	59 / 43	18 / 11	46 / 20
Age (yrs; M ± SD)	35.0 ± 0.9	36.0 ± 1.8	35.5 ± 1.4
Age onset of psychiatric symptoms (yrs; M ± SD)		29.0 ± 1.6	20.9 ± 1.9
Age first psychiatric diagnosis (yrs; M ± SD)		30.2 ± 2.0	21.7 ± 1.2
Duration of illness (yrs; M ± SD)		7.1 ± 2.1	11.5 ± 1.2
APs (*n*)			FGA: 12, SGA: 42, both: 12

*HC, healthy control; MD, mental disorder; APs, antipsychotics; BD, bipolar disorder; SZ, schizophrenia; yrs, years; M ± SD, mean ± standard deviation; FGA, first-generation antipsychotics; SGA, second-generation antipsychotics*.

**Table 2:** Risk factors of metabolic syndrome.

		***P*-value**		
	**ANOVA**	**MD - HC**	**MD + AP - HC**	**MD + AP - MD**
BMI	0.019	0.26	0.033	0.01
Waist Circumference (cm)	0.014	0.32	0.0037	0.25
Triglycerides (mmol/l)	0.062	0.79	0.031	0.073
HDL (mmol/l)	0.17	0.065	0.82	0.11
LDL (mmol/l)	0.52	0.52	0.48	0.27
Systolic BP (mmHg)	1.4e-04	6.4e-04	6.2e-04	0.43
Diastolic BP (mmHg)	2.9e-05	4.6e-05	1.2e-03	0.11
Fasting Glucose (mmol/l)	0.36	0.54	0.29	0.18

*ANOVA and post-hoc P-values of comparisons between MD, MD+AP, and HC. ANOVA, Analysis of Variance; MD, mental disorder; HC, healthy control; HDL, high-density lipoprotein; LDL, low-density lipoprotein; BP, blood pressure*.

**Figure 1:**
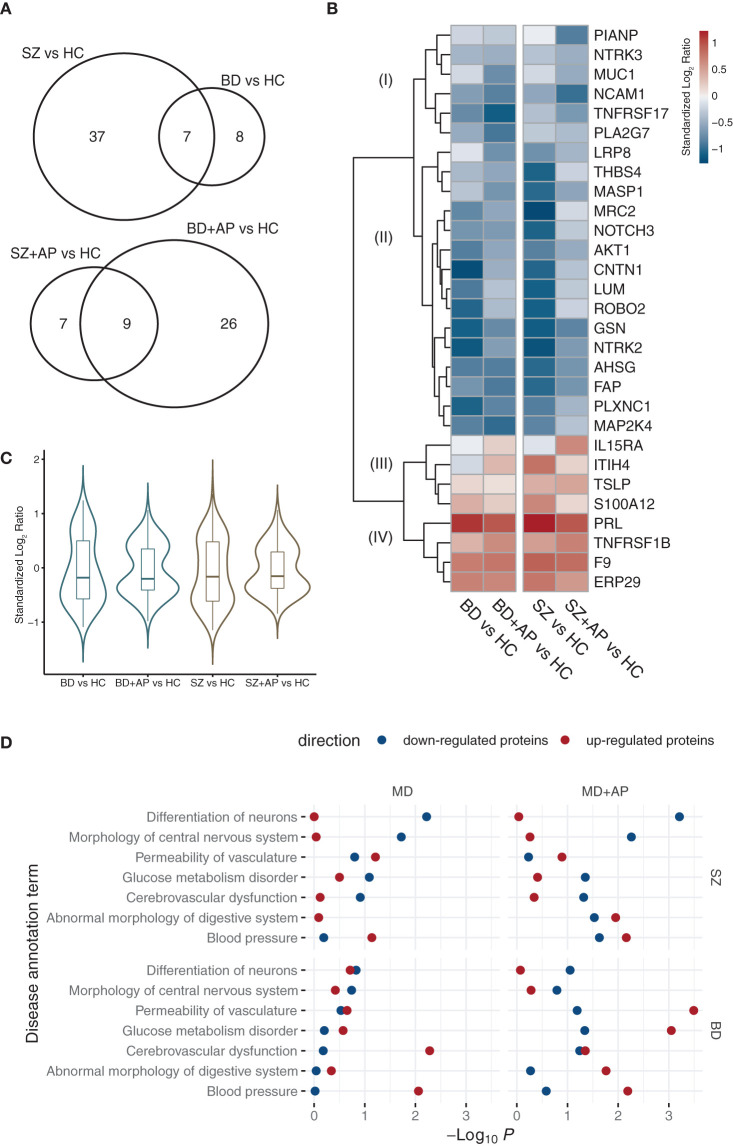
Circulating proteome profiles in SZ and BD patients (+/− AP). **(A)** Number of significantly regulated plasma proteins (*post-hoc* FDR < 0.05) in SZ and BD patients (do not receive APs for at least 6 months) and patients taking APs. **(B)** Heatmap is showing standardized log_2_ protein level ratios for most significant proteins (*post-hoc* FDR < 0.01) of SZ and BD patients (+/-AP) compared to HC. Four major clusters I-IV revealed using complete Euclidean distance clustering are labeled. **(C)** Distributions of protein level ratios of significantly changing proteins (ANCOVA *P* < 0.01) across comparisons of conditions and treatment to HCs. **(D)** Statistically over-represented IPA disease annotation terms within significantly down- or upregulated groups of proteins in SZ and BD patients (+/− AP). Statistical over-representation of terms is expressed as -log_10_ *P*-value derived from a Fisher's exact test.

The SOMAscan assay was used to assess relative levels of 1301 proteins in plasma from patients in the cohort. We performed a single-analyte ANCOVA on standardized logarithmic RFUs to identify differences between the groups of patients (+/− AP) and HC, correcting for gender, age, and ethnicity. We also controlled for the top features of the first and second principal components, accounting for plasma preparation variability described in materials and methods. We did not control for the duration of illness, as age (already included in the model) showed a strong correlation with this variable. The analysis revealed 58 ANCOVA significant proteins at a False Discovery Rate (FDR) level of 0.05 (FDR < 0.05, *P* < 2.15 × 10^−3^; Supplementary Table 1). *Post-hoc* tests indicated 44 differential proteins when comparing SZ with HC (FDR < 0.05, *P* = 1.62 × 10^−3^) and 15 ones between BD and HC (*P* = 5.68 x 10^−4^) (Figure 1A). For patients on APs, group comparisons identified 16 differential proteins between the groups SZ+AP and HC (FDR < 0.05, *P* = 5.68 × 10^−4^) and 35 ones between BD+AP and HC (FDR < 0.05, *P* = 1.33 × 10^−3^).

We plotted the standardized log_2_ RFU ratios in a heatmap to visualize the statistically significant protein changes between MD patients and HC (*post-hoc* FDR < 0.01 in one of the conditions, Figure 1B; *post-hoc* FDR < 0.05, Supplementary Figure 1). The protein profile signatures of BD and SZ (+/−AP) are qualitatively similar, showing four main clusters I to IV as revealed by the Euclidean distance metric. Cluster I contains proteins that are downregulated in patients taking AP. Cluster II contains proteins downregulated in AP-free patients (SZ, BD), which remain at basal levels in patients on AP. Interestingly, protein level distributions for a fraction of the significantly changing proteins (ANCOVA *P* < 0.01) indicated a shift of these proteins in patients on AP toward levels comparable to HC (Figure 1C).

We have assembled 223 previously published blood-based biomarkers associated with BD and SZ, from which 124 were quantified in this study (Supplementary Table 2). At FDR < 0.05 cut-off, we could replicate PRL, NTRK2, and TIMP1 in BD, and PRL, ITIH4, AHSG, TNFRSF1B, C4A, PPY, ICAM2 in SZ patients. The three most reported proteins were quantified in our results (when compared to HC) as following: APOA1 (BD: *P* = 1.07 × 10^−2^, SZ: *P* = 3.15 × 10^−2^), BDNF (BD: *P* = 9.22 × 10^−3^, SZ: *P* = 0.42), A2M (BD: *P* = 6.06 × 10^−2^, SZ: *P* = 7.69 × 10^−2^).

### Functional Enrichment Analysis

We performed enrichment analysis using the Ingenuity Pathway Studio and the incorporated Knowledge Base annotation to understand the underlying functional features and disease-related consequences shared by differential proteins. We have initially performed a broad screen by selecting differential proteins at *post-hoc P* < 0.01 (*n* = 213) for enrichment analysis and filtered for statistically enriched disease and function annotation terms. These terms were used to perform a refined enrichment analysis where the upregulated and downregulated proteins (*post-hoc* FDR < 0.05) and proteins from BD and SZ patients were tested separately. Among the top 7 groups detected, we found disease annotation groups related to a neurological disorder phenotype, such as differentiation of neurons and morphology of central nervous systems (Figure 1D). The most robust enrichment of proteins from these groups was detected in the downregulated fraction of proteins in SZ patients. Disease traits of vascular dysfunction (cerebrovascular dysfunction, the permeability of vasculature, and blood pressure) were found among proteins where levels changed in both directions among patients treated with AP. It is further prominent that proteins associated with cerebrovascular dysfunction and blood pressure were upregulated in BD and BD+AP patients.

### Biomarker Identification

**Figure 2:**
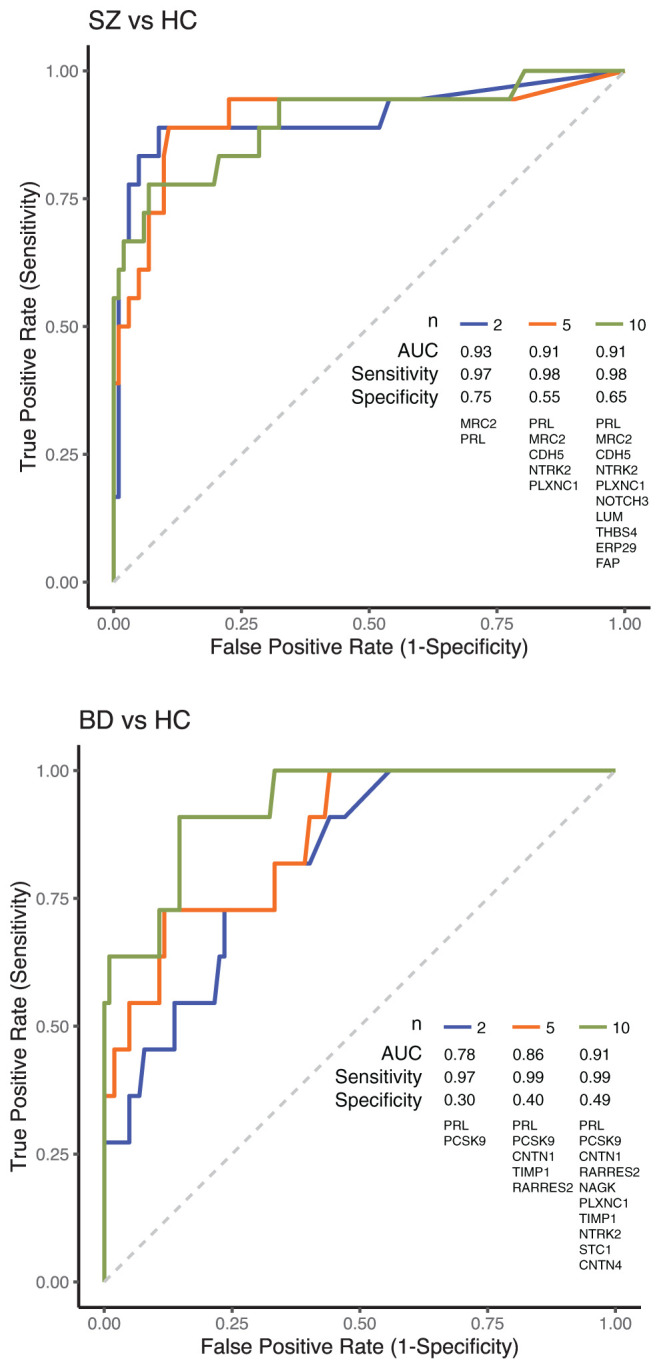
ROC curves showing the diagnostic performance in discriminating SZ and BD patients from HC. Classification between MD patients and HC was performed using 2, 5, or 10 proteins. Proteins for classification were selected from a list of the most significantly regulated proteins identified in this study, followed by training a random forest prediction model to choose the most robust protein classifiers. ROC curves and classification model performance metrics using selected protein classifiers, as shown in the plot, were established using 10-fold cross-validation.

Having revealed proteome changes in BD and SZ patient's plasma, we assessed if significantly differential proteins would be useful in clinical diagnosis. We trained a random forest model for discrimination between MD groups and HC with 10-fold cross-validation. Three models were trained to identify 2, 5, and 10 best performing protein classifiers. As shown previously, changes in protein abundance were more prominent in SZ patients. Accordingly, the diagnostic performance was better in discriminating SZ than BD patients from HC (Figure 2, Supplementary Table 3). Prediction using 2 proteins resulted in performance with AUC of 0.93 (sensitivity 97%, specificity 75%, classifier: MRC2, PRL) in SZ patients and AUC of 0.78 (sensitivity 97%, specificity 30%, classifier: PRL, PCSK9) in BD ones. For BD patients, predictive performance was improved using ten proteins for classification, leading to an AUC of 0.91 (sensitivity 91%, specificity 49%).

### Metabolic Syndrome Risk Factors Associations

**Figure 3:**
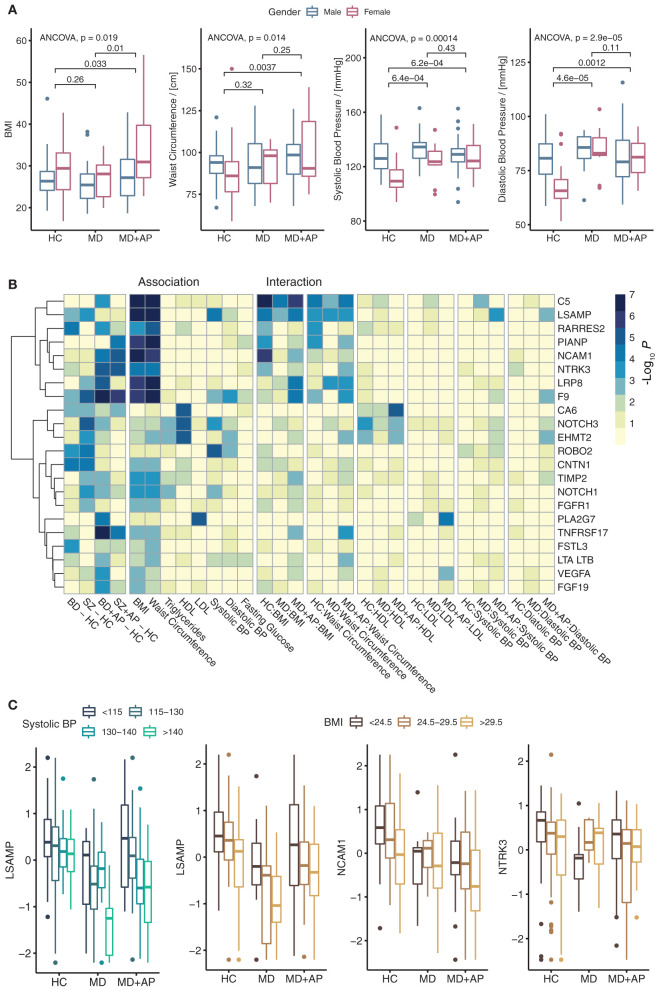
Clinical variables and their associations with differentially abundant proteins. **(A)** Boxplot indicating the distribution of BMI, waist circumference, systolic, and diastolic blood pressure across conditions. Indicated are ANCOVA *P*-values and *post-hoc P*-values for group comparisons correcting for gender and age. **(B)** Heatmap shows the significance of association for differentially abundant proteins with clinical variables. The plot shows the significance of differential abundances comparing BD and SZ to HC, the significance of these proteins for the association with a clinical variable, and the significance of interaction with the MD group (BD and SZ combined). **(C)** Boxplots for proteins were found to have a significant interaction term with a clinical variable.

Among the clinical variables of interest, BMI, waist circumference, and triglycerides were significantly higher in patients taking APs (Table 2, Figure 3A). Similar results were obtained when the analysis was performed separately for BD or SZ patients (Supplementary Table 4, Supplementary Figure 2).

To investigate plasma patterns associated with the metabolic syndrome risk factors, we performed two types of multivariate linear model analysis. First, we performed a linear regression analysis of proteins with the clinical descriptors of metabolic syndrome to characterize protein associations with metabolic syndrome risk factors. Second, to investigate how protein associations with clinical variables change within groups, we fitted an interaction model combining metabolic syndrome variable and patient group (Supplementary Table 5). All models were controlled for age and gender. The results of both analyses were filtered for significant values (main effect ANCOVA FDR < 0.05, significant association or interaction FDR < 0.05) followed by plotting of log_10_ *P*-values (Figure 3B). The results showed that many proteins are associated with either BMI or waist circumference. For example, NCAM1, NTRK3, LRP8, and TNFRSF17, changed significantly in BD+AP and SZ+AP patients compared to HC and showed significant association with BMI and waist measurement. NCAM1 and NTRK3 association decreased in patients on APs (Figure 3C), while LRP8 and TNFRSF17 association increased in patients on APs. All these proteins belong to cluster I of proteins, downregulated upon AP treatment (Figure 1B).

Further, CA6, NOTCH3, and EHMT2 were associated with HDL cholesterol, while PLA2G7 was associated with LDL-cholesterol levels. All these proteins showed an increase in the association among patients on APs. The strongest correlation with systolic blood pressure was found for LSAMP, NOTCH3, and ROBO2. Besides, LSAMP interaction with systolic and diastolic blood pressure increased in patients treated with APs (Figure 3C).

## Discussion

In this study, we have quantified 1301 proteins in plasma of unmedicated and medicated BD and SZ patients compared to HC. The majority of the significantly regulated circulating proteins in BD and SZ patients have not been reported previously. Overall, the number of regulated proteins and the degree of change were higher in SZ patients than in BD patients. In contrast, BD patients treated with APs showed a more pronounced response in protein regulation than SZ patients.

We have detected changes that are functionally associated with nervous system morphology, glucose metabolism, and vasculature permeability. These changes demonstrate the patient's physiological status concerning their disease phenotype and medication status, providing another example that blood proteins can reflect the physiological state of an individual (27). Our findings add to the previously reported proteins involved in immune response and inflammation, complement activation, oxidative stress response, and hormone signaling (10, 28, 29). For example, we have identified NTRK2, CNTN1, ROBO2, and PLXNC1 to be downregulated in BD and SZ. Further, genome-wide association studies (GWAS) reported that genes encoding these proteins had been associated with SZ and BD (30–33). These proteins were annotated to be involved in the morphology and differentiation of the nervous system. Interestingly, our data showed that these proteins were downregulated in unmedicated patients but were mostly “normalized” in patients on APs. In SZ patients, the recovery of proteins to normal levels included more proteins such as MRC2, CDH5, LUM, THBS4, MASP1, ITIH4, LSAMP, and S100A12. The fact that these biomarker's levels tended to normalize on APs might reflect that these are possible “state” markers associated with specific pathophysiological mechanisms that may be reverted, at least partially, by pharmacological treatment. Similarly, in patients with BD, circulating BDNF levels were decreased in manic and depressive episodes and to recover after treatment of the mood episode, thus suggesting its role as a “state” marker during relapses (34).

We identified a group of downregulated proteins in AP-free patients with SZ and BD but were mostly “normalized” in patients with these disorders on APs. These proteins were primarily involved in neurodevelopment, axon guidance, neuron proliferation, and survival, and included PLXNC1, NTRK2, ROBO2, and CNTN1. Many of the genes coding for these proteins have already been associated with SZ or BD in the genomic studies (35–37). Similarly, previous studies found that proteins involved in neuron survival and maturation were associated with SZ and BD. BDNF and GMFB serum levels were consistently decreased in patients with SZ (10, 38). AP use was reported to modestly increase BDNF levels in patients with SZ (10).

We also identified another group of virtually normal proteins in AP-free patients with SZ or BD but were downregulated in patients with either disorder on APs. These mainly included proteins involved in the immune system, including PIANP, TNFRSF17, and NCAM1. These alterations are probably more linked to the effects of the AP than to the disorders themselves. However, contrary to our findings, NCAM1 was previously reported to be elevated in drug-free patients with BD, with a positive correlation with the severity of mania (39). In previous proteomic studies about SZ or BD, most reported potential biomarkers were immune-related, including pro-inflammatory cytokines, Complement C3, and MIF (10, 16, 40). However, most studies included patients on treatment, thus making it difficult to disentangle the effects of the disorder from the medication's effects.

We also found that prolactin (PRL) was upregulated in patients with SZ and BD, both on treatment and AP-free. High PRL levels in patients with SZ or BD are often attributed to APs. Nevertheless, there have been reports of high prolactin levels in drug-naive patients with SZ or other psychotic disorders (41), as well as in BD (42). The data on PRL in drug-free or drug-naïve patients are still equivocal (43). However, a meta-analysis of the studies on long-acting injectable (LAI) and second-generation oral antipsychotics reported better clinical outcomes with LAIs but higher PRL level and side effects (44). Many hypotheses have been proposed, including relationships between PRL and dopamine, links between PRL and stress, effects of the Thyroid Stimulating Hormone (4) axis on PRL, as well as pro-inflammatory and detrimental cognitive effects of PRL (42, 45).

### Enrichment Analysis

The most substantial enrichment of proteins from the differentiation of neurons and the central nervous system's morphology were detected in the downregulated fraction of proteins in patients with SZ. This enrichment seems stronger in patients with SZ on APs than in those drug-free. Proteomic studies in blood previously showed that many of the differentially expressed proteins in SZ had roles in neuronal differentiation and maturation, synaptic plasticity, and neurites outgrowth (46). Similarly, GWAS identified risk variants associated with early brain development and synaptogenesis (47). Previous studies reported that APs partly “repair” the neuronal abnormalities associated with SZ and promote neuronal differentiation (48, 49). Another possibility might be that the AP-free group had a “milder” form of SZ in the first place, where the neurodevelopmental abnormalities might have been less prominent.

We also found disease traits of vascular dysfunction (cerebrovascular dysfunction, the permeability of vasculature, and blood pressure) in patients on APs (both with SZ and BD) and in AP-free patients with BD. Signs of vascular dysfunction, including endothelial dysfunction, have been previously found in patients with BD. Compared to lithium or anticonvulsant use, AP use was also found to accentuate these alterations (50). In our enrichment analysis, most of these alterations seem to be similar on a large scale in patients on APs than in those who were drug-free with certain exceptions, including NTRK2, CNTN1, ROBO2, NOTCH1, AKT1. Still, a larger sample size might have allowed us to highlight more differences between untreated patients and patients on APs.

### Associations With the Metabolic Syndrome

We found that metabolic syndrome-associated clinical variables were more associated with AP use than with the presence of SZ or BD. Even though drug-naïve patients with SZ might also display a higher prevalence of metabolic syndrome than the general population (51), most of the metabolic risk in patients with SZ and BD has been attributed to the AP use (52). In SZ, proteins involved in glucose homeostasis and lipid metabolism have been repeatedly reported to be altered. Among the proteins involved in appetite glucose metabolism, we found several associated with AP use, including IL15RA, TNFRSF1B, TNFRSF1A, FGF19, APOM, PTPN11. While previous studies did not report the same proteins, they found that other proteins associated with appetite regulation and glucose homeostasis, mainly leptin, insulin, and C-peptide, were differentially elevated in patients with SZ (53–55). We also found that certain blood pressure annotated proteins, including TNFRSF1B, ANGPT2, PRL, PTHLH, and CD36, were associated with AP use. Some of these proteins have already been linked to AP use in previous studies. Indeed, the link between AP use and increased PRL has been well-established, and high PRL has been associated with hypertension (56). Although we did not find studies linking TNFRSF1B to AP use *per se*, a previous GWAS study linked TNFRSF1B to tardive dyskinesia, a known side effect of APs (57). In addition, olanzapine use has also been associated with CD36 overexpression (58).

Our results indicated that proteins mostly involved in the immune function (such as NCAM1, PIANP, and the C5 factor of the Complement) were strongly associated with the BMI and waist circumference, similarly in HCs, AP-free, and AP-treated patients with BD or SZ. A plausible explanation could be that these proteins are more associated with obesity than psychiatric disorders or AP use. It is also in line with the growing body of evidence highlighting the interrelationships between obesity, metabolic syndrome, and immune dysfunction (59). Proteins differentially expressed in patients with SZ or BD might not be directly linked to the psychiatric condition itself or the medication's effects but rather to the associations between these psychiatric disorders and metabolic syndrome.

### Biomarker Identification

We found that it was possible to predict SZ using only two biomarkers (PRL and MRC2) with a sensitivity of 0.97 and specificity of 0.75. Previously, PRL was reported to be upregulated in patients with SZ (55), including drug-naive patients (41). MRC2 is a C-type lectin primarily present on the surface of macrophages and microglial cells. In a meta-analysis using the Detecting Association with Network (DAWN) framework to identify SZ risk genes, the MRC2 gene was a primary risk gene (60). Also, in a GWAS, the MRC2 gene was associated with “psychosis proneness” (61).

Prediction of BD was much less accurate, even with ten biomarkers. This is may be due to the considerable heterogeneity of the disorder, possibly blurring the associations between biomarkers and certain types or subtypes of BD (62). Indeed, a multiplex blood-based proteomic study showed that growth differentiation factor 15 (GDF-15, also an immune modulator), retinol-binding protein 4 (RBP-4, a transporter of retinol from the liver to peripheral tissues), and transthyretin (TTR, a transporter of T4 across the blood-brain barrier) were good predictors of type I BD with ROC-AUC > 0.8 (63). A quantitative mass-spectrometry-based proteomics study comparing patients with SZ to patients with BD found that ankyrin repeat domain-containing protein 12 (ANKRD12, a protein probably involved in gene regulation) was higher in patients with SZ (8).

### Strengths and Limitations

This study was one of few studies to include both treated and AP-free patients with SZ and BD, and HCs, to help distinguish the possible effects of the conditions from the potential effects of AP. To the best of our knowledge, this is the first proteomic study performed in the Arab region. However, a few limitations are to be acknowledged. First, the group of AP-free patients included patients who had been off AP medication for at least 6 months, rather than drug-naïve patients. Even though most of the potential effects of the AP should have subsided after this period, the long-term effects of APs on specific circulating proteins cannot be ruled out. Second, we assessed proteins in the plasma where levels do not necessarily reflect those at the cellular and tissue level in the brain (46). However, most previous proteomic studies in SZ and BD used blood, serum, or plasma. This is probably because blood-based biomarkers can be more clinically useful than other biofluids since these fluids are more easily accessible, less invasive to obtain, and simpler to standardize (5, 6). Third, the sample size of some of the groups, especially those AP-free, might be small, and thus some comparisons did not have enough power to reach significance. Hence, the results for the proteins for which we did not find any significant differences between groups should be interpreted with caution, given the possibility of a type II error due to the small sample size.

In conclusion, our results support plasma proteomic's utility to explore the pathophysiology and treatment of SZ and BP. Our study's design assessing the proteome signatures in the patients on and off AP, together with the comparisons with another HC group, allowed the identification of the processes involved in the disease itself vs. the APs effects. The protein changes (with and without AP) are implicated in the neurodevelopmental aspects of these disorders and might contribute to the development of better treatments for SZ and BP. The AP-induced proteome modifications, independent of the disease, highlight the clinical findings concerning the high prevalence of metabolic syndrome and cardiovascular diseases in these patients.

## Data Availability Statement

The original data, as received from SOMAscan (Boulder, CO), was deposited at the Dryad Digital Repository under https://doi.org/10.5061/dryad.p8cz8w9sc. Further enquires can be directed toward the corresponding author.

## Ethics Statement

The Institutional Review Boards approved the study at Hamad Medical Corporation (HMC) and Weill Cornell Medicine in Qatar (IRB reference number: 14-00106). All participants signed a written informed consent after explaining all the procedures of the study. All study procedures were carried out according to the Helsinki Declaration of 1975. The patients/participants provided their written informed consent to participate in this study.

## Author Contributions

HA-A designed and supervised all the aspects of the study. RE, FS, KS, and HA-A designed the proteomics study and contributed to the implementation and analysis of results. SG contributed to the design and supervised the performance at the clinical site. RC and NK implemented the study, collected the clinical data, and processed the samples. RE and HS executed the proteomic measurements. RE performed the analysis, and wrote the first draft of methods and results. SO reviewed the literature and finalized the first draft of the manuscript. All authors contributed to the writing of the manuscript. They also reviewed and approved the final version before submission.

## Funding

This study was part of a larger project funded solely by Qatar National Research Fund (QNRF) www.qnrf.org granted to HA-A (NPRP 4–268–3-085). QNRF did not have any additional role in the study design, data collection and analysis, interpretation of data, publication decision, or manuscript preparation.

## Conflict of Interest

SO and SG were employed by Hamad Medical Corporation. The remaining authors declare that the research was conducted in the absence of any commercial or financial relationships that could be construed as a potential conflict of interest.

## Publisher's Note

All claims expressed in this article are solely those of the authors and do not necessarily represent those of their affiliated organizations, or those of the publisher, the editors and the reviewers. Any product that may be evaluated in this article, or claim that may be made by its manufacturer, is not guaranteed or endorsed by the publisher.

## Supplementary Material

Supplementary Figure 1 Heatmap showing standardized log2 protein level ratios for most significant proteins (*post-hoc* FDR < 0.01) of SZ and BD patients (+/−AP) compared to HC. Right site indicates disease and function term annotation using Ingenuity Pathways Analysis software. Asterisk indicates *P*-value significance level (“^***^” 0 - 0.001, “^**^” 0.001 - 0.01, “^*^” 0.01 - 0.05, “.” 0.05 - 0.1).

Supplementary Figure 2 Clinical variables and their associations with differentially abundant proteins. A Boxplot indicating the distribution of BMI, waist circumference, systolic, and diastolic blood pressure across conditions. The ANCOVA *P*-values and *post-hoc P*-values are displayed for group comparisons correcting for gender and age.

Supplementary Table 1 Differential analysis of proteins quantified with the SOMAscan assay. A complete list of proteins' ANCOVA and *post hoc* test results.

Supplementary Table 2 Circulating protein biomarkers reported in previous plasma or serum-based biomarker studies in BD and SZ.

Supplementary Table 3 Summary of receiver operating characteristic (ROC) analysis for the diagnosis of SZ and BD.

Supplementary Table 4 Risk factors of metabolic syndrome.

Supplementary Table 5 A complete list of protein associations and interactions with clinical variables.
