# Supplementary figures and images for: Proteomic Analysis of Plasma Markers in Patients Maintained on Antipsychotics: Comparison to Patients Off Antipsychotics and Normal Controls
Source: Front Psychiatry. 2022 Apr 25;13:809071. doi: 10.3389/fpsyt.2022.809071 (PMC9081931; doi:10.3389/fpsyt.2022.809071)

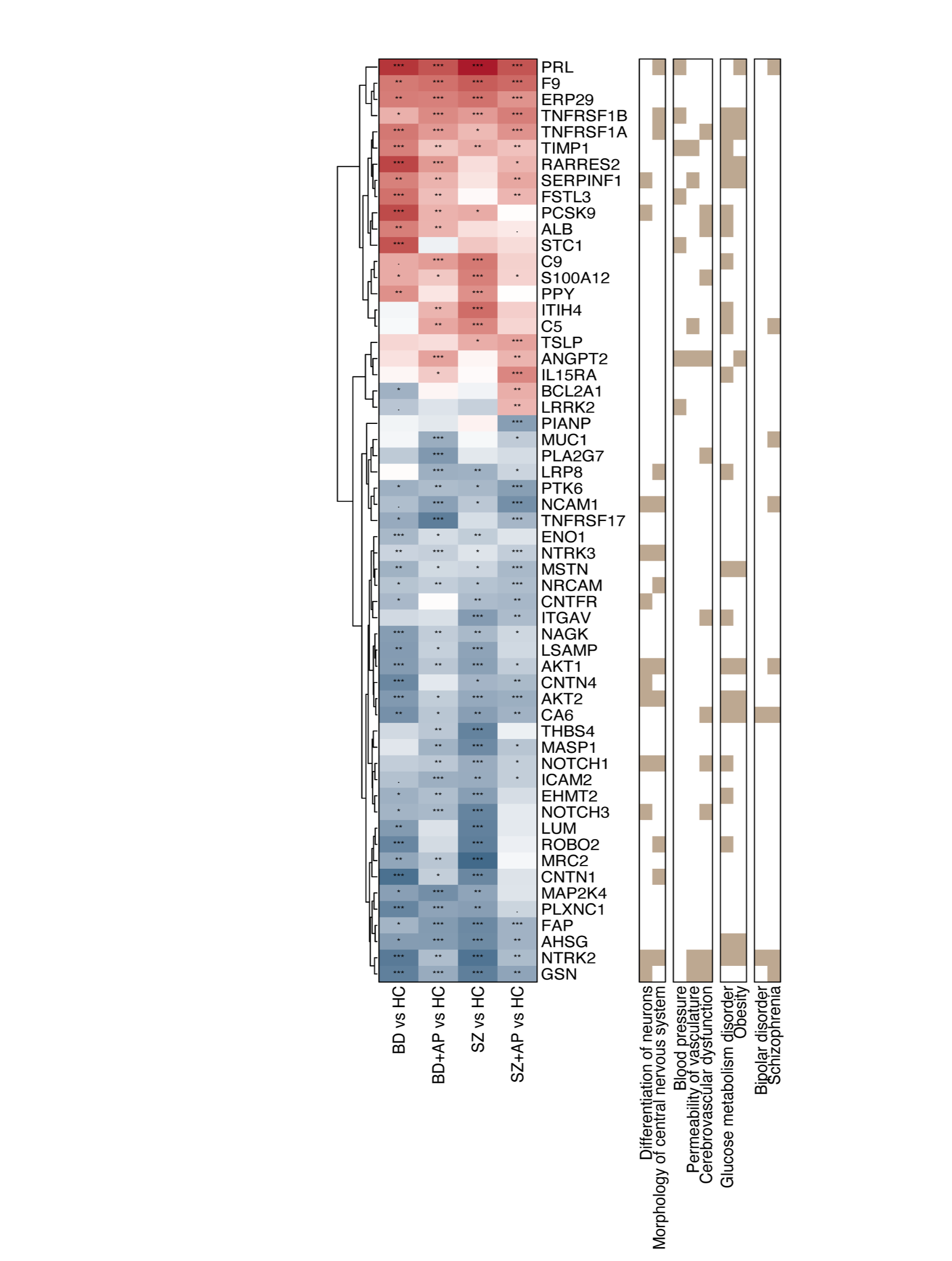

Supplement: Supplementary Figure 1 — Heatmap showing standardized log2 protein level ratios for most significant proteins (post-hoc FDR < 0.01) of SZ and BD patients (+/−AP) compared to HC. Right site indicates disease and function term annotation using Ingenuity Pathways Analysis software. Asterisk indicates P-value significance level (“***” 0 - 0.001, “**” 0.001 - 0.01, “*” 0.01 - 0.05, “.” 0.05 - 0.1). [file Image_1.TIFF]

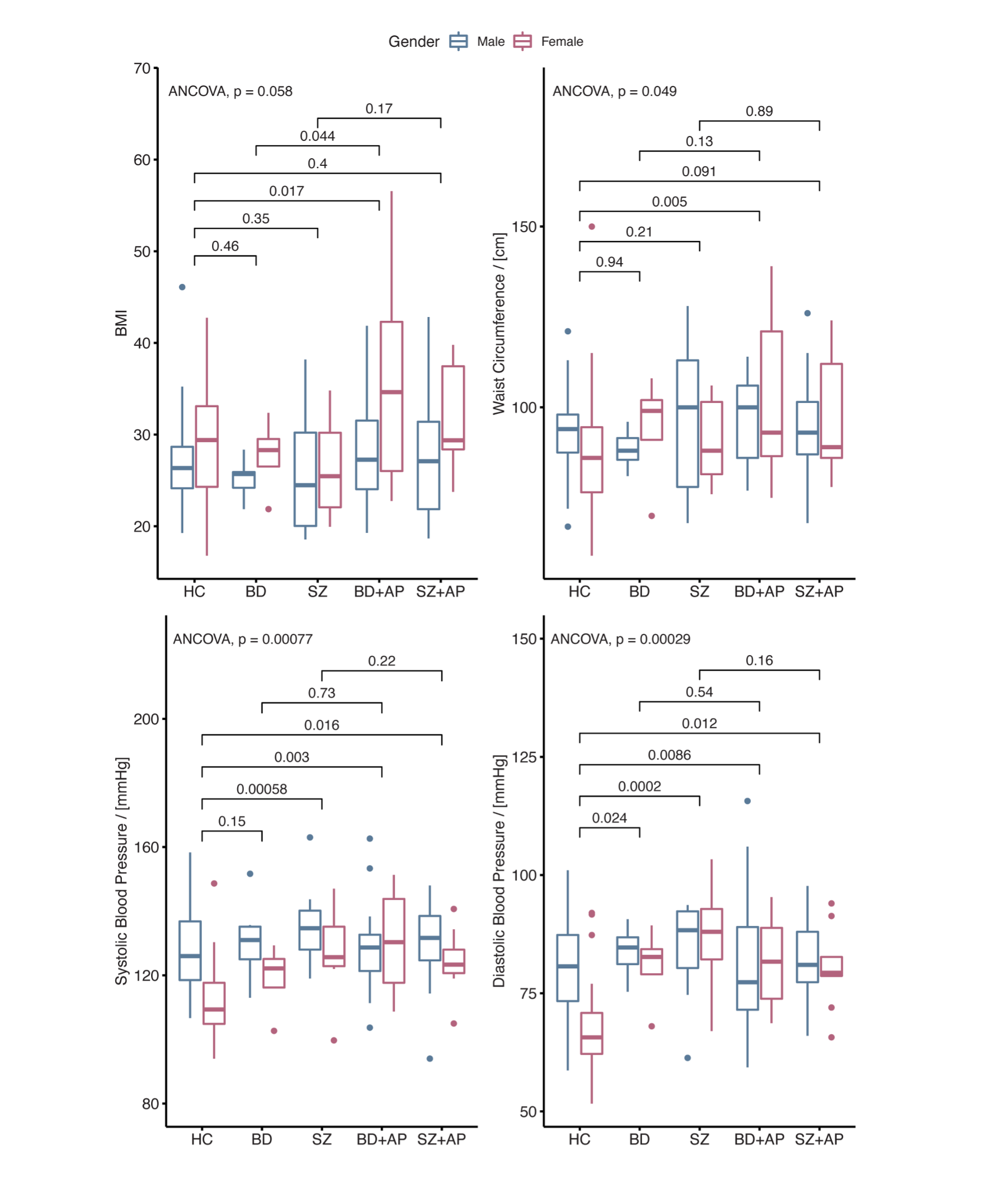

Supplement: Supplementary Figure 2 — Clinical variables and their associations with differentially abundant proteins. A Boxplot indicating the distribution of BMI, waist circumference, systolic, and diastolic blood pressure across conditions. The ANCOVA P-values and post-hoc P-values are displayed for group comparisons correcting for gender and age. [file Image_2.TIFF]
