# Supplementary material for: Proteomic Analysis of Plasma Markers in Patients Maintained on Antipsychotics: Comparison to Patients Off Antipsychotics and Normal Controls
Source: Front Psychiatry. 2022 Apr 25;13:809071. doi: 10.3389/fpsyt.2022.809071 (PMC9081931; doi:10.3389/fpsyt.2022.809071)
Supplement: Supplementary file 8 [file Data_Sheet_1.PDF]

ASSOCIATION

	SOMAscan SeqId	SOMAscan Target	UniProt AC	Gene name	BMI P	BMI beta	Waist measurement P	Waist measurement beta	Systolic blood pressure P	Systolic blood pressure beta	Diastolic blood pressure P	Diastolic blood pressure beta	HDL P	HDL beta	LDL P	LDL beta	Triglycerides P	Triglycerides beta	Fasting blood glucose P	Fasting blood glucose beta
1	10336-3_3	CHIP	Q9UNE7	STUB1	7.84E-01	-0.0026	7.76E-01	-0.0011	4.60E-01	-0.0032	7.47E-01	-0.0017	1.08E-01	0.2823	5.32E-01	0.0423	8.66E-01	-0.0111	6.43E-01	0.0209
2	10337-83_3	CEBPB	P17676	CEBPB	3.54E-01	0.0090	1.04E-01	0.0066	1.18E-01	-0.0069	6.56E-02	-0.0101	2.05E-01	0.2294	8.32E-01	-0.0148	3.77E-01	-0.0598	9.98E-02	0.0762
3	10339-48_3	NSE	P09104	ENO2	1.78E-02	-0.0144	7.76E-02	-0.0045	6.65E-01	0.0012	2.68E-01	0.0039	7.38E-01	0.0383	8.01E-01	0.0111	9.10E-01	0.0048	2.64E-01	-0.0327
4	10342-55_3	PIAS4	Q8N2W9	PIAS4	6.54E-01	0.0047	3.97E-01	0.0037	2.96E-01	-0.0050	9.04E-01	0.0007	8.35E-01	0.0407	9.26E-01	-0.0070	3.25E-01	-0.0718	1.68E-01	0.0689
5	10344-334_3	IL-10 Ra	Q13651	IL10RA	1.36E-02	0.0226	8.91E-02	0.0066	1.66E-01	0.0058	7.20E-01	-0.0019	6.44E-01	-0.0799	9.79E-01	0.0018	1.86E-01	0.0852	6.61E-01	0.0194
6	10346-5_3	STAT3	P40763	STAT3	3.39E-01	0.0066	9.52E-01	-0.0002	1.84E-02	-0.0073	5.27E-01	-0.0025	1.65E-01	0.1785	2.60E-01	0.0557	1.87E-01	-0.0632	5.68E-01	0.0188
7	10351-51_3	IRF1	P10914	IRF1	2.31E-01	-0.0111	5.06E-01	-0.0026	3.93E-01	0.0036	9.51E-01	0.0003	8.15E-01	-0.0408	4.08E-01	-0.0554	2.34E-01	0.0774	8.59E-01	0.0079
8	10356-21_3	c-Jun	P05412	JUN	3.73E-01	-0.0084	3.75E-01	-0.0035	6.57E-01	-0.0019	3.34E-01	-0.0052	3.83E-01	0.1538	1.40E-01	-0.0997	1.62E-01	0.0920	1.48E-01	0.0653
9	10358-33_3	Mcl-1	Q07820	MCL1	7.24E-01	0.0039	5.11E-01	0.0031	5.48E-01	0.0030	9.82E-01	-0.0001	3.73E-02	0.4275	2.58E-01	0.0895	4.97E-01	-0.0523	5.19E-01	0.0341
10	10361-25_3	OAS1	P00973	OAS1	5.24E-02	0.0196	6.87E-02	0.0078	8.70E-01	-0.0008	9.17E-01	0.0006	8.67E-01	-0.0318	6.37E-01	0.0345	8.86E-01	0.0102	9.10E-01	-0.0055
11	10362-35_3	c-Myc	P01106	MYC	8.02E-01	0.0025	4.69E-01	0.0030	4.04E-01	-0.0037	1.03E-01	-0.0092	4.13E-01	0.1512	1.28E-02	0.1754	4.60E-01	-0.0509	3.98E-01	-0.0400
12	10363-13_3	SMAD3	P84022	SMAD3	1.49E-02	-0.0145	4.20E-02	-0.0051	7.91E-01	-0.0007	8.45E-01	0.0007	3.92E-01	0.0960	9.58E-01	-0.0023	9.90E-02	0.0689	7.48E-01	0.0093
13	10364-6_3	SMAD2	Q15796	SMAD2	8.76E-02		7.85E-02	-0.0045	9.81E-01	-0.0001	9.39E-01	-0.0003	9.34E-01	-0.0095	6.41E-02	0.0814	7.95E-02	0.0749	1.49E-01	-0.0423
14	10365-132_3	IL-23	P29460, Q9N[illegible]	IL12B IL23A	2.20E-01	0.0101	4.27E-01	0.0027	7.11E-01	0.0014	7.63E-01	0.0014	1.19E-01	-0.2393	8.12E-01	-0.0141	4.39E-01	0.0444	1.56E-01	0.0558
15	10366-11_3	PDGFRA	P16234	PDGFRA	1.34E-02	-0.0199	1.17E-02	-0.0086	1.55E-01	-0.0052	4.91E-02	-0.0091	6.03E-01	0.0790	6.66E-01	0.0252	2.01E-01	-0.0725	5.86E-01	-0.0212
16	10367-62_3	IL-12	P29459, P2[illegible]	IL12A IL12B	8.79E-01	-0.0017	7.77E-01	0.0013	3.19E-01	0.0051	9.41E-01	-0.0005	8.40E-01	0.0424	9.97E-01	0.0003	3.67E-01	-0.0705	6.98E-01	-0.0208
17	10370-21_3	STAT1	P42224	STAT1	1.27E-01	-0.0105	1.15E-01	-0.0046	4.11E-01	-0.0026	7.63E-01	-0.0012	9.58E-01	0.0068	6.53E-01	-0.0223	9.87E-01	-0.0008	2.95E-01	-0.0347
18	10372-18_3	STAT6	P42226	STAT6	9.20E-01	-0.0010	6.09E-01	-0.0021	2.39E-01	0.0052	1.54E-01	0.0079	6.28E-01	0.0877	2.79E-01	-0.0753	7.86E-01	0.0183	2.83E-01	-0.0497
19	10990-21_3	LRRK2	Q5S007	LRRK2	2.37E-01	-0.0129	3.99E-01	-0.0039	2.69E-01	-0.0055	4.08E-01	-0.0052	6.92E-01	0.0812	5.87E-01	-0.0428	2.35E-01	-0.0907	1.22E-01	0.0810
20	11067-13_3	Osteocalcin	P02818	BGLAP	6.18E-01	-0.0053	6.74E-01	-0.0019	9.84E-01	-0.0001	4.28E-01	0.0048	3.92E-01	-0.1711	2.13E-01	0.0956	5.89E-01	0.0403	1.08E-01	-0.0821
21	11071-1_3	IL-5	P05113	IL5	1.16E-01	0.0168	7.21E-01	0.0016	1.02E-01	0.0079	9.89E-01	-0.0001	8.70E-01	0.0327	6.68E-01	-0.0330	8.06E-01	-0.0184	3.67E-02	-0.1067
22	11081-1_3	GPDA	P21695	GPD1	6.01E-04	0.0257	3.23E-03	0.0093	2.60E-01	0.0039	9.81E-02	0.0072	7.20E-01	-0.0510	1.69E-01	0.0751	7.47E-01	0.0172	7.36E-01	0.0123
23	11089-7_3	IgA	P01876 P01[illegible]	IGHA1 IGHA[illegible]	2.68E-02	0.0256	3.88E-01	0.0042	8.95E-01	-0.0007	4.14E-01	-0.0054	2.61E-01	-0.2444	6.27E-01	-0.0407	7.00E-01	-0.0313	2.73E-02	0.1224
24	11094-104_3	LPPL	Q05315	CLC	2.70E-01	0.0117	7.92E-01	0.0012	9.60E-01	0.0002	1.71E-01	-0.0083	7.43E-01	-0.0651	6.18E-02	0.1419	2.98E-01	-0.0770	9.47E-01	0.0034
25	11096-57_3	HEMK2	Q9Y5N5	N6AMT1	7.14E-01	-0.0033	7.38E-01	0.0013	3.23E-01	-0.0040	8.71E-01	0.0008	8.36E-03	0.4367	7.91E-01	-0.0171	3.13E-01	-0.0628	9.27E-01	-0.0039
[illegible]	[illegible]	[illegible]	[illegible]	[illegible]	[illegible]	[illegible]	[illegible]	[illegible]	[illegible]	[illegible]	[illegible]	[illegible]	[illegible]	[illegible]	[illegible]	[illegible]	[illegible]	[illegible]	[illegible]	[illegible]

INTERACTION

[illegible]

228	2763-66_2	FGF-20	Q9NP95	FGF20	8.34E-01	-0.0018
229	2764-20_2	FGF9	P31371	FGF9	7.86E-02	0.0184
230	2765-4_3	GDF-11/8	O95390 O14	GDF11 MSTI	2.44E-02	-0.0227
231	2768-56_2	Hemopexin	P02790	HPX	3.38E-02	0.0251
232	2769-3_2	HIV-2 Rev	P18093	Human-virus	8.23E-02	0.0162
233	2770-51_2	I-309	P22362	CCL1	3.09E-01	-0.0095
234	2771-35_2	IGFBP-1	P08833	IGFBP1	8.65E-11	-0.0695
235	2773-50_2	IL-10	P22301	IL10	1.21E-01	0.0163
236	2774-10_3	IL-16	Q14005	IL16	6.49E-01	-0.0051
237	2775-54_2	IL-17F	Q96PD4	IL17F	7.17E-01	-0.0042
238	2778-10_2	IL-22	Q9GZX6	IL22	4.10E-01	-0.0092
239	2780-35_2	Lactoferrin	P02788	LTF	1.82E-02	0.0259
240	2781-63_2	LAG-1	Q8NHW4	CCL4L1	2.33E-01	0.0092
241	2783-18_2	LD78-beta	P16619	CCL3L1	4.85E-03	0.0264
242	2785-15_2	MCP-2	P80075	CCL8	5.44E-02	0.0192
243	2788-55_1	MMP-3	P08254	MMP3	5.93E-01	-0.0056
244	2789-26_2	MMP-7	P09237	MMP7	3.45E-01	-0.0092
245	2790-54_2	NAP-2	P02775	PPBP	8.07E-02	0.0177
246	2794-60_2	SOD	P00441	SOD1	3.62E-01	0.0057
247	2796-62_2	Fibrinogen	P02671 P02	FGA FGB FG	3.77E-01	0.0100
248	2797-56_2	Apo B	P04114	APOB	3.41E-01	-0.0106
249	2805-6_2	ACE2	Q9BYF1	ACE2	9.65E-01	-0.0004
250	2806-49_2	Activin RIB	P36896	ACVR1B	5.62E-01	-0.0053
251	2809-25_2	ADAMTS-4	O75173	ADAMTS4	7.69E-02	0.0185
252	2811-27_1	Angiopoietin	Q15389	ANGPT1	9.10E-02	0.0177
253	2813-11_2	ART	O00253	AGRP	8.42E-02	-0.0093
254	2816-50_2	BCAM	P50895	BCAM	6.36E-01	-0.0041
255	2819-23_2	Cadherin-5	P33151	CDH5	7.21E-02	-0.0192
256	2822-56_2	CD97	P48960	CD97	4.63E-02	-0.0232
257	2823-7_1	COMMD7	Q86VX2	COMMD7	5.86E-02	-0.0144
258	2826-53_2	EDA	Q92838	EDA	1.45E-02	-0.0173
259	2827-23_2	Fractalkine/	P78423	CX3CL1	3.04E-06	-0.0450
260	2828-82_2	HAI-1	O43278	SPINT1	4.46E-02	-0.0146
261	2829-19_2	IL-27	Q8NEV9 Q1	IL27 EBI3	3.67E-01	0.0086
262	2831-29_1	Kallikrein 11	Q9UBX7	KLK11	3.76E-02	-0.0144
263	2833-20_1	Kallikrein 4	Q9Y5K2	KLK4	3.61E-01	0.0086
264	2834-54_1	kallikrein 8	O60259	KLK8	2.18E-01	-0.0072
265	2835-1_4	Ku70	P12956	XRCC6	3.56E-01	0.0083
266	2836-68_2	Lipocalin 2	P80188	LCN2	5.80E-01	-0.0066
267	2837-3_2	Met	P08581	MET	1.95E-03	-0.0294
268	2838-53_1	MMP-17	Q9ULZ9	MMP17	1.59E-01	-0.0155
269	2839-2_1	OX40 Ligand	P23510	TNFSF4	9.06E-02	-0.0175
270	2841-13_2	sFRP-3	Q92765	FRZB	3.90E-01	-0.0086
271	2843-13_2	SPINT2	O43291	SPINT2	6.45E-01	0.0048
272	2844-53_2	sTie-1	P35590	TIE1	3.46E-01	-0.0100
273	2846-24_2	Ubiquitin+1	P62979	RPS27A	5.91E-01	-0.0030
274	2848-2_2	WIF-1	Q9Y5W5	WIF1	4.03E-04	-0.0312
275	2849-49_1	AIF1	P55008	AIF1	1.53E-01	-0.0129
276	2851-63_3	C5a	P01031	C5	9.24E-16	0.0834
277	2853-68_2	CHK1	O14757	CHEK1	4.58E-01	-0.0077
278	2855-49_2	ERK-1	P27361	MAPK3	3.53E-01	-0.0061
279	2857-70_2	Glucocortico	P04150	NR3C1	7.56E-01	-0.0033
280	2858-29_2	Hat1	O14929	HAT1	8.18E-01	0.0016
281	2859-69_2	HDAC8	Q9BY41	HDAC8	9.44E-02	-0.0179
282	2860-19_2	Karyopherin-	P52292	KPNA2	7.84E-01	0.0022
283	2864-2_3	MEK1	Q02750	MAP2K1	5.87E-01	0.0052
284	2865-77_2	MOZ	Q92794	KAT6A	8.02E-01	-0.0026
285	2869-68_3	PKC-D	Q05655	PRKCD	6.49E-01	0.0040
286	2870-29_2	RAC1	P63000	RAC1	6.21E-03	-0.0129
287	2871-73_2	RAD51	Q06609	RAD51	3.31E-03	-0.0266
288	2875-15_2	TBP	P20226	TBP	2.94E-01	-0.0092
289	2876-74_2	Topoisomera	P11387	TOP1	3.54E-01	-0.0067
290	2877-3_1	UBC9	P63279	UBE2I	7.89E-01	0.0012
291	2878-66_2	YES	P07947	YES1	8.79E-02	-0.0170
292	2879-9_2	a1-Antichym	P01011	SERPINA3	1.11E-01	-0.0184
293	2888-49_2	C7	P10643	C7	6.00E-05	-0.0441
294	2889-37_2	Cardiotrophi	Q16619	CTF1	5.98E-01	0.0043
295	2890-59_2	CCL28	Q9NRJ3	CCL28	9.02E-02	-0.0202
296	2891-1_3	CD22	P20273	CD22	1.09E-02	-0.0228
297	2900-53_3	HCC-1	Q16627	CCL14	2.63E-01	0.0125
298	2906-55_3	IL-4	P05112	IL4	1.14E-01	0.0117
299	2911-27_2	Midkine	P21741	MDK	2.01E-01	0.0144
300	2913-1_2	MPIF-1	P55773	CCL23	7.50E-01	0.0035
301	2915-6_2	PCNA	P12004	PCNA	2.96E-03	0.0316
302	2917-3_2	sRANKL	O14788	TNFSF11	5.39E-01	-0.0060
303	2925-9_1	PAI-1	P05121	SERPINE1	4.70E-05	0.0452
304	2937-10_2	Apo E3	P02649	APOE	3.56E-01	-0.0105
305	2938-55_2	Apo E4	P02649	APOE	4.46E-01	-0.0089
306	2939-10_2	Artemin	Q5T4W7	ARTN	4.01E-01	0.0084
307	2942-50_2	Cytochrome	P99999	CYCS	3.28E-01	-0.0076
308	2943-5_2	Cytochrome	P08684	CYP3A4	7.10E-01	0.0035
309	2944-66_2	DAN	P41271	NBL1	6.72E-01	0.0036
310	2945-25_1	ER	P03372	ESR1	9.15E-01	0.0008
311	2946-52_2	Factor D	P00746	CFD	3.90E-01	0.0086
312	2948-58_2	Growth horn	P10912	GHR	2.35E-08	0.0593
313	2949-6_2	GX	O15496	PLA2G10	5.67E-03	-0.0295
314	2950-57_2	IGFBP-4	P22692	IGFBP4	1.42E-02	0.0271
315	2952-75_2	IGF-I	P05019	IGF1	1.15E-04	-0.0423
316	2953-31_2	Luteinizing h	P01215, P01	CGA LHB	3.69E-02	-0.0232
317	2960-66_2	Properdin	P27918	CFP	7.31E-02	0.0078
318	2961-1_2	Protein C	P04070	PROC	1.45E-01	-0.0169
319	2962-50_2	PTHrP	P12272	PTHLH	2.32E-01	-0.0122
320	2966-65_2	SCGF-beta	Q9Y240	CLEC11A	3.82E-01	-0.0099
321	2967-8_1	VCAM-1	P19320	VCAM1	4.10E-01	-0.0091
322	2968-61_1	TNFSF15	O95150	TNFSF15	9.15E-01	0.0007
323	2969-11_1	ALK-1	P37023	ACVRL1	1.39E-01	-0.0164
324	2970-60_2	AREG	P15514	AREG	1.10E-01	-0.0084
325	2972-57_2	BMP-7	P18075	BMP7	5.95E-01	-0.0039
326	2973-15_2	CD36 ANTIG	P16671	CD36	2.05E-01	0.0139
327	2974-61_2	contactin-1	Q12860	CNTN1	1.18E-03	-0.0374
328	2975-19_2	CTGF	P29279	CTGF	3.18E-02	0.0172
329	2976-58_2	Desmoglein-	Q02413	DSG1	9.99E-02	-0.0156
330	2977-7_2	EDAR	Q9UNE0	EDAR	2.69E-01	-0.0105
331	2979-8_2	ENA-78	P42830	CXCL5	4.21E-03	0.0265
332	2981-9_3	ESAM	Q96AP7	ESAM	3.86E-01	-0.0099
333	2982-82_2	Galectin-4	P56470	LGALS4	7.54E-01	0.0029
334	2985-35_1	Gro-a	P09341	CXCL1	7.70E-01	-0.0030
335	2986-49_2	Gro-b/g	P19876 P19	CXCL3 CXCL2	3.22E-01	0.0089
336	2987-37_3	Histone H1.2	P16403	HIST1H1C	1.33E-01	-0.0123
337	2988-57_2	ICOS	Q9Y6W8	ICOS	7.93E-01	-0.0026
338	2991-9_2	IL-1 sRI	P14778	IL1R1	6.34E-06	-0.0220
339	2992-59_2	IL-17 sR	Q96F46	IL17RA	2.42E-04	-0.0423
340	2993-1_2	IL-18 Rb	O95256	IL18RAP	7.47E-01	0.0038
341	2994-71_2	IL-1Rrp2	Q9HB29	IL1RL2	4.51E-01	-0.0079
342	2997-8_1	JAM-B	P57087	JAM2	8.32E-03	-0.0269
343	2998-53_2	JAM-C	Q9BX67	JAM3	1.62E-01	-0.0103
344	2999-6_2	LSAMP	Q13449	LSAMP	7.93E-09	-0.0559
345	3000-66_1	MBL	P11226	MBL2	7.43E-01	-0.0035
346	3003-29_2	NKp30	O14931	NCR3	9.37E-01	0.0009
347	3004-67_2	PD-L2	Q9BQ51	PDCD1LG2	3.53E-02	-0.0163
348	3005-5_2	PTP-1B	P18031	PTPN1	1.87E-01	-0.0122
349	3007-7_2	Siglec-9	Q9Y336	SIGLEC9	5.32E-01	0.0071
350	3009-3_2	TGF-b R III	Q03167	TGFBR3	7.97E-04	-0.0342
351	3010-53_2	TSLP	Q969D9	TSLP	9.38E-02	0.0128
352	3022-4_2	CTLA-4	P16410	CTLA4	4.21E-01	0.0077
353	3024-18_2	a2-Antiplasn	P08697	SERPINF2	3.40E-01	-0.0103
354	3025-50_1	bFGF	P09038	FGF2	8.23E-01	-0.0025
355	3026-5_2	Calpastatin	P20810	CAST	2.63E-02	-0.0252
356	3028-36_2	Ck-b-8-1	P55773	CCL23	4.02E-01	0.0089
357	3029-52_2	DC-SIGN	Q9NNX6	CD209	8.43E-01	0.0023
358	3030-3_2	DC-SIGNR	Q9H2X3	CLEC4M	5.04E-01	0.0070
359	3032-11_2	FSH	P01215, P01	CGA FSHB	2.23E-02	-0.0245
360	3033-57_1	Galectin-2	P05162	LGALS2	6.45E-01	0.0049
361	3034-1_2	GFAP	P14136	GFAP	7.48E-01	-0.0036
362	3035-80_2	IL-19	Q9UHD0	IL19	7.84E-03	-0.0291
363	3037-62_1	IL-1b	P01584	IL1B	6.10E-01	0.0040
364	3038-9_2	I-TAC	O14625	CXCL11	5.84E-01	0.0062
365	3040-59_1	MIP-1a	P10147	CCL3	1.23E-02	0.0281
366	3041-55_2	MRC2	Q9UBG0	MRC2	5.98E-01	-0.0055
367	3042-7_2	Myoglobin	P02144	MB	5.79E-01	-0.0060
368	3043-49_2	ON	P09486	SPARC	4.15E-02	0.0220
369	3044-3_2	PARC	P55774	CCL18	5.11E-04	0.0385
370	3045-72_2	PTN	P21246	PTN	8.57E-01	-0.0020
371	3046-31_1	resistin	Q9HD89	RETN	8.28E-01	-0.0025
372	3049-61_2	Trypsin	P07477	PRSS1	9.78E-03	-0.0286
373	3050-7_2	vWF	P04275	VWF	4.39E-01	0.0091
374	3052-8_2	Fas ligand, s	P48023	FASLG	7.13E-01	-0.0032
375	3053-49_2	Flt3 ligand	P49771	FLT3LG	6.39E-01	0.0042
376	3054-3_2	Haptoglobin,	P00738	HP	4.38E-03	0.0295
377	3055-54_2	IL-4 sR	P24394	IL4R	3.08E-01	-0.0087
378	3056-11_1	NKG2D	P26718	KLRK1	1.68E-01	-0.0149
379	3057-55_1	WISP-1	O95388	WISP1	6.46E-01	-0.0044
380	3059-50_2	BAFF	Q9Y275	TNFSF13B	2.95E-01	0.0077
381	3060-43_2	C9	P02748	C9	7.02E-02	0.0209
382	3061-61_2	Cathepsin B	P07858	CTSB	6.98E-01	0.0043
383	3065-65_1	FGF-5	P12034	FGF5	7.32E-01	0.0035
384	3066-12_1	Galectin-3	P17931	LGALS3	2.30E-01	0.0130
385	3067-67_1	GDF-9	O60383	GDF9	9.99E-01	0.0000
386	3069-52_3	IgM	P01871	IGHM IGJ IG	2.03E-02	-0.0262
387	3070-1_2	IL-2	P60568	IL2	2.83E-03	0.0148
388	3072-4_2	IL-13	P35225	IL13	8.45E-02	-0.0173
389	3073-51_2	IL-18 BPa	O95998	IL18BP	1.87E-02	-0.0223
390	3074-6_2	LBP	P18428	LBP	4.38E-02	0.0225
391	3077-66_2	Coagulation	P00742	F10	1.20E-01	0.0180
392	3078-1_2	PlGF	P49763	PGF	4.15E-01	0.0079
393	3079-62_2	TIG2	Q99969	RARRES2	1.78E-04	0.0424
394	3081-70_2	ULBP-1	Q9BZM6	ULBP1	9.63E-01	0.0005
395	3082-9_2	ULBP-2	Q9BZM5	ULBP2	7.93E-01	-0.0027
396	3083-71_1	XEDAR	Q9HAV5	EDA2R	1.43E-01	-0.0140
397	3091-70_2	Aurora kinas	O14965	AURKA	8.99E-01	0.0014
398	3115-64_2	MK01	P28482	MAPK1	8.38E-01	-0.0014
399	3122-6_2	SMAC	Q9NR28	DIABLO	3.93E-01	-0.0077
400	3132-1_1	VEGF-C	P49767	VEGFC	5.65E-01	-0.0055
401	3143-3_1	sCD4	P01730	CD4	7.48E-01	0.0034
402	3151-6_1	IL-2 sRa	P01589	IL2RA	7.42E-01	-0.0032
403	3152-57_1	TNF sR-II	P20333	TNFRSF1B	6.74E-01	0.0045
404	3166-92_1	Siglec-3	P20138	CD33	1.60E-01	-0.0156
405	3168-8_2	ADAMTS-5	Q9UNA0	ADAMTS5	8.46E-01	0.0019
406	3169-70_2	IDUA	P35475	IDUA	8.42E-01	-0.0023
407	3170-6_1	AMPM2	P50579	METAP2	7.44E-01	-0.0021
408	3171-57_2	amyloid prec	P05067	APP	4.81E-02	0.0210
409	3172-28_2	ARSB	P15848	ARSB	6.25E-01	-0.0046
410	3173-49_2	ASAHL	Q02083	NAAA	1.24E-01	-0.0127
411	3174-2_1	ATS1	Q9UHI8	ADAMTS1	4.15E-01	0.0065
412	3175-51_5	ATS13	Q76LX8	ADAMTS13	3.47E-01	0.0097
413	3177-49_2	Carbonic Anh	P22748	CA4	9.64E-03	-0.0237
414	3178-5_2	CATC	P53634	CTSC	5.39E-01	0.0051
415	3179-51_2	Cathepsin A	P10619	CTSA	9.00E-02	0.0192
416	3181-50_2	Cathepsin S	P25774	CTSS	3.05E-01	0.0118
417	3182-38_2	CD39	P49961	ENTPD1	4.69E-01	-0.0071
418	3184-25_2	Coagulation	P08709	F7	5.57E-01	0.0067
419	3186-2_2	C2	P06681	C2	1.26E-02	0.0284
420	3187-52_2	CRIS3	P54108	CRISP3	5.71E-02	0.0198
421	3189-61_2	Enterokinase	P98073	TMPRSS15	7.26E-01	0.0040
422	3191-50_2	WFKN1	Q96NZ8	WFIKKN1	1.08E-03	0.0317
423	3192-3_2	Glutamate c	Q96KP4	CNDP2	1.81E-01	0.0134
424	3194-36_2	GPVI	Q9HCN6	GP6	6.77E-01	-0.0044
425	3195-50_2	Granulysin	P22749	GNLY	5.49E-03	0.0317
426	3196-6_2	HPLN1	P10915	HAPLN1	6.35E-01	-0.0051
427	3197-70_2	IDE	P14735	IDE	2.70E-01	-0.0112
428	3198-4_1	IDS	P22304	IDS	4.27E-03	-0.0179
429	3199-54_2	kallikrein 12	Q9UKR0	KLK12	3.61E-04	-0.0342
430	3200-49_2	kallikrein 13	Q9UKR3	KLK13	1.02E-01	-0.0157
431	3201-49_2	kallikrein 5	Q9Y337	KLK5	5.48E-02	0.0188
432	3202-28_2	KREM2	Q8NCW0	KREMEN2	1.58E-03	-0.0170
433	3204-2_2	LKHA4	P09960	LTA4H	2.45E-01	0.0118
434	3206-4_2	LYVE1	Q9Y5Y7	LYVE1	2.97E-02	-0.0248
435	3208-2_4	MATN3	O15232	MATN3	7.48E-01	-0.0034
436	3209-69_1	MEPE	Q9NQ76	MEPE	8.58E-01	0.0016
437	3210-1_2	METAP1	P53582	METAP1	7.33E-02	-0.0143
438	3212-30_3	ASAH2	Q9NR71	ASAH2	1.86E-01	-0.0146
439	3213-65_2	Nidogen	P14543	NID1	4.42E-01	-0.0088
440	3216-2_2	PIGR	P01833	PIGR	5.24E-02	0.0223
441	3217-74_2	Protease nex	P07093	SERPINE2	9.53E-01	-0.0006
442	3220-40_2	RET	P07949	RET	5.83E-05	0.0426
443	3221-54_1	SARP-2	Q8N474	SFRP1	4.69E-02	0.0108
444	3222-11_2	Semaphorin	Q14563	SEMA3A	5.80E-01	0.0046
445	3232-28_2	TrATPase	P13686	ACP5	1.11E-01	-0.0164
446	3234-23_2	URB	Q76M96	CCDC80	2.22E-01	0.0132
447	3235-50_2	WFKN2	Q8TEU8	WFIKKN2	1.79E-06	-0.0443
448	3280-49_2	Aggrecan	P16112	ACAN	4.33E-06	-0.0410
449	3281-19_1	ANGL3	Q9Y5C1	ANGPTL3	5.30E-01	0.0054
450	3283-21_1	BGH3	Q15582	TGFBI	3.34E-02	-0.0214
451	3284-75_1	BGN	P21810	BGN	2.48E-01	-0.0088
452	3285-23_2	C1r	P00736	C1R	3.31E-01	0.0109
453	3289-19_2	Carbonic Anh	Q9NS85	CA10	3.10E-01	-0.0111
454	3290-50_2	CD109	Q6YHK3	CD109	1.39E-01	-0.0082
455	3291-30_2	CD23	P06734	FCER2	1.98E-01	0.0141
456	3292-75_1	CD48	P09326	CD48	5.98E-02	-0.0215
457	3293-2_4	CD5L	O43866	CD5L	3.89E-01	-0.0097
458	3294-55_2	CFC1	P0CG37	CFC1	4.94E-01	-0.0057
459	3296-92_2	CNTN2	Q02246	CNTN2	1.26E-02	-0.0266
460	3298-52_2	Contactin-4	Q8IWV2	CNTN4	1.30E-01	-0.0150
461	3299-29_2	Contactin-5	O94779	CNTN5	1.29E-01	-0.0149
462	3302-58_1	CYTF	O76096	CST7	7.49E-01	0.0034

463	3303-23_2	Cystatin M	Q15828	CST6	1.51E-01	-0.0158	9.01E-02	-0.0079	4.30E-01	-0.0040	5.73E-01	0.0036	9.00E-02	-0.3497	9.53E-02	0.1322	8.18E-01	0.0178	1.45E-01	-0.0770
464	3305-6_1	DLL4	Q9NR61	DLL4	8.50E-01	-0.0017	7.21E-01	-0.0014	5.52E-01	-0.0025	5.40E-01	-0.0032	5.85E-01	-0.0942	7.05E-01	-0.0251	2.95E-01	0.0674	6.87E-01	0.0178
465	3309-2_2	FCG2A	P12318	FCGR2A	8.00E-02	0.0208	8.13E-02	0.0087	6.46E-01	0.0025	9.09E-01	-0.0008	7.64E-01	-0.0671	5.40E-01	-0.0525	9.59E-01	0.0043	8.08E-01	0.0139
466	3310-62_1	FCG2B	P31994	FCGR2B	2.83E-01	0.0127	5.24E-01	0.0032	2.72E-01	0.0059	3.19E-01	0.0067	5.31E-01	0.1386	8.46E-01	0.0165	8.34E-01	-0.0173	7.22E-01	0.0202
467	3311-27_1	FCG3B	O75015	FCGR3B	3.64E-01	-0.0107	5.70E-01	-0.0028	2.37E-01	-0.0063	5.70E-01	-0.0038	6.01E-01	0.1156	4.54E-01	-0.0635	4.54E-01	-0.0617	5.71E-01	-0.0320
468	3312-64_1	FCGR1	P12314	FCGR1A	4.52E-01	0.0075	4.95E-01	0.0029	4.83E-01	-0.0032	2.62E-01	-0.0064	9.80E-01	0.0048	6.46E-02	0.1319	6.08E-01	-0.0357	2.42E-02	-0.1070
469	3313-21_2	FCN2	Q15485	FCN2	3.92E-02	0.0241	5.05E-02	0.0096	6.21E-01	-0.0026	5.57E-01	0.0039	1.57E-01	-0.3101	7.37E-01	-0.0283	4.69E-01	-0.0593	4.52E-01	-0.0422
470	3314-74_2	GFRa-1	P56159	GFRA1	1.97E-07	0.0474	5.45E-08	0.0208	4.47E-02	0.0086	6.87E-02	0.0098	4.78E-03	-0.4928	3.21E-01	0.0672	3.69E-03	0.1892	6.97E-01	-0.0176
471	3315-15_1	GPC2	Q8N158	GPC2	9.74E-01	0.0003	8.14E-01	0.0010	3.92E-01	-0.0039	2.64E-01	-0.0064	9.21E-01	-0.0188	5.58E-01	0.0426	4.03E-01	-0.0589	2.04E-01	-0.0614
472	3316-58_1	Heparin cofa	P05546	SERPIND1	9.85E-03	0.0302	4.53E-03	0.0140	4.15E-01	-0.0044	4.74E-01	-0.0048	5.10E-01	0.1456	5.54E-01	0.0502	3.62E-01	0.0750	6.01E-01	0.0296
473	3317-33_1	HTRA2	O43464	HTRA2	4.91E-01	-0.0034	1.50E-01	-0.0030	5.22E-01	0.0014	8.02E-02	0.0049	5.56E-02	0.1747	6.55E-01	0.0157	7.15E-01	0.0125	4.10E-01	-0.0193
474	3320-49_2	IGFBP-7	Q16270	IGFBP7	2.00E-01	0.0121	3.13E-01	0.0040	6.60E-01	0.0019	6.99E-01	-0.0021	5.63E-02	-0.3371	2.59E-01	-0.0769	5.89E-01	-0.0358	2.03E-01	0.0578
475	3321-2_2	IL24	Q13007	IL24	2.47E-01	-0.0126	1.95E-01	-0.0059	9.68E-01	0.0002	6.63E-01	-0.0027	7.44E-01	0.0664	7.07E-01	-0.0294	5.70E-01	0.0432	7.94E-01	-0.0136
476	3322-52_2	LRIG3	Q6UXM1	LRIG3	3.04E-01	0.0058	3.84E-01	0.0021	5.57E-01	0.0015	1.14E-01	0.0051	5.15E-01	0.0694	2.95E-01	0.0428	6.35E-02	0.0735	8.64E-01	-0.0047
477	3323-37_1	LRP8	Q14114	LRP8	9.25E-07	-0.0393	2.27E-08	-0.0187	5.43E-02	-0.0072	1.92E-01	-0.0061	1.12E-01	0.2448	6.68E-01	-0.0255	5.45E-01	-0.0349	5.08E-01	-0.0262
478	3324-51_1	LY9	Q9HBG7	LY9	8.55E-01	0.0006	7.06E-01	0.0005	2.36E-01	-0.0017	7.57E-01	0.0006	1.89E-01	-0.0781	2.99E-01	-0.0237	4.80E-01	-0.0157	8.33E-01	0.0032
479	3325-2_2	MATN2	O00339	MATN2	1.38E-01	-0.0161	3.70E-01	-0.0041	1.99E-03	-0.0152	1.99E-02	-0.0144	8.30E-01	0.0439	2.86E-01	0.0837	6.73E-01	-0.0322	7.30E-02	0.0934
480	3326-58_2	Nectin-like p	Q9BY67	CADM1	2.88E-02	-0.0106	2.95E-02	-0.0045	1.21E-01	-0.0034	6.32E-01	0.0013	4.52E-02	0.1827	9.54E-01	-0.0020	6.88E-01	0.0137	9.49E-01	0.0015
481	3327-27_1	NET4	Q9HB63	NTN4	5.21E-03	0.0325	2.70E-02	0.0109	7.79E-01	0.0015	9.65E-01	-0.0003	7.11E-02	-0.3949	2.79E-01	0.0912	1.45E-01	0.1193	5.64E-01	0.0324
482	3329-14_2	PGRP-S	O75594	PGLYRP1	4.89E-01	0.0078	2.42E-01	0.0056	8.48E-01	0.0010	7.31E-01	0.0022	3.94E-02	-0.4330	8.87E-01	0.0115	5.77E-01	-0.0440	2.85E-01	0.0578
483	3331-8_1	RGMB	Q6NW40	RGMB	2.82E-03	-0.0303	9.79E-03	-0.0111	2.65E-01	-0.0052	9.61E-01	0.0003	7.67E-01	-0.0570	4.47E-01	-0.0561	8.72E-01	0.0116	2.64E-01	-0.0548
484	3332-57_1	RGM-C	Q6ZVN8	HFE2	3.85E-01	0.0097	3.54E-01	0.0044	1.27E-01	0.0078	1.89E-01	0.0084	4.26E-01	0.1672	6.72E-01	0.0341	6.23E-04	0.2641	7.66E-01	-0.0160
485	3336-50_1	TFPI	P10646	TFPI	2.59E-06	0.0485	1.72E-05	0.0188	3.55E-03	0.0139	1.26E-01	0.0092	3.14E-02	-0.4249	3.19E-02	0.1628	2.36E-04	0.2678	4.29E-02	0.1024
486	3339-33_1	TSP2	P35442	THBS2	2.80E-02	0.0229	6.35E-02	0.0082	3.37E-02	0.0100	7.65E-03	0.0158	5.05E-01	-0.1307	9.38E-02	-0.1258	1.41E-03	0.2306	3.04E-02	0.1081
487	3340-53_1	TSP4	P35443	THBS4	1.56E-01	0.0165	6.54E-02	0.0090	7.82E-01	-0.0015	7.68E-02	-0.0117	8.09E-03	-0.5705	5.37E-01	0.0515	1.67E-01	0.1118	1.73E-02	-0.1316
488	3341-33_4	ABL1	P00519	ABL1	3.53E-01	0.0092	5.30E-01	0.0026	5.78E-01	-0.0025	4.50E-01	-0.0042	9.61E-01	0.0091	3.24E-01	-0.0699	3.52E-01	-0.0640	9.50E-01	-0.0030
489	3343-1_4	Aminoacylas	Q03154	ACY1	2.02E-01	0.0145	1.01E-01	0.0078	1.01E-01	0.0085	1.28E-01	0.0098	1.79E-01	-0.2860	9.89E-02	0.1347	2.14E-03	0.2412	5.31E-01	0.0342
490	3344-60_4	Antithrombi	P01008	SERPINC1	1.53E-11	-0.0713	2.09E-09	-0.0270	7.34E-01	-0.0017	9.40E-01	-0.0005	8.97E-02	0.3548	3.04E-01	-0.0827	9.38E-02	-0.1308	6.23E-01	-0.0264
491	3346-72_2	AURKB	Q96GD4	AURKB	7.57E-01	-0.0035	2.66E-01	-0.0053	9.38E-01	-0.0004	6.03E-01	0.0034	5.13E-01	-0.1388	6.98E-01	-0.0316	5.69E-01	-0.0451	7.60E-01	-0.0166
492	3347-9_2	BARK1	P25098	ADRBK1	8.61E-01	-0.0016	3.45E-01	-0.0036	1.94E-01	-0.0053	8.53E-01	0.0009	5.91E-01	0.0901	4.76E-01	0.0459	7.30E-01	-0.0216	9.63E-01	-0.0020
493	3348-49_2	BMP-1	P13497	BMP1	1.52E-01	0.0162	8.83E-02	0.0081	8.65E-01	0.0009	6.32E-01	-0.0031	9.29E-01	0.0189	5.73E-03	0.2228	5.45E-02	0.1514	6.45E-01	-0.0250
494	3350-53_2	CAMK2A	Q9UQM7	CAMK2A	3.79E-01	-0.0072	1.68E-01	-0.0047	6.93E-01	0.0015	9.06E-01	-0.0005	5.09E-01	0.1007	8.14E-01	-0.0138	1.60E-01	0.0798	5.17E-01	-0.0253
495	3351-1_1	CAMK2B	Q13554	CAMK2B	1.92E-02	-0.0163	1.38E-02	-0.0072	5.09E-01	-0.0021	6.35E-01	-0.0019	4.96E-02	0.2555	9.25E-01	0.0047	9.56E-01	-0.0027	2.06E-01	-0.0422
496	3352-80_3	Carbonic anh	P23280	CA6	4.30E-01	-0.0090	1.25E-01	-0.0073	4.10E-01	-0.0043	3.05E-01	-0.0067	1.16E-05	0.9133	2.55E-01	0.0932	2.15E-01	-0.0987	5.64E-02	-0.1038
497	3356-50_1	Carbonic anh	P43166	CA7	8.72E-01	-0.0017	6.96E-01	0.0017	6.41E-01	-0.0022	5.28E-01	-0.0038	4.52E-01	-0.1478	4.80E-01	0.0534	2.55E-02	-0.1630	6.57E-02	-0.0924
498	3357-67_2	CDK2/cyclin	P24941 P20	CDK2 CCNA2	7.09E-01	0.0041	9.61E-01	-0.0002	3.05E-01	0.0051	2.66E-01	0.0069	9.73E-01	0.0070	8.41E-01	-0.0157	5.80E-01	0.0421	7.15E-01	-0.0191
499	3358-51_2	CDK5/p35	Q00535 Q15	CDK5 CDK5R	9.89E-02	-0.0164	4.48E-02	-0.0084	1.31E-01	-0.0068	2.61E-01	-0.0064	7.23E-01	-0.0664	6.79E-01	-0.0297	6.85E-02	0.1267	7.65E-01	-0.0143
500	3359-11_2	CDK8/cyclin	P49336 P24	CDK8 CCNC	5.52E-02	-0.0209	3.98E-02	-0.0094	8.79E-03	-0.0129	9.69E-01	0.0002	4.80E-01	0.1446	5.99E-01	-0.0414	8.23E-01	-0.0171	7.17E-01	-0.0190
501	3360-50_4	Chk2	O96017	CHEK2	4.02E-01	0.0090	7.20E-01	-0.0016	8.71E-02	0.0083	1.29E-01	0.0092	2.73E-01	-0.2195	9.53E-01	-0.0046	7.96E-01	0.0193	4.42E-01	0.0395
502	3361-26_2	CLC4K	Q9UJ71	CD207	6.94E-01	-0.0043	2.52E-01	-0.0052	7.99E-01	-0.0012	3.55E-01	-0.0057	7.62E-01	-0.0614	8.31E-01	-0.0166	7.99E-01	-0.0192	7.63E-01	0.0156
503	3362-61_2	CRDL1	Q9BU40	CHRDL1	8.85E-03	-0.0286	2.42E-03	-0.0139	8.74E-02	-0.0085	9.04E-02	-0.0106	1.22E-01	0.3176	3.02E-01	-0.0816	4.62E-02	-0.1525	4.47E-01	-0.0401
504	3363-31_4	CSK	P41240	CSK	5.38E-01	-0.0039	3.65E-01	-0.0024	1.98E-03	-0.0088	3.02E-02	-0.0078	3.17E-02	0.2547	3.17E-01	0.0458	8.14E-02	-0.0773	3.34E-01	-0.0294
505	3364-76_2	Cathepsin V	O60911	CTSV	6.16E-02	-0.0149	1.54E-01	-0.0048	6.23E-01	-0.0018	7.22E-01	0.0016	9.15E-04	0.4907	6.21E-01	0.0285	8.96E-01	0.0073	7.29E-01	-0.0133
506	3365-7_2	Dkk-4	Q9UBT3	DKK4	3.45E-01	0.0107	6.94E-01	0.0019	2.99E-01	0.0053	5.78E-01	0.0036	7.22E-01	-0.0753	1.44E-01	0.1183	6.67E-02	0.1441	1.01E-01	0.0886
507	3366-51_2	ECM1	Q16610	ECM1	1.88E-01	-0.0156	1.73E-01	-0.0068	6.71E-03	-0.0145	2.78E-02	-0.0148	5.03E-01	0.1485	9.60E-01	0.0043	5.99E-02	-0.1549	1.65E-01	-0.0786
508	3367-8_3	FETUB	Q9UGM5	FETUB	9.08E-01	0.0012	8.18E-01	0.0010	1.72E-01	-0.0065	4.20E-01	-0.0048	4.44E-01	0.1494	8.99E-01	0.0095	1.15E-01	0.1146	3.27E-01	-0.0490
509	3373-5_2	Granzyme H	P20718	GZMH	1.42E-01	0.0160	1.71E-01	0.0063	9.62E-01	-0.0002	4.32E-01	-0.0049	5.29E-01	-0.1287	3.44E-01	0.0743	7.33E-01	-0.0260	6.53E-01	-0.0235
510	3374-49_2	HCK	P08631	HCK	3.11E-01	-0.0093	4.64E-01	-0.0028	6.88E-01	-0.0017	8.07E-01	-0.0013	2.62E-01	-0.1920	2.26E-01	-0.0796	7.78E-02	0.1124	4.32E-01	-0.0345
511	3376-49_2	IL-17 RD	Q8NFM7	IL17RD	3.53E-01	-0.0100	7.08E-01	-0.0017	2.48E-02	-0.0109	5.22E-01	-0.0039	1.74E-01	-0.2728	6.48E-01	-0.0353	7.32E-01	0.0257	6.89E-01	-0.0206
512	3378-49_2	Kallikrein 7	P49862	KLK7	7.46E-01	-0.0038	8.29E-01	-0.0011	3.10E-01	-0.0054	4.68E-01	-0.0048	5.92E-01	-0.1170	9.81E-01	-0.0020	5.62E-01	0.0473	4.72E-02	-0.1104
513	3379-29_1	KPCI	P41743	PRKCI	5.82E-02	-0.0102	1.29E-01	-0.0034	1.17E-01	0.0038	3.67E-01	0.0028	4.29E-03	-0.2861	3.15E-01	-0.0391	1.06E-01	0.0608	6.33E-02	-0.0479
514	3381-24_2	LYNB	P07948	LYN	3.35E-02	-0.0152	4.96E-02	-0.0059	3.83E-03	-0.0093	1.40E-02	-0.0100	2.86E-01	0.1433	2.50E-01	0.0594	6.59E-02	-0.0919	1.91E-01	-0.0450
515	3387-1_2	PAK3	O75914	PAK3	2.48E-05	-0.0438	2.87E-05	-0.0183	1.06E-03	-0.0156	1.22E-02	-0.0151	8.39E-01	-0.0405	2.61E-01	-0.0858	1.20E-01	0.1150	3.83E-01	-0.0444
516	3388-58_2	PAK7	Q9P286	PAK7	6.23E-01	-0.0049	3.72E-01	-0.0038	1.62E-01	-0.0063	5.25E-01	-0.0036	1.65E-01	0.2591	6.63E-01	0.0313	4.41E-01	-0.0537	6.24E-01	-0.0235
517	3389-7_2	PCI	P05154	SERPINA5	1.91E-02	-0.0255	3.88E-02	-0.0095	1.64E-02	0.0118	1.94E-02	0.0145	1.78E-01	0.2749	7.39E-01	-0.0262	5.74E-01	0.0429	3.23E-01	0.0517
518	3390-72_2	PIK3CA/PIK3	P42336 P27	PIK3CA PIK3	4.74E-03	0.0253	1.80E-01	0.0051	5.25E-01	0.0026	5.06E-01	0.0034	7.67E-01	-0.0500	8.65E-01	-0.0110	2.15E-02	0.1441	7.56E-01	0.0135
519	3391-10_2	PK3CG	P48736	PIK3CG	2.72E-01	-0.0111	1.16E-01	-0.0067	5.69E-01	-0.0026	3.89E-01	-0.0050	6.78E-01	0.0790	5.37E-02	-0.1403	8.47E-01	0.0137	3.99E-01	0.0410
520	3392-68_2	PKB a/b/g	P31749 P31	AKT1 AKT2 A	1.00E+00	0.0000	4.17E-01	-0.0030	4.70E-02	-0.0080	1.07E-02	-0.0128	9.39E-02	0.2776	1.81E-01	0.0852	2.61E-01	-0.0697	4.22E-01	-0.0341
521	3394-81_2	PLK-1	P53350	PLK1	8.27E-01	-0.0024	4.31E-01	-0.0036	7.74E-01	0.0014	8.77E-01	0.0010	5.01E-01	0.1379	8.38E-01	-0.0161	6.94E-01	-0.0301	3.13E-01	-0.0530
522	3396-54_2	Renin	P00797	REN	2.70E-02	-0.0251	8.96E-02	-0.0081	1.06E-02	-0.0132	3.86E-02	-0.0134	1.05E-01	0.3455	3.18E-01	-0.0820	2.43E-01	-0.0930	3.99E-02	0.1119
523	3397-7_4	SHP-2	Q06124	PTPN11	6.71E-01	-0.0041	9.09E-01	-0.0005	4.62E-01	0.0032	5.71E-01	-0.0031	9.54E-01	-0.0104	6.67E-01	0.0300	4.98E-01	0.0458	3.78E-01	-0.0409
524	3399-31_2	STAB2	Q8WWQ8	STAB2	3.67E-02	-0.0225	8.04E-03	-0.0120	9.81E-02	-0.0081	5.05E-01	-0.0041	1.42E-01	0.2976	3.19E-02	-0.1665	2.78E-02	-0.1657	7.70E-01	0.0152
525	3400-49_2	TBK1	Q9UHD2	TBK1	8.87E-01	0.0013	8.34E-01	-0.0008	8.77E-01	-0.0006	3.34E-01	-0.0050	6.47E-01	-0.0780	1.21E-01	-0.1012	2.20E-01	0.0778	7.04E-01	0.0166
526	3401-8_2	TCPTP	P17706	PTPN2	5.93E-01	0.0056	7.25E-01	-0.0015	7.21E-01	0.0017	9.12E-01	0.0007	7.71E-01	-0.0569	9.83E-01	0.0016	5.02E-01	-0.0489	3.07E-01	0.0511
527	3403-1_2	TPSB2	P20231	TPSB2	6.83E-01	0.0048	6.62E-01	0.0022	5.00E-02	-0.0105	8.14E-01	-0.0016	1.31E-01	-0.3340	8.53E-01	0.0158	6.74E-01	-0.0348	1.25E-01	0.0869
528	3404-51_2	TPSG1	Q9NRR2	TPSG1	9.76E-01	0.0003	5.33E-01	0.0024	3.87E-01	-0.0036	4.14E-01	-0.0042	6.35E-02	-0.3134	8.04E-01	-0.0162	6.21E-01	0.0313	6.06E-01	0.0224
529	3405-6_2	UFC1	Q9Y3C8	UFC1	4.10E-01	-0.0054	2.58E-01	-0.0031	1.91E-01	0.0039	5.15E-01	0.0024	9.94E-02	-0.2001	8.54E-01	-0.0086	8.41E-01	0.0091	6.97E-01	-0.0122
530	3412-7_1	Bcl-2	P10415	BCL2	5.52E-03	-0.0157	4.39E-03	-0.0068	3.49E-01	-0.0024	7.89E-01	0.0009	7.85E-01	0.0290	1.61E-01	-0.0573	4.43E-01	0.0306	4.04E-01	-0.0228
531	3413-50_2	BFL1	Q16548	BCL2A1	5.25E-01	-0.0063	5.37E-01	-0.0026	2.92E-01	0.0047	9.91E-01	0.0001	6.11E-01	0.0942	8.92E-01	-0.0097	3.50E-01	0.0645	7.78E-01	-0.0134
532	3414-40_2	BMX	P51813	BMX	2.48E-01	0.0104	1.94E-01	0.0049	2.27E-02	0.0093	8.71E-01	0.0008	1.20E-01	-0.2619	5.62E-02	0.1234	8.05E-01	0.0156	7.97E-02	-0.0755
533	3415-61_2	BSP	P21815	IBSP	5.32E-04	-0.0359	4.18E-04	-0.0154	9.04E-01	-0.0006	7.22E-01	0.0021	1.22E-02	0.4889	1.50E-02	-0.1824	2.14E-01	-0.0911	1.06E-01	-0.0812
534	3416-2_2	BTK	Q06187	BTK	1.13E-01	-0.0113	3.52E-02	-0.0063	2.64E-04	-0.0117	2.49E-02	-0.0091	3.15E-02	0.2869	1.44E-01	0.0752	1.58E-01	-0.0706	7.69E-01	-0.0101
535	3418-12_2	CAMK1D	Q8IU85	CAMK1D	7.25E-02	-0.0170	3.14E-02	-0.0086	9.99E-01	0.0000	2.63E-01	-0.0060	1.61E-01	-0.2482	2.80E-01	0.0736	2.23E-01	0.0807	8.92E-01	-0.0062
536	3419-49_2	CAMK2D	Q13557	CAMK2D	7.69E-02	-0.0116	1.36E-01	-0.0041	2.25E-01	-0.0036	6.01E-01	-0.0020	5.45E-02	0.2352	8.35E-01	-0.0098	6.05E-01	-0.0237	5.30E-01	-0.0197
537	3420-21_2	Carbonic anh	Q8N1Q1	CA13	3.78E-02	-0.0177	1.36E-03	-0.0114	6.35E-02	-0.0072	2.92E-02	-0.0106	3.29E-01	0.1561	7.59E-01	0.0188	8.28E-01	0.0129	2.71E-01	-0.0451
538	3421-54_2	CD30 Ligand	P32971	TNFSF8	6.73E-03	-0.0184	2.64E-03	-0.0086	9.72E-01	0.0001	8.15E-01	0.0009	2.97E-01	-0.1339	1.21E-01	-0.0763	7.16E-01	-0.0174	4.33E-01	-0.0258
539	3422-4_2	CDK1/cyclin	P06493 P14	CDC2 CCNB1	1.69E-01	-0.0117	7.92E-02	-0.0063	7.11E-02	-0.0070	7.19E-01	0.0018	9.13E-01	0.0176	8.39E-01	-0.0125	7.39E-01	0.0199	8.14E-01	0.0096
540	3423-59_2	Chymase	P23946	CMA1	1.06E-01	-0.0113	2.91E-01	-0.0031	2.66E-01	-0.0035	4.06E-01	0.0033	3.78E-01	0.1153	4.53E-01	-0.0378	2.30E-01	-0.0586	3.02E-01	0.0346
541	3427-63_2	CSK21	P68400	CSNK2A1	8.51E-01	-0.0016	4.95E-01	-0.0025	8.46E-01	0.0008	2.13E-01	0.0061	3.85E-01	-0.1392	2.71E-01	-0.0678	5.67E-01	0.0343	4.92E-01	-0.0282
542	3431-54_2	EphA1	P21709	EPHA1	1.31E-01	-0.0070	1.80E-01	-0.0026	4.23E-01	0.0017	1.54E-01	0.0038	7.63E-01	-0.0263	2.07E-02	-0.0770	1.46E-01	0.0473	2.01E-01	-0.0285
543	3432-21_2	EPHA3	P29320	EPHA3	3.44E-01	0.0084	8.68E-01	0.0006	3.19E-01	0.0040	6.05E-01	-0.0026	7.51E-01	-0.0529	4.64E-01	-0.0468	3.07E-01	0.0634	8.38E-01	-0.0087
544	3434-34_1	FN1.3	P02751	FN1	6.34E-02	0.0207	1.53E-01	0.0067	7.62E-01	-0.0015	9.82E-02	-0.0105	6.73E-01	0.0882	1.29E-01	0.1216	9.03E-01	0.0095	8.58E-01	0.0096
545	3435-53_2	FN1.4	P02751	FN1	8.11E-02	0.0158	9.09E-02	0.0065	7.08E-01	-0.0016	6.34E-02	-0.0096	6.20E-01	0.0844	2.60E-01	0.0736	6.56E-01	0.0283	3.94E-01	-0.0372
546	3437-80_3	Flt-3	P36888	FLT3	3.88E-03	-0.0214	4.90E-04	-0.0108	2.47E-03	-0.0102	7.86E-01	-0.0012	4.30E-02	0.2817	9.66E-02	-0.0891	4.13E-02	-0.1060	6.42E-01	0.0167
547	3438-10_2	FSTL3	O95633	FSTL3	7.24E-03	0.0255	2.09E-03	0.0123	4.77E-01	0.0031	5.24E-02	0.0106	4.05E-01	0.1494	8.98E-01	-0.0089	7.73E-01	-0.0193	6.41E-01	0.0214
548	3440-7_2	granzyme A	P12544	GZMA	9.78E-02	0.0183	1.61E-01	0.0065	7.92E-01	0.0013	7.40E-01	0.0021	2.35E-02	-0.4661	2.34E-01	-0.0944	7.04E-01	0.0293	7.06E-01	-0.0200
549	3441-64_2	GSK-3 alpha	P49840 P49	GSK3A GSK3	5.55E-03	-0.0167	4.60E-03	-0.0072	4.47E-01	-0.0021	7.80E-01	0.0010	1.39E-01	0.1679	9.78E-01	0.0012	6.85E-01	-0.0172	2.93E-01	-0.0306
550	3443-61_2	HIPK3	Q9H422	HIPK3	7.20E-01	0.0037	3.22E-01	-0.0043	5.03E-01	0.0032	5.38E-01	0.0036	8.70E-01	-0.0317	8.78E-01	-0.0115	8.92E-01	0.0098	9.57E-01	-0.0027
551	3445-53_2	IL-15 Ra	Q13261	IL15RA	1.67E-01	-0.0145	4.95E-02	-0.0087	7.40E-01	0.0016	2.86E-01	0.0064	3.82E-01	0.1721	6.89E-01	-0.0303	2.87E-01	-0.0781	6.20E-01	0.0250
552	3446-7_2	IL-18 Ra	Q13478	IL18R1	1.90E-01	0.0141	3.91E-01	0.0039	9.65E-02	0.0081	1.08E-02	0.0155	9.54E-01	-0.0116	3.09E-01	-0.0786	1.58E-01	0.1059	4.55E-02	0.1026
553	3447-64_2	IL-8	P10145	CXCL8	8.69E-01	0.0017	7.88E-01	-0.0012	2.72E-01	0.0051	2.82E-01	0.0062	3.04E-01	0.1960	3.21E-01	0.0727	9.11E-01	0.0080	2.00E-01	0.0626
554	3448-13_2	IR	P06213	INSR	3.77E-01	0.0063	5.20E-01	0.0019	2.64E-01	0.0036	3.38E-01	0.0039	3.73E-01	-0.1181	2.70E-01	0.0561	3.86E-01	0.0429	7.34E-01	-0.0115
555	3449-58_2	Kallistatin	P29622	SERPINA4	7.00E-03	-0.0308	4.51E-03	-0.0137	1.72E-01	0.0071	4.22E-01	0.0053	2.86E-02	0.4695	8.58E-01	0.0149	6.13E-01	0.0407	9.86E-01	0.0010
556	3450-4_2	Kallikrein 6	Q92876	KLK6	6.98E-01	-0.0036	6.26E-01	0.0019	1.98E-01	-0.0054	2.40E-01	-0.0062	4.42E-02	-0.3450	8.13E-01	-0.0157	1.29E-02	0.1587	2.42E-01	0.0516
557	3452-17_2	LCK	P06239	LCK	7.26E-01	-0.0038	2.95E-01	-0.0048	8.06E-01	0.0012	6.89E-01	-0.0025	7.25E-01	0.0724	1.80E-01	-0.1057	6.27E-01	0.0373	7.01E-01	0.0203
558	3453-87_2	LYN	P07948	LYN	1.04E-01	-0.0123	9.71E-02	-0.0053	6.69E-03	-0.0092	3.51E-02	-0.0090	1.36E-01	0.2101	5.76E-01	0.0303	1.72E-01	-0.0718	2.44E-01	-0.0421
559	3457-57_1	Periostin	Q15063	POSTN	1.78E-02	-0.0223	1.65E-03	-0.0124	2.71E-01	-0.0047	1.85E-01	-0.0071	8.06E-01	-0.0437	3.20E-01	0.0677	8.84E-01	0.0096	1.22E-01	-0.0701
560	3459-49_2	PDGF Rb	P09619	PDGFRB	1.99E-01	-0.0108	1.55E-01	-0.0051	6.30E-01	0.0019	9.10E-01	0.0005	9.98E-01	0.0004	8.37E-01	-0.0125	3.30E-01	0.0576	7.41E-01	-0.0134
561	3461-58_1	PGCB	Q96GW7	BCAN	3.02E-07	-0.0540	3.55E-09	-0.0259	2.43E-01	-0.0058	4.30E-01	-0.0049	6.93E-02	0.3692	9.60E-01	-0.0039	6.32E-01	-0.0365	3.22E-01	-0.0518
562	3466-8_2	PRKACA	P17612	PRKACA	4.72E-01	0.0053	9.95E-01	0.0000	4.32E-01	-0.0026	7.77E-01	-0.0012	5.20E-01	0.0883	9.30E-01	-0.0046	7.84E-01	0.0140	2.45E-01	-0.0407
563	3469-74_1	RPS6KA3	P51812	RPS6KA3	3.93E-01	-0.0062	6.10E-01	-0.0016	7.72E-02	-0.0058	3.56E-01	-0.0038	8.25E-01	-0.0299	6.80E-01	0.0215	6.14E-01	-0.0255	2.36E-01	-0.0410
564	3470-1_2	sE-Selectin	P16581	SELE	3.20E-02	0.0249	3.58E-02	0.0103	8.62E-01	-0.0009	3.07E-01	0.0068	9.11E-03	-0.5652	1.62E-01	0.1173	9.86E-02	0.1343	5.56E-01	0.0329
565	3471-49_1	STK16	O75716	STK16	3.94E-01	-0.0066	5.37E-02	-0.0063	5.67E-01	0.0020	8.69E-01	0.0007	4.67E-01	-0.1054	7.47E-02	-0.0989	5.55E-02	0.1032	4.38E-01	0.0288
566	3472-40_2	Survivin	O15392	BIRC5	8.08E-01	0.0026	8.97E-01	0.0006	3.90E-01	-0.0042	7.20E-01	0.0022	3.02E-01	0.2055	9.02E-01	-0.0095	6.33E-01	-0.0355	6.38E-01	0.0240
567	3473-78_2	Thrombopoi	P40238	MPL	6.24E-02	0.0199	2.39E-01	0.0053	1.92E-01	0.0063	3.12E-01	0.0062	9.18E-01	-0.0206	2.45E-01	0.0894	3.08E-03	0.2192	3.27E-01	0.0503
568	3474-19_2	Thrombospo	P07996	THBS1	1.50E-02	0.0242	3.01E-02	0.0091	6.78E-02	0.0083	3.99E-01	0.0048	8.64E-01	0.0320	7.50E-02	0.1276	3.45E-02	0.1470	4.41E-02	0.0961
569	3477-63_2	TrkA	P04629	NTRK1	5.00E-01	0.0054	9.96E-01	0.0000	5.21E-01	-0.0023	1.33E-01	-0.0068	7.96E-01	-0.0385	9.99E-01	0.0001	8.28E-01	-0.0121	9.30E-01	-0.0034
570	3479-71_2	TRY3	P35030	PRSS3	6.00E-01	-0.0056	6.11E-01	-0.0023	2.51E-01	0.0055	1.80E-01	0.0081	7.57E-01	0.0615	4.95E-01	-0.0519	4.14E-02	0.1502	2.15E-01	0.0628
571	3480-7_1	DUS3	P51452	DUSP3	4.50E-01	0.0069	9.15E-01	-0.0004	4.18E-02	-0.0084	3.65E-02	-0.0108	2.12E-01	0.2128	3.44E-01	0.0620	8.53E-01	0.0118	5.95E-02	-0.0821
572	3481-87_1	XPNPEP1	Q9NQW7	XPNPEP1	2.52E-01	0.0062	4.19E-01	0.0018	6.05E-01	0.0013	1.94E-01	0.0040	3.57E-01	0.0935	6.28E-01	0.0189	3.86E-01	0.0328	7.73E-01	-0.0075
573	3484-60_2	Angiotensin	P01019	AGT	3.23E-01	-0.0110	2.94E-01	-0.0049	4.88E-01	0.0035	3.78E-02	0.0131	2.16E-03	0.6315	6.55E-01	0.0357	5.91E-01	-0.0418	2.95E-01	0.0558
574	3485-28_2	b2-Microglol	P61769	B2M	4.19E-01	-0.0088	8.35E-01	-0.0010	2.64E-01	0.0055	1.80E-01	0.0083	4.79E-02	-0.4004	3.03E-03	-0.2291	2.48E-01	-0.0876	1.39E-01	-0.0769
575	3486-58_2	b-ECGF	P05230	FGF1	1.54E-03	0.0313	4.31E-03	0.0119	5.69E-02	0.0086	4.04E-01	0.0048	2.67E-02	-0.4127	7.18E-01	0.0260	4.96E-02	0.1367	7.05E-01	0.0182
576	3487-32_2	BLC	O43927	CXCL13	6.86E-01	-0.0045	4.17E-01	-0.0038	1.62E-01	-0.0071	4.08E-02	-0.0130	9.80E-01	-0.0053	6.57E-01	0.0357	7.10E-01	0.0290	1.41E-01	0.0787
577	3488-64_2	Catalase	P04040	CAT	3.57E-01	-0.0062	7.53E-01	-0.0009	1.03E-04	0.0116	7.73E-04	0.0127	1.76E-01	-0.1699	4.40E-01	-0.0374	2.33E-02	0.1059	2.16E-01	-0.0398
578	3489-9_2	CNTF	P26441	CNTF	4.46E-01	0.0072	3.96E-01	0.0034	4.43E-01	0.0033	9.91E-01	-0.0001	7.77E-02	-0.3123	1.81E-01	0.0912	3.93E-01	0.0566	6.50E-01	0.0206
579	3494-71_3	FGF-17	O60258	FGF17	5.47E-01	0.0067	5.94E-01	0.0025	9.39E-01	-0.0004	8.76E-01	-0.0010	9.54E-02	-0.3445	2.16E-01	0.0984	2.39E-01	0.0909	8.91E-01	0.0073
580	3495-15_2	GCP-2	P80162	CXCL6	6.89E-01	0.0045	7.70E-01	0.0014	1.09E-01	0.0081	9.51E-02	0.0106	3.25E-01	0.2062	7.37E-01	-0.0271	9.08E-01	-0.0091	1.90E-04	0.1971
581	3497-13_2	IFN-aA	P01563	IFNA2	8.45E-01	-0.0022	4.11E-01	-0.0038	5.50E-01	0.0030	9.72E-01	0.0002	3.22E-01	0.2054	6.60E-01	0.0351	4.45E-01	0.0591	8.48E-01	0.0102
582	3499-77_2	IL-17B	Q9UHF5	IL17B	4.49E-01	0.0080	7.23E-01	0.0016	5.10E-01	0.0032	5.09E-01	-0.0040	3.50E-02	-0.4145	9.24E-01	0.0073	1.87E-02	0.1722	8.02E-01	-0.0127
583	3503-4_2	Integrin a1b	P56199, P05	ITGA1 ITGB1	4.62E-01	0.0079	9.98E-01	0.0000	3.30E-01	0.0048	7.73E-01	0.0018	8.10E-01	0.0484	3.02E-02	0.1666	2.25E-01	0.0910	1.23E-01	-0.0794
584	3504-58_2	LEAP-1	P81172	HAMP	3.06E-01	0.0101	2.83E-01	0.0045	5.76E-02	0.0085	1.07E-01	0.0091	1.82E-02	-0.4332	8.53E-01	0.0131	3.56E-01	0.0636	1.19E-02	-0.1181
585	3505-6_2	Lymphotoxin	P01374, Q06	LTA LTB	8.70E-01	0.0011	6.38E-01	-0.0013	8.84E-01	-0.0004	5.08E-01	-0.0025	5.90E-02	-0.2369	2.63E-01	-0.0541	2.59E-01	0.0531	2.23E-01	-0.0392
586	3506-49_1	Lymphotoxin	P01374, Q06	LTA LTB	9.52E-03	-0.0259	2.93E-03	-0.0125	1.39E-02	-0.0112	1.35E-02	-0.0141	5.25E-02	-0.3645	3.02E-01	-0.0748	8.22E-01	0.0159	1.32E-02	-0.1189
587	3508-78_3	MDC	O00626	CCL22	3.25E-01	0.0115	4.26E-01	0.0039	9.55E-01	-0.0003	2.02E-01	-0.0085	7.24E-02	-0.3920	1.18E-01	0.1313	6.92E-01	0.0324	2.83E-01	-0.0602
588	3509-1_1	MIP-5	Q16663	CCL15	5.30E-02	-0.0219	1.34E-01	-0.0072	1.97E-01	0.0067	6.65E-02	0.0119	4.66E-01	-0.1550	9.77E-01	0.0023	2.08E-01	0.0998	3.09E-01	0.0554
589	3514-49_2	Proteinase-3	P24158	PRTN3	4.35E-01	0.0086	1.76E-01	0.0062	5.23E-01	0.0032	1.20E-02	0.0156	4.98E-01	-0.1391	5.42E-02	-0.1511	9.43E-02	-0.1277	5.54E-01	-0.0311
590	3516-60_2	SDF-1	P48061	CXCL12	1.85E-01	-0.0151	3.91E-02	-0.0099	7.31E-01	0.0018	6.92E-01	-0.0026	4.18E-01	0.1729	1.02E-01	-0.1337	1.87E-01	-0.1051	2.91E-01	-0.0577
591	3518-54_2	TAFI	Q96IY4	CPB2	1.85E-01	0.0155	1.98E-02	0.0114	4.77E-01	-0.0038	6.35E-01	0.0032	8.62E-01	-0.0379	1.17E-01	0.1312	8.63E-01	-0.0141	5.18E-01	0.0362
592	3519-3_2	TARC	Q92583	CCL17	6.99E-01	0.0041	9.59E-01	0.0002	8.99E-01	0.0006	8.56E-01	0.0011	9.99E-01	-0.0002	2.61E-02	0.1686	1.96E-01	0.0956	3.92E-01	0.0435
593	3520-58_1	TGF-b3	P10600	TGFB3	2.55E-01	0.0130	1.47E-01	0.0070	7.39E-01	0.0017	8.56E-01	0.0012	3.91E-01	-0.1832	5.99E-01	0.0432	3.53E-01	0.0740	1.46E-01	0.0793
594	3521-16_2	TSH	P01215 P01	CGA TSHB	3.70E-02	0.0209	8.98E-02	0.0072	9.27E-01	-0.0004	9.60E-01	-0.0003	3.84E-01	-0.1643	1.97E-02	0.1680	1.44E-01	0.1028	6.70E-01	0.0206
595	3522-57_1	Vasoactive I	P01282	VIP	1.92E-01	-0.0086	8.61E-01	-0.0005	6.16E-01	0.0015	1.64E-01	0.0052	3.02E-01	-0.1268	6.46E-01	0.0217	8.26E-01	0.0101	3.28E-01	-0.0308
596	3534-14_2	CD40 ligand,	P29965	CD40LG	8.33E-01	0.0021	4.59E-01	-0.0031	9.11E-01	0.0005	6.10E-01	0.0029	9.22E-02	-0.3138	9.13E-01	0.0078	1.93E-01	0.0907	3.99E-01	0.0404
597	3535-84_1	DKK1	O94907	DKK1	1.92E-01	0.0145	1.99E-01	0.0060	7.90E-02	0.0088	2.84E-01	0.0068	8.16E-01	-0.0484	9.47E-02	0.1331	3.25E-02	0.1649	6.95E-02	0.0962
598	3538-26_1	dopa decarb	P20711	DDC	3.09E-01	-0.0116	3.97E-01	-0.0041	6.55E-01	0.0023	7.92E-01	0.0017	4.32E-01	-0.1675	7.55E-01	0.0256	2.33E-01	0.0949	1.48E-01	-0.0788
599	3554-24_1	Adiponectin	Q15848	ADIPOQ	1.45E-06	-0.0532	7.22E-06	-0.0209	9.21E-03	-0.0133	1.32E-02	-0.0160	6.32E-09	1.1837	1.97E-01	-0.1053	9.65E-06	-0.3427	3.32E-01	-0.0528
600	3580-25_8	a1-Antitryps	P01009	SERPINA1	3.65E-01	-0.0069	6.39E-02	-0.0059	3.00E-01	0.0036	7.51E-01	0.0014	3.71E-02	0.2956	6.98E-01	0.0213	3.29E-01	-0.0519	9.53E-01	-0.0021
601	3581-53_3	a2-HS-Glyco	P02765	AHSG	1.16E-01	-0.0182	1.58E-02	-0.0117	4.92E-02	-0.0103	4.14E-02	-0.0134	9.34E-01	0.0179	8.02E-01	0.0210	5.71E-01	0.0459	6.59E-01	-0.0245
602	3583-54_4	Arylsulfatas	P15289	ARSA	8.40E-02	0.0170	2.09E-01	0.0052	9.13E-03	0.0116	1.58E-02	0.0135	2.39E-01	0.2167	6.98E-01	0.0275	1.14E-02	0.1729	1.02E-01	0.0769
603	3585-54_4	BASI	P35613	BSG	4.09E-01	-0.0078	1.23E-01	-0.0062	8.74E-01	0.0007	8.74E-01	-0.0009	1.67E-01	0.2450	9.27E-01	0.0063	7.97E-01	-0.0171	9.43E-01	0.0033
604	3587-53_2	BMP10	O95393	BMP10	4.99E-01	-0.0068	2.42E-01	-0.0050	1.13E-01	-0.0073	1.03E-01	-0.0094	4.98E-01	0.1285	7.99E-01	-0.0185	7.26E-01	0.0248	3.10E-01	-0.0493
605	3591-51_3	Cadherin-6	P55285	CDH6	7.19E-01	0.0041	8.07E-01	0.0012	8.95E-01	0.0007	4.43E-01	-0.0049	5.57E-01	-0.1238	3.82E-01	0.0708	2.44E-01	0.0916	9.71E-01	0.0020
606	3592-4_3	CAMK1	Q14012	CAMK1	7.47E-01	0.0037	4.24E-01	0.0039	1.64E-01	-0.0073	9.90E-01	-0.0001	9.28E-01	0.0195	2.30E-01	0.0997	4.65E-01	-0.0590	9.64E-01	0.0025
607	3593-72_3	Caspase-3	P42574	CASP3	2.82E-01	-0.0073	2.45E-01	-0.0033	2.10E-01	-0.0039	1.15E-01	-0.0061	7.27E-02	0.2268	1.18E-01	0.0759	1.73E-01	-0.0644	1.38E-01	-0.0480
608	3594-6_5	CATE	P14091	CTSE	5.53E-01	-0.0058	3.32E-01	-0.0040	6.66E-01	-0.0019	3.09E-01	-0.0056	6.93E-01	0.0716	8.17E-01	-0.0161	8.00E-01	-0.0171	1.62E-01	-0.0649
609	3600-2_3	Chitotriosida	Q13231	CHIT1	2.96E-01	-0.0121	7.59E-02	-0.0086	5.58E-01	0.0031	4.95E-01	0.0045	4.44E-02	0.4335	4.20E-01	-0.0672	3.96E-02	-0.1656	6.12E-01	0.0281
610	3601-54_3	CHL1	O00533	CHL1	2.29E-02	-0.0235	1.38E-02	-0.0107	1.19E-01	-0.0074	1.42E-01	-0.0087	3.97E-04	0.6780	2.37E-01	0.0882	6.93E-01	-0.0286	4.21E-01	-0.0401
611	3603-60_5	CLC7A	Q9BXN2	CLEC7A	7.70E-01	0.0023	6.99E-01	-0.0013	6.35E-01	0.0017	5.66E-01	-0.0025	3.98E-01	0.1221	9.63E-01	-0.0026	2.68E-01	0.0597	4.36E-01	0.0288
612	3605-77_4	MASP3	P48740	MASP1	7.42E-01	-0.0033	4.68E-01	-0.0030	8.10E-02	-0.0078	2.45E-01	-0.0066	4.33E-01	0.1452	9.89E-01	-0.0010	8.29E-01	-0.0149	8.64E-01	0.0081
613	3606-2_1	Discoidin do	Q16832	DDR2	1.19E-01	-0.0146	7.08E-02	-0.0071	4.55E-01	-0.0032	5.68E-01	-0.0031	7.86E-01	0.0479	2.60E-01	0.0762	1.25E-01	-0.1006	8.85E-01	0.0065
614	3607-71_1	DKK3	Q9UBP4	DKK3	1.09E-03	-0.0345	1.40E-03	-0.0142	5.99E-01	-0.0026	6.19E-01	-0.0030	4.56E-02	0.3984	6.82E-01	-0.0315	4.34E-01	0.0585	6.29E-02	-0.0950
615	3608-12_1	DPP2	Q9UHL4	DPP7	3.11E-01	-0.0104	1.26E-01	-0.0066	6.72E-01	-0.0020	8.27E-01	0.0013	4.73E-01	0.1374	4.92E-01	0.0505	9.25E-01	-0.0067	4.51E-01	-0.0370
616	3611-70_4	Endothelin-c	P42892	ECE1	5.01E-01	0.0074	9.09E-01	-0.0005	1.45E-01	-0.0073	7.71E-02	-0.0111	5.60E-02	-0.3925	3.44E-01	0.0749	8.12E-01	0.0183	4.20E-02	-0.1069
617	3612-6_4	EphB4	P54760	EPHB4	6.71E-01	-0.0028	3.17E-01	-0.0028	8.81E-01	0.0005	6.35E-01	0.0018	1.41E-01	0.1818	8.69E-01	0.0078	3.41E-01	0.0440	3.11E-03	0.0928
618	3613-62_1	FCN1	O00602	FCN1	7.31E-01	-0.0031	3.93E-01	-0.0033	3.45E-01	-0.0039	6.26E-01	-0.0025	8.83E-02	0.2899	4.02E-01	-0.0549	1.70E-01	-0.0872	6.92E-01	0.0173
619	3616-3_5	GNS	P15586	GNS	7.75E-01	-0.0023	4.60E-01	0.0025	6.62E-01	-0.0016	1.37E-01	0.0068	3.76E-01	0.1325	7.78E-01	-0.0162	4.32E-01	0.0439	3.84E-01	0.0334
620	3617-80_4	HGFA	Q04756	HGFAC	7.58E-03	-0.0312	7.89E-03	-0.0131	1.45E-01	-0.0078	5.79E-02	-0.0127	4.14E-01	0.1804	8.50E-02	0.1457	9.28E-01	-0.0074	8.95E-01	0.0074
621	3620-67_2	IL22RA1	Q8N6P7	IL22RA1	3.64E-01	-0.0092	2.54E-01	-0.0049	2.61E-01	-0.0052	1.91E-01	-0.0075	4.83E-01	0.1327	9.32E-01	0.0062	3.22E-01	0.0699	3.46E-02	0.1019
622	3622-33_4	LGMN	Q99538	LGMN	6.03E-01	0.0055	7.91E-01	-0.0012	3.35E-01	0.0046	9.92E-01	0.0001	1.81E-01	0.2627	1.74E-01	0.1026	4.05E-01	-0.0612	4.61E-02	0.1000
623	3623-84_4	LY86	O95711	LY86	2.67E-01	-0.0112	2.24E-01	-0.0052	7.47E-01	-0.0015	8.98E-02	-0.0097	4.60E-02	0.3753	7.40E-01	0.0241	3.09E-01	-0.0717	7.27E-02	0.0865
624	3624-3_4	Marapsin	Q9BQR3	PRSS27	3.04E-02	-0.0196	1.31E-02	-0.0094	2.41E-01	-0.0048	4.26E-01	-0.0041	6.99E-01	0.0658	1.07E-01	-0.1052	4.50E-01	0.0480	2.31E-01	0.0521
625	3627-71_4	MMEL2	Q495T6	MMEL1	5.24E-01	-0.0068	1.68E-01	-0.0062	9.14E-01	0.0005	3.12E-01	0.0062	1.05E-01	0.3249	1.62E-01	0.1077	6.08E-01	0.0385	3.65E-01	0.0466
626	3628-3_4	MP2K2	P36507	MAP2K2	1.01E-02	0.0295	3.38E-01	0.0047	1.15E-01	0.0083	4.98E-01	0.0045	2.51E-01	0.2482	2.26E-01	0.1006	4.34E-01	-0.0633	7.11E-01	-0.0206
627	3629-60_4	MRCKB	Q9Y5S2	CDC42BPB	8.94E-01	0.0012	2.11E-01	-0.0048	7.98E-01	-0.0011	6.84E-01	0.0021	9.79E-01	0.0045	7.92E-01	0.0174	9.63E-01	0.0030	8.48E-01	0.0084
628	3630-27_4	Nectin-like p	Q8N126	CADM3	2.40E-01	-0.0111	7.00E-02	-0.0072	9.79E-01	-0.0001	6.10E-01	-0.0028	3.44E-01	-0.1679	7.76E-01	0.0195	2.76E-01	0.0721	2.35E-01	-0.0540
629	3633-70_5	NID2	Q14112	NID2	5.10E-01	-0.0076	5.42E-01	-0.0030	1.72E-01	0.0071	6.69E-01	0.0028	1.54E-01	0.3064	1.52E-01	0.1183	6.51E-01	-0.0364	1.83E-01	0.0734
630	3634-5_4	OBCAM	Q14982	OPCML	7.51E-01	0.0032	5.14E-01	-0.0028	5.92E-01	0.0025	8.12E-01	0.0014	8.25E-01	0.0423	3.33E-01	-0.0710	5.49E-02	0.1362	3.83E-03	0.1400
631	3635-76_4	OCAD1	Q9NX40	OCIAD1	2.63E-01	-0.0106	4.50E-02	-0.0080	2.00E-01	-0.0055	4.25E-01	-0.0043	8.09E-01	0.0429	3.11E-01	0.0692	1.76E-01	0.0896	6.84E-01	-0.0186
632	3636-37_4	OLR1	P78380	OLR1	8.59E-01	-0.0018	4.54E-01	0.0032	9.32E-01	0.0004	6.84E-01	0.0023	7.19E-01	-0.0674	7.88E-01	0.0194	5.87E-01	-0.0379	4.37E-01	-0.0372
633	3640-14_3	RAP	P30533	LRPAP1	1.54E-01	-0.0140	2.12E-01	-0.0052	4.38E-01	-0.0035	7.65E-01	-0.0017	1.25E-01	0.2825	6.55E-01	0.0317	7.26E-01	-0.0241	7.79E-01	0.0133
634	3642-4_1	SLAF5	Q9UIB8	CD84	9.31E-03	-0.0126	9.40E-03	-0.0053	3.49E-03	-0.0064	7.89E-01	-0.0007	2.98E-01	0.0949	2.17E-01	-0.0433	1.94E-01	-0.0442	6.56E-01	-0.0104
635	3643-90_2	SLIK1	Q96PX8	SLITRK1	2.40E-01	0.0099	8.18E-01	0.0008	5.60E-01	-0.0022	9.69E-01	-0.0002	8.56E-02	0.2708	4.50E-01	0.0459	2.45E-01	0.0685	8.96E-01	-0.0053
636	3644-5_4	Soggy-1	Q9UK85	DKKL1	5.59E-01	-0.0059	3.14E-01	-0.0043	7.46E-01	-0.0015	6.70E-01	-0.0024	2.49E-01	0.2170	1.98E-01	-0.0931	2.72E-02	0.1543	4.30E-03	0.1363
637	3646-7_4	TEC	P42680	TEC	6.43E-01	-0.0040	2.55E-01	-0.0042	5.23E-01	-0.0025	3.12E-01	-0.0050	5.60E-01	0.0952	2.77E-02	0.1372	8.28E-01	0.0132	7.93E-02	-0.0731
638	3647-49_4	TLR4:MD-2 c	O00206 Q9	TLR4 LY96	7.32E-01	0.0034	3.18E-01	-0.0041	5.14E-01	0.0029	2.34E-01	0.0067	5.32E-01	0.1153	4.97E-01	-0.0482	2.53E-01	0.0786	6.60E-01	-0.0208
639	3651-50_5	VEGF sR2	P35968	KDR	4.49E-01	0.0079	7.20E-01	-0.0016	1.75E-01	0.0065	2.75E-01	0.0065	2.29E-01	-0.2358	4.49E-02	0.1506	7.75E-01	-0.0209	5.53E-01	-0.0299
640	3654-27_5	BMPER	Q8N8U9	BMPER	3.64E-01	-0.0094	3.00E-01	-0.0045	4.84E-01	0.0033	7.97E-01	-0.0015	6.30E-01	-0.0936	6.83E-01	0.0305	8.85E-01	0.0105	9.11E-02	-0.0838
641	3656-9_1	Cadherin-12	P55289	CDH12	6.99E-02	-0.0116	7.01E-03	-0.0072	8.07E-03	-0.0077	1.00E-01	-0.0060	7.11E-02	0.2165	3.99E-01	-0.0390	7.32E-02	-0.0802	4.11E-01	-0.0254
642	3657-74_3	Calcineurin E	P63098	PPP3R1	9.41E-01	0.0007	7.79E-01	-0.0011	9.42E-01	-0.0003	4.03E-01	-0.0046	3.60E-01	0.1633	4.77E-01	-0.0488	3.00E-01	0.0690	8.93E-02	0.0775
643	3666-17_4	complement	Q9BXR6	CFHR5	3.57E-04	0.0372	3.25E-03	0.0130	6.09E-01	-0.0025	4.58E-01	-0.0045	9.59E-01	-0.0102	9.47E-01	0.0050	1.16E-01	-0.1157	7.72E-01	0.0147
644	3676-15_3	IGF-II recept	P11717	IGF2R	1.84E-02	0.0243	1.82E-02	0.0103	7.30E-01	0.0016	4.71E-01	0.0043	4.27E-03	0.5504	8.14E-01	-0.0176	3.50E-01	-0.0678	1.20E-01	0.0773
645	3681-87_3	kallikrein 14	Q9P0G3	KLK14	8.46E-01	-0.0019	9.68E-01	-0.0002	9.05E-01	-0.0005	5.59E-01	-0.0032	2.12E-01	0.2243	8.01E-01	-0.0175	9.21E-01	0.0067	1.75E-01	0.0624
646	3684-78_3	Macrophage	P21757	MSR1	8.40E-01	0.0017	6.61E-01	0.0016	3.53E-01	0.0035	3.22E-01	0.0047	9.47E-01	-0.0105	1.35E-02	0.1482	7.76E-02	0.1032	5.15E-01	-0.0262
647	3685-53_1	MFRP	Q9BY79	MFRP	3.31E-01	-0.0070	1.55E-01	-0.0043	6.67E-01	0.0014	5.71E-01	0.0023	2.71E-02	0.2979	7.87E-01	-0.0141	8.55E-01	-0.0092	3.96E-01	0.0295
648	3707-12_2	Albumin	P02768	ALB	9.53E-01	0.0007	9.53E-01	0.0003	1.86E-01	0.0066	1.86E-01	0.0083	3.52E-01	0.1929	4.08E-01	-0.0658	9.90E-01	0.0009	7.22E-01	-0.0189
649	3708-62_1	a2-Macroglo	P01023	A2M	1.57E-05	-0.0437	1.69E-06	-0.0203	4.43E-02	-0.0094	1.79E-02	-0.0139	1.97E-05	0.8085	5.58E-01	-0.0437	1.18E-03	-0.2315	2.63E-01	-0.0555
650	3709-4_2	ALT	P24298	GPT	7.44E-01	0.0036	9.72E-01	-0.0002	1.61E-01	0.0070	8.88E-01	0.0009	9.09E-01	-0.0236	6.47E-01	0.0365	3.50E-01	0.0723	6.35E-01	-0.0252
651	3710-49_2	Angiostatin	P00747	PLG	1.83E-02	0.0240	2.74E-02	0.0095	4.81E-01	0.0033	8.56E-01	0.0011	3.86E-01	0.1660	3.62E-03	0.2117	8.31E-01	0.0153	1.73E-01	-0.0667
652	3714-49_2	CK-MB	P12277 P06	CKB CKM	9.76E-02	0.0169	8.16E-02	0.0075	6.17E-01	0.0023	4.50E-01	-0.0044	3.85E-02	-0.3953	4.72E-01	0.0530	1.51E-01	0.1026	9.46E-01	0.0033
653	3719-2_2	p27Kip1	P46527	CDKN1B	7.96E-01	-0.0026	6.41E-01	-0.0020	6.47E-01	-0.0021	3.56E-01	-0.0053	4.87E-01	0.1318	1.43E-01	0.1067	4.31E-01	-0.0558	6.12E-01	0.0247
654	3723-1_2	BNP-32	P16860	NPPB	9.31E-01	-0.0009	7.09E-01	-0.0017	4.50E-01	0.0037	9.43E-01	0.0004	6.66E-02	0.3715	5.37E-01	0.0482	1.04E-01	-0.1231	8.96E-01	0.0068
655	3727-35_1	PYY	P10082	PYY	8.54E-01	0.0020	3.43E-01	0.0044	1.93E-01	0.0065	1.39E-01	0.0093	3.53E-01	-0.1916	6.42E-01	-0.0368	6.76E-02	0.1403	1.42E-03	0.1665
656	3728-52_2	Secretin	P09683	SCT	1.44E-01	0.0141	8.68E-02	0.0069	1.12E-02	0.0110	5.80E-01	0.0030	7.29E-01	-0.0624	3.15E-01	0.0696	2.14E-01	0.0835	3.76E-01	0.0409
657	3730-81_2	TNR4	P43489	TNFRSF4	4.50E-01	0.0088	7.09E-01	0.0018	7.70E-01	-0.0015	5.68E-01	-0.0038	8.64E-01	0.0373	8.85E-01	0.0120	2.62E-01	-0.0908	7.21E-01	0.0199
658	3738-54_1	CSF-1	P09603	CSF1	6.57E-01	-0.0037	2.05E-01	-0.0044	8.46E-01	0.0007	8.73E-02	0.0080	9.96E-01	0.0008	2.82E-01	-0.0640	2.78E-01	-0.0627	7.60E-01	-0.0121
659	3758-68_3	Activated Pr	P04070	PROC	3.13E-02	0.0217	5.78E-02	0.0081	2.56E-01	0.0052	5.15E-01	0.0038	4.65E-01	0.1385	4.45E-01	0.0556	2.16E-02	0.1615	5.57E-01	0.0286
660	3761-4_2	COX-2	P35354	PTGS2	1.69E-01	-0.0116	6.82E-01	-0.0015	5.05E-01	-0.0026	8.45E-01	-0.0009	4.08E-01	-0.1304	2.96E-01	-0.0633	9.28E-02	-0.0986	3.40E-01	-0.0385
661	3766-51_4	STX1a	Q16623	STX1A	6.90E-01	0.0045	2.99E-01	0.0049	4.33E-01	0.0040	5.75E-01	0.0036	1.61E-01	-0.2943	9.48E-01	-0.0053	4.10E-01	0.0646	6.71E-01	0.0229
662	3773-15_4	sTie-2	Q02763	TEK	9.19E-03	-0.0258	1.97E-02	-0.0098	3.63E-01	0.0041	4.72E-01	0.0041	4.62E-01	0.1378	3.80E-02	-0.1486	4.20E-01	-0.0563	5.65E-02	0.0911
663	3795-6_2	ADAM 9	Q13443	ADAM9	3.52E-01	-0.0096	4.57E-01	-0.0032	4.69E-01	0.0034	4.29E-01	0.0046	2.45E-02	0.4298	9.16E-01	-0.0078	8.51E-01	-0.0135	3.17E-01	-0.0493
664	3796-79_2	ANGL4	Q9BY76	ANGPTL4	1.61E-01	-0.0087	1.26E-01	-0.0040	6.32E-01	0.0013	5.51E-01	-0.0021	7.80E-01	0.0323	4.18E-01	0.0360	9.17E-01	0.0045	7.04E-01	-0.0113
665	3797-1_1	Cadherin-2	P19022	CDH2	7.94E-01	0.0026	8.12E-01	0.0010	1.92E-01	0.0059	5.68E-01	0.0032	8.31E-01	-0.0397	8.83E-01	0.0106	1.42E-01	0.1019	7.22E-02	0.0855
666	3798-71_1	Carbonic anh	Q16790	CA9	4.07E-01	0.0083	6.46E-01	0.0019	1.98E-01	0.0058	3.01E-01	0.0059	7.59E-01	0.0575	1.25E-01	0.1099	9.09E-01	0.0080	8.84E-03	-0.1242
667	3799-11_2	Carbonic anh	P07451	CA3	2.48E-01	-0.0101	5.48E-01	-0.0022	7.32E-03	0.0106	3.09E-01	0.0051	1.08E-02	-0.4150	5.55E-01	0.0372	2.66E-02	0.1350	9.87E-01	-0.0007
668	3800-71_2	CK-BB	P12277	CKB	7.10E-01	0.0042	9.53E-01	0.0003	7.56E-01	0.0016	9.52E-01	0.0004	5.03E-02	0.4109	3.51E-01	0.0756	2.87E-02	-0.1711	2.43E-01	-0.0629
669	3802-50_1	Cystatin-S	P01036	CST4	8.77E-01	-0.0016	3.50E-01	-0.0040	6.89E-01	0.0019	3.49E-01	0.0054	1.03E-01	0.3092	1.95E-01	0.0947	8.88E-01	-0.0100	1.21E-01	-0.0754
670	3803-10_2	CYTD	P28325	CST5	3.06E-02	-0.0207	5.83E-02	-0.0077	1.39E-01	0.0065	3.79E-01	0.0048	1.40E-01	0.2653	9.55E-01	-0.0039	1.56E-01	-0.0953	2.37E-01	-0.0545
671	3805-16_2	Endocan	Q9NQ30	ESM1	2.07E-05	-0.0473	3.09E-05	-0.0195	3.61E-01	-0.0047	4.72E-01	-0.0047	1.92E-03	0.6521	4.59E-04	-0.2820	1.27E-04	-0.2986	3.34E-01	-0.0525
672	3806-55_1	EphA5	P54756	EPHA5	3.82E-02	-0.0160	3.13E-02	-0.0070	1.11E-01	-0.0056	2.64E-01	-0.0049	7.31E-01	-0.0500	7.35E-01	-0.0190	6.82E-01	-0.0223	4.39E-01	-0.0289
673	3807-1_2	FGF23	Q9GZV9	FGF23	2.69E-01	0.0104	3.17E-01	0.0040	1.93E-01	-0.0056	3.98E-02	-0.0110	2.05E-01	0.2232	7.68E-01	-0.0200	3.27E-01	-0.0645	3.65E-01	0.0409
674	3808-76_2	FGFR-2	P21802	FGFR2	1.07E-01	-0.0168	7.79E-02	-0.0078	2.90E-01	-0.0050	5.02E-02	-0.0117	9.59E-01	-0.0102	6.30E-01	0.0363	2.53E-01	-0.0836	9.05E-01	0.0060
675	3809-1_2	FGFR-3	P22607	FGFR3	6.96E-01	-0.0038	5.60E-01	-0.0024	4.13E-01	-0.0037	9.25E-01	-0.0005	5.66E-01	0.1055	6.88E-02	0.1281	9.35E-01	0.0056	7.69E-01	0.0139
676	3810-50_2	FGR	P09769	FGR	4.72E-01	-0.0073	9.68E-01	0.0002	7.00E-01	-0.0018	6.37E-01	-0.0027	9.32E-01	0.0162	6.96E-01	0.0285	1.18E-01	-0.1102	3.58E-01	0.0446
677	3813-3_2	FYN	P06241	FYN	9.62E-01	0.0004	8.62E-01	-0.0007	3.91E-01	0.0037	7.17E-01	-0.0020	8.81E-01	0.0265	7.82E-01	0.0189	9.34E-01	0.0055	7.23E-01	-0.0161
678	3815-14_1	IL-12 RB2	Q99665	IL12RB2	2.73E-01	0.0122	8.81E-01	-0.0007	5.62E-01	0.0029	8.59E-01	0.0011	4.59E-01	0.1539	1.21E-01	0.1235	6.86E-01	0.0314	9.05E-01	0.0064
679	3817-18_1	KPCT	Q04759	PRKCQ	8.58E-01	0.0019	2.02E-01	-0.0056	1.86E-01	-0.0063	3.13E-01	-0.0061	6.65E-03	0.5300	5.97E-01	0.0401	8.23E-01	-0.0165	6.16E-01	0.0253
680	3820-68_2	MAPK2	P49137	MAPKAPK2	2.34E-01	-0.0106	1.67E-01	-0.0052	4.81E-02	-0.0080	3.41E-01	-0.0049	4.71E-02	0.3304	9.14E-01	0.0070	3.00E-01	-0.0646	1.42E-01	0.0627
681	3821-28_1	MAPK5	Q8IW41	MAPKAPK5	8.56E-01	0.0021	5.13E-01	0.0032	5.43E-01	-0.0032	6.60E-01	-0.0029	6.56E-01	0.0969	1.21E-01	0.1291	4.60E-01	0.0598	5.60E-01	-0.0324
682	3822-54_2	MAPKAPK3	Q16644	MAPKAPK3	6.71E-01	0.0043	8.52E-01	-0.0008	6.42E-01	-0.0022	2.19E-01	-0.0072	4.82E-03	0.5339	7.23E-02	0.1316	7.42E-01	-0.0235	1.54E-01	-0.0696
683	3823-9_1	MATK	P42679	MATK	7.01E-01	-0.0038	4.70E-01	-0.0030	5.77E-01	0.0025	6.94E-01	-0.0022	8.31E-01	0.0390	2.00E-02	0.1624	5.74E-02	0.1292	2.41E-01	-0.0549
684	3825-18_2	MK08	P45983	MAPK8	4.04E-01	-0.0089	3.54E-01	-0.0042	5.73E-01	0.0027	7.33E-01	-0.0021	5.25E-01	0.1265	7.32E-01	0.0262	4.69E-01	-0.0538	1.16E-01	-0.0798
685	3827-22_1	PAK6	Q9NQU5	PAK6	6.28E-01	-0.0035	5.23E-01	-0.0020	1.25E-02	-0.0082	1.64E-02	-0.0099	2.10E-01	0.1709	2.48E-01	0.0606	7.73E-01	0.0147	6.91E-01	0.0139
686	3828-54_2	PDGF-CC	Q9NRA1	PDGFC	1.29E-01	-0.0158	1.83E-01	-0.0058	9.51E-01	-0.0003	5.75E-01	-0.0033	8.18E-01	0.0450	5.72E-01	0.0424	4.68E-01	-0.0529	5.81E-02	-0.0943
687	3831-21_1	pTEN	P60484	PTEN	7.24E-01	0.0038	8.59E-01	-0.0008	5.95E-01	0.0026	4.67E-01	0.0045	8.27E-01	-0.0448	3.19E-02	0.1674	1.41E-01	0.1119	6.03E-01	0.0272
688	3832-51_1	PTK6	Q13882	PTK6	2.67E-01	-0.0130	3.51E-01	-0.0046	6.85E-01	-0.0022	3.47E-01	-0.0063	5.48E-01	-0.1314	4.50E-01	0.0635	5.18E-01	-0.0528	3.80E-01	-0.0492
689	3835-11_2	TLR2	O60603	TLR2	5.52E-01	0.0046	4.28E-01	0.0026	5.71E-01	0.0020	6.73E-01	-0.0019	4.15E-01	-0.1175	8.49E-01	0.0105	1.09E-02	0.1358	3.69E-02	0.0766
690	3836-51_2	UFM1	P61960	UFM1	2.29E-01	-0.0095	1.53E-01	-0.0047	1.67E-01	-0.0049	5.90E-01	0.0024	4.77E-01	-0.1048	6.33E-01	0.0271	9.31E-01	0.0048	4.67E-01	-0.0274
691	3839-60_2	AIP	O00170	AIP	2.20E-02	-0.0144	7.86E-03	-0.0070	2.89E-01	-0.0030	6.53E-01	-0.0016	8.66E-01	-0.0200	9.75E-01	0.0014	5.93E-01	0.0236	8.06E-01	-0.0074
692	3844-2_1	Cyclophilin A	P62937	PPIA	3.33E-01	-0.0048	2.85E-01	-0.0022	7.55E-01	0.0007	9.56E-01	0.0002	7.24E-01	-0.0329	3.59E-01	-0.0328	6.09E-01	0.0178	2.52E-01	-0.0273
693	3845-51_2	DLRB1	Q9NP97	DYNLRB1	5.55E-01	-0.0051	4.41E-01	-0.0028	7.97E-01	0.0010	2.89E-01	-0.0052	4.41E-01	0.1243	6.90E-01	0.0247	9.43E-02	0.1004	1.76E-01	-0.0559
694	3847-56_2	ETHE1	O95571	ETHE1	8.29E-01	-0.0019	7.82E-01	-0.0010	1.23E-01	0.0061	8.14E-01	0.0012	4.12E-01	-0.1332	4.03E-01	-0.0522	1.16E-04	0.2293	8.86E-01	0.0060
695	3848-14_2	GAPDH, liver	P04406	GAPDH	9.00E-01	0.0006	5.45E-01	0.0012	5.52E-02	0.0039	2.61E-02	0.0057	1.35E-02	-0.2086	9.44E-01	0.0023	2.15E-02	0.0726	7.16E-01	-0.0079
696	3852-19_2	HSP 40	P25685	DNAJB1	1.98E-02	-0.0191	4.79E-02	-0.0068	3.74E-02	0.0077	4.68E-01	0.0034	5.68E-01	-0.0880	8.56E-01	0.0107	4.52E-01	0.0432	5.96E-02	-0.0740
697	3853-56_1	MDHC	P40925	MDH1	3.29E-01	0.0060	9.32E-02	0.0044	7.45E-02	0.0050	2.36E-01	0.0042	1.80E-01	-0.1550	8.72E-01	-0.0072	4.79E-02	0.0851	1.20E-01	-0.0459

698	3854-24_1	NACA	Q13765	NACA	7.52E-01	-0.0031	6.19E-01	0.0021	5.94E-01
699	3855-56_1	Peroxiredoxi	Q06830	PRDX1	3.72E-01	-0.0052	6.08E-01	0.0013	3.04E-04
700	3858-5_1	PPAC	P24666	ACP1	3.05E-02	-0.0235	5.04E-02	-0.0090	6.23E-01
701	3859-50_2	PSA1	P25786	PSMA1	8.39E-01	0.0020	9.08E-01	0.0005	1.53E-01
702	3860-7_2	PSA6	P60900	PSMA6	3.47E-02	-0.0101	4.79E-02	-0.0040	4.63E-01
703	3864-5_2	RS7	P62081	RPS7	1.06E-01	0.0135	1.37E-01	0.0052	9.24E-01
704	3865-53_1	RSK-like pro	O75582	RPS6KA5	1.25E-01	-0.0090	1.30E-01	-0.0037	3.44E-01
705	3866-7_2	SBDS	Q9Y3A5	SBDS	3.02E-01	-0.0055	2.46E-01	-0.0026	5.39E-01
706	3867-49_1	SE6L2	Q6UXD5	SEZ6L2	1.34E-01	-0.0107	1.87E-01	-0.0040	7.19E-01
707	3868-8_1	SGTA	O43765	SGTA	4.57E-02	-0.0175	2.49E-01	-0.0043	3.79E-01
708	3872-2_1	TCTP	P13693	TPT1	1.90E-01	0.0072	3.53E-01	0.0021	9.08E-01
709	3873-51_2	TMA	P07202	TPO	1.75E-01	-0.0144	7.20E-02	-0.0081	7.23E-02
710	3874-8_1	UB2L3	P68036	UBE2L3	1.53E-01	-0.0087	1.19E-01	-0.0040	2.21E-01
711	3875-62_1	ARI3A	Q99856	ARID3A	2.19E-01	-0.0099	6.80E-01	-0.0014	4.57E-01
712	3877-67_2	CaMKK alpha	Q8N5S9	CAMKK1	9.79E-01	-0.0001	8.68E-01	0.0004	1.30E-01
713	3879-50_1	CDC37	Q16543	CDC37	2.02E-01	-0.0115	4.67E-01	-0.0028	1.21E-01
714	3881-49_2	DLC8	P63167	DYNLL1	4.92E-01	0.0066	6.22E-01	-0.0020	8.18E-01
715	3887-90_2	IMB1	Q14974	KPNB1	3.77E-02	-0.0116	1.45E-01	-0.0034	4.87E-01
716	3888-8_1	ING1	Q9UK53	ING1	3.34E-01	-0.0042	1.24E-01	-0.0028	4.99E-01
717	3889-64_2	Lamin-B1	P20700	LMNB1	1.22E-01	-0.0129	1.35E-01	-0.0053	6.22E-01
718	3890-8_2	LDH-H 1	P07195	LDHB	5.03E-01	-0.0050	5.38E-01	-0.0019	1.95E-01
719	3891-56_1	MBD4	O95243	MBD4	8.00E-01	-0.0027	1.88E-01	-0.0058	2.68E-01
720	3893-64_1	Mesothelin	Q13421	MSLN	6.93E-01	-0.0040	7.37E-01	-0.0014	6.10E-01
721	3894-15_2	NAGK	Q9UJ70	NAGK	4.76E-01	-0.0063	3.78E-02	-0.0077	8.34E-01
722	3896-5_2	Phosphoglyc	P18669	PGAM1	1.96E-02	0.0259	3.01E-01	0.0049	9.13E-01
723	3897-61_1	PLPP	Q96GD0	PDXP	1.48E-01	-0.0079	5.91E-01	-0.0012	4.27E-02
724	3898-5_2	PSD7	P51665	PSMD7	1.06E-01	-0.0154	6.35E-02	-0.0075	8.60E-01
725	3902-21_2	SKP1	P63208	SKP1	9.76E-02	-0.0092	2.31E-01	-0.0028	5.82E-02
726	3903-49_2	Sorting nexi	O95219	SNX4	5.46E-01	-0.0049	1.48E-01	-0.0049	1.04E-01
727	3905-62_1	UBE2N	P61088	UBE2N	7.06E-01	-0.0021	7.07E-01	0.0009	1.06E-01
728	4122-12_2	discoidin dor	Q08345	DDR1	5.94E-01	-0.0050	5.30E-01	-0.0025	1.48E-01
729	4123-60_2	FGF-4	P08620	FGF4	6.28E-01	0.0047	8.17E-01	0.0010	9.35E-01
730	4124-24_2	HSP 70	P0DMV8	HSPA1A	1.16E-01	0.0174	2.85E-01	0.0050	8.83E-01
731	4125-52_2	sRAGE	Q15109	AGER	2.12E-02	-0.0272	3.23E-03	-0.0146	1.56E-03
732	4126-22_1	BPI	P17213	BPI	5.68E-01	0.0042	5.78E-01	0.0017	7.88E-01
733	4127-75_1	C6	P13671	C6	6.95E-01	0.0027	3.72E-01	-0.0026	9.83E-01
734	4128-27_2	Eotaxin-2	O00175	CCL24	8.24E-02	0.0194	2.57E-01	0.0053	8.11E-01
735	4129-72_1	Factor B	P00751	CFB	8.10E-04	0.0387	9.70E-05	0.0189	6.90E-01
736	4130-71_1	FGF-6	P10767	FGF6	3.90E-01	-0.0066	2.47E-01	-0.0037	9.10E-01
737	4131-72_2	Fibronectin	P02751	FN1	5.24E-02	0.0203	6.47E-02	0.0082	6.61E-01
738	4132-27_2	FST	P19883	FST	1.68E-01	-0.0099	1.28E-01	-0.0046	1.88E-01
739	4133-54_2	Granzyme B	P10144	GZMB	6.17E-01	0.0040	6.86E-01	0.0014	6.37E-01
740	4134-4_2	HB-EGF	Q99075	HBEGF	1.49E-01	0.0157	2.56E-01	0.0052	8.15E-01
741	4135-84_2	IgE	P01854	IGHE IGK@ I	9.29E-01	0.0010	3.89E-01	0.0040	2.02E-01
742	4136-40_2	IL-17D	Q8TAD2	IL17D	9.11E-01	0.0011	8.57E-01	0.0008	5.65E-01
743	4137-57_2	IL-17E	Q9H293	IL25	7.67E-01	0.0029	9.84E-01	0.0001	5.51E-01
744	4138-25_2	IL-20	Q9NYY1	IL20	8.04E-01	0.0028	4.97E-01	-0.0032	8.17E-01
745	4139-71_2	IL-6 sRa	P08887	IL6R	4.42E-01	0.0087	7.39E-01	-0.0016	5.61E-01
746	4140-3_2	IL-7	P13232	IL7	4.35E-01	0.0081	3.28E-01	0.0042	5.35E-01
747	4141-79_1	IP-10	P02778	CXCL10	6.36E-01	0.0051	3.39E-01	0.0043	3.27E-01
748	4143-74_2	Lymphotacti	P47992	XCL1	7.07E-01	0.0044	9.05E-01	0.0006	1.15E-01
749	4144-13_1	MCP-4	Q99616	CCL13	4.48E-01	-0.0075	3.93E-01	-0.0036	1.75E-01
750	4145-58_2	Neurotrophi	P20783	NTF3	7.59E-01	-0.0026	8.39E-01	0.0007	1.78E-01
751	4146-58_2	Neurotrophi	P34130	NTF4	3.64E-01	0.0084	9.20E-01	-0.0004	5.08E-01
752	4148-49_2	PAPP-A	Q13219	PAPPA	9.28E-01	0.0010	4.65E-01	0.0034	9.97E-01
753	4149-8_2	PDGF-BB	P01127	PDGFB	2.80E-02	0.0234	3.70E-02	0.0094	1.07E-01
754	4150-75_2	Plasmin	P00747	PLG	4.41E-02	0.0208	4.92E-01	0.0030	4.03E-01
755	4151-6_2	Plasminoger	P00747	PLG	2.21E-01	0.0128	5.42E-01	0.0027	8.06E-01
756	4152-58_2	Prekallikrein	P03952	KLKB1	1.24E-01	0.0167	1.21E-01	0.0071	9.00E-02
757	4153-11_2	alpha-1-anti	P01011	SERPINA3	7.83E-01	-0.0020	6.89E-01	0.0013	7.30E-01
758	4154-57_2	P-Selectin	P16109	SELP	6.51E-01	-0.0051	7.60E-01	-0.0014	9.69E-01
759	4155-3_2	Tenascin	P24821	TNC	7.02E-01	-0.0044	8.22E-01	0.0011	4.27E-01
760	4156-74_1	TGF-b2	P61812	TGFB2	6.19E-01	0.0051	8.09E-01	0.0011	5.21E-01
761	4157-2_1	Thrombin	P00734	F2	2.94E-01	0.0094	7.08E-01	-0.0014	3.14E-01
762	4158-54_2	uPA	P00749	PLAU	3.58E-01	-0.0101	1.92E-01	-0.0061	3.50E-01
763	4159-130_1	Factor H	P08603	CFH	3.04E-07	0.0575	3.50E-10	0.0292	3.84E-01
764	4160-49_1	MMP-2	P08253	MMP2	4.65E-05	-0.0367	4.77E-03	-0.0108	6.23E-02
765	4162-54_2	Transferrin	P02787	TF	2.45E-05	-0.0455	5.08E-04	-0.0159	2.80E-01
766	4163-5_2	Histone H2A	P0C0S5	H2AFZ	9.76E-01	-0.0003	3.68E-01	0.0032	8.56E-01
767	4165-2_1	Thyroglobuli	P01266	TG	5.90E-01	0.0055	8.28E-01	-0.0009	3.96E-01
768	4179-57_3	14/03/2003	P31946, P62	YWHAB,YWI	7.72E-01	-0.0021	1.97E-01	-0.0039	9.47E-04
769	4184-43_3	4EBP2	Q13542	EIF4EBP2	9.68E-02	0.0183	4.09E-01	0.0038	6.75E-02
770	4187-49_2	6-Phosphogl	P52209	PGD	5.18E-05	-0.0237	1.22E-02	-0.0063	3.87E-01
771	4188-1_2	Aflatoxin B1	O43488	AKR7A2	2.36E-02	-0.0115	1.14E-01	-0.0034	8.63E-01
772	4192-10_2	AK1A1	P14550	AKR1A1	7.77E-01	0.0023	9.01E-01	0.0004	1.91E-02
773	4194-26_3	AN32B	Q92688	ANP32B	5.81E-02	0.0212	5.41E-02	0.0091	5.28E-01
774	4203-50_2	Cofilin-1	P23528	CFL1	1.53E-03	-0.0296	1.10E-02	-0.0101	3.84E-02
775	4209-60_2	DRG-1	Q9NP79	VTA1	3.55E-01	-0.0049	6.16E-01	-0.0011	5.64E-02
776	4212-5_3	EP15R	Q9UBC2	EPS15L1	2.76E-02	-0.0201	3.13E-03	-0.0113	2.37E-01
777	4217-49_3	ERAB	Q99714	HSD17B10	1.51E-01	-0.0130	2.19E-02	-0.0087	8.27E-01
778	4220-39_2	FER	P16591	FER	1.62E-01	-0.0123	4.85E-02	-0.0073	4.38E-01
779	4224-7_2	HNRPQ	O60506	SYNCRIP	7.80E-02	-0.0193	1.98E-02	-0.0107	5.51E-02
780	4230-1_2	IF4G2	P78344	EIF4G2	1.47E-01	-0.0107	8.14E-02	-0.0054	3.20E-01
781	4232-19_2	IGF-I sR	P08069	IGF1R	4.20E-03	-0.0280	7.12E-03	-0.0111	6.43E-01
782	4234-8_2	IL-1 R4	Q01638	IL1RL1	5.20E-01	0.0071	5.46E-01	0.0028	8.02E-01
783	4237-70_3	LCMT1	Q9UIC8	LCMT1	4.93E-01	-0.0071	9.11E-01	-0.0005	6.94E-01
784	4238-4_2	LIN7B	Q9HAP6	LIN7B	4.54E-01	-0.0087	1.37E-01	-0.0072	5.41E-01
785	4240-31_2	M2-PK	P14618	PKM2	6.36E-01	-0.0036	4.55E-01	-0.0024	5.79E-02
786	4245-80_2	MDM2	Q00987	MDM2	7.10E-01	0.0041	6.71E-01	-0.0020	9.51E-01
787	4246-40_2	NCAM-L1	P32004	L1CAM	4.12E-03	-0.0304	3.77E-03	-0.0129	4.57E-03
788	4249-64_2	NDP kinase E	P22392	NME2	1.99E-01	-0.0098	1.60E-01	-0.0045	7.69E-02
789	4250-23_3	NSF1C	Q9UNZ2	NSFL1C	9.08E-01	0.0007	8.84E-01	0.0004	1.32E-02
790	4254-6_2	NUDC3	Q8IVD9	NUDCD3	6.94E-01	0.0036	9.06E-01	0.0005	6.93E-01
791	4258-15_2	PA2G4	Q9UQ80	PA2G4	4.58E-01	-0.0057	7.90E-01	-0.0009	1.24E-01
792	4261-55_2	paraoxonase	P27169	PON1	9.23E-01	0.0011	4.77E-01	-0.0034	9.79E-01
793	4267-81_3	PESC	O00541	PES1	9.12E-01	0.0011	6.14E-01	0.0020	9.85E-01
794	4271-75_2	PFD5	Q99471	PFDN5	4.71E-01	-0.0068	3.28E-01	-0.0039	9.62E-01
795	4272-46_2	PHI	P06744	GPI	5.30E-01	0.0061	9.75E-01	-0.0001	1.00E-01
796	4276-10_2	prostatic bin	P30086	PEBP1	5.83E-01	0.0033	2.59E-01	0.0029	9.75E-03
797	4278-14_3	Protein disul	P07237	P4HB	2.41E-01	-0.0116	2.63E-01	-0.0047	1.43E-01
798	4280-47_2	PSA2	P25787	PSMA2	6.15E-01	-0.0050	8.36E-01	-0.0009	3.58E-01
799	4282-3_2	RAN	P62826	RAN	1.70E-01	-0.0082	9.58E-01	0.0001	6.07E-01
800	4284-18_3	RBM39	Q14498	RBM39	2.70E-01	-0.0108	3.01E-01	-0.0043	2.46E-02
801	4292-5_3	SNAA	P54920	NAPA	8.43E-02	-0.0085	6.73E-02	-0.0038	3.25E-02
802	4294-16_2	Sphingosine	Q9NYA1	SPHK1	2.55E-02	-0.0142	1.48E-03	-0.0085	1.08E-02
803	4297-62_3	Spondin-1	Q9HCB6	SPON1	6.94E-01	-0.0041	5.56E-01	-0.0026	2.00E-01
804	4301-58_2	Thymidine ki	P04183	TK1	8.79E-01	-0.0016	9.04E-01	-0.0005	2.02E-01
805	4304-18_2	transcription	Q8N3X6	LCORL	7.42E-01	0.0033	5.47E-01	0.0026	3.04E-01
806	4306-4_2	Transketolas	P29401	TKT	3.42E-01	-0.0049	3.18E-01	-0.0022	1.79E-02
807	4309-59_3	Triosephospl	P60174	TPI1	2.71E-01	-0.0056	4.70E-01	-0.0016	1.01E-01
808	4314-12_2	XTP3A	Q9H773	DCTPP1	8.74E-01	0.0007	7.83E-01	-0.0005	7.43E-01
809	4318-12_3	PTP-1C	P29350	PTPN6	6.65E-01	-0.0032	4.65E-01	-0.0023	9.36E-02
810	4322-28_3	AMNLS	Q9BXJ7	AMN	9.35E-02	0.0184	1.03E-01	0.0075	4.74E-01
811	4324-33_2	CYTT	P09228	CST2	1.36E-03	-0.0311	1.65E-03	-0.0129	3.33E-01
812	4328-2_2	BOC	Q9BWV1	BOC	4.31E-04	-0.0179	6.53E-04	-0.0073	9.14E-01
813	4332-6_2	CLC1B	Q9P126	CLEC1B	6.34E-02	0.0186	1.64E-01	0.0059	2.85E-01
814	4336-2_1	SAA	P0DJI8	SAA1	1.31E-02	0.0290	3.69E-02	0.0103	5.76E-01
815	4337-49_2	CRP	P02741	CRP	9.30E-10	0.0666	2.44E-10	0.0289	5.80E-01
816	4342-10_3	sICAM-1	P05362	ICAM1	5.95E-03	0.0314	5.26E-02	0.0094	8.22E-01
817	4355-13_1	DAPK2	Q9UIK4	DAPK2	6.07E-01	-0.0053	2.31E-01	-0.0052	4.08E-01
818	4359-87_1	DYRK3	O43781	DYRK3	4.74E-01	-0.0054	5.70E-01	-0.0018	5.33E-01
819	4374-45_2	MIC-1	Q99988	GDF15	8.62E-01	0.0017	9.86E-01	0.0001	3.00E-01
820	4389-2_1	DHH	O43323	DHH	2.86E-01	-0.0071	4.27E-01	-0.0022	7.84E-01
821	4392-54_2	FGF-12	P61328	FGF12	5.05E-01	0.0077	2.42E-01	0.0057	4.65E-01
822	4393-3_1	FGF-16	O43320	FGF16	1.64E-01	-0.0067	5.24E-01	-0.0013	1.12E-01
823	4394-71_2	FGF-8A	P55075	FGF8	1.74E-01	-0.0108	8.79E-02	-0.0057	4.54E-01
824	4396-54_1	IFN-lambda	Q8IU54	IFNL1	1.29E-01	-0.0116	4.18E-01	-0.0026	7.10E-01
825	4397-26_2	IFN-lambda	Q8IZJ0	IFNL2	5.98E-01	0.0060	4.43E-01	0.0037	5.68E-01
826	4407-10_1	MSP	P26927	MST1	6.87E-01	-0.0046	5.77E-01	-0.0027	7.35E-02
827	4413-3_2	SLPI	P03973	SLPI	7.40E-01	-0.0031	8.98E-01	-0.0005	5.07E-01
828	4414-69_2	SP-D	P35247	SFTPD	3.84E-01	-0.0056	7.74E-01	0.0008	7.67E-01
829	4420-7_2	ADAM12	O43184	ADAM12	8.56E-01	-0.0020	9.04E-01	-0.0006	3.47E-01
830	4423-77_2	BCL2-like 1 p	Q07817	BCL2L1	1.33E-02	-0.0216	1.46E-03	-0.0117	9.20E-01
831	4428-1_2	CHST2	Q9Y4C5	CHST2	7.27E-01	-0.0034	2.66E-01	-0.0045	2.92E-01
832	4429-51_2	CHST6	Q9GZX3	CHST6	5.86E-01	0.0060	6.96E-01	0.0018	7.65E-01
833	4430-44_3	Collectin Kid	Q9BWP8	COLEC11	8.03E-01	-0.0026	5.13E-01	-0.0028	6.63E-02
834	4435-66_2	ENPP7	Q6UWV6	ENPP7	2.66E-01	0.0124	3.16E-01	0.0047	3.86E-03
835	4436-1_2	ENTP3	O75355	ENTPD3	4.20E-01	-0.0074	1.41E-01	-0.0057	6.78E-01
836	4437-56_3	ENTP5	O75356	ENTPD5	4.06E-03	0.0317	4.36E-03	0.0132	6.08E-01
837	4440-15_2	FCRL3	Q96P31	FCRL3	2.41E-01	0.0122	5.28E-01	0.0028	1.64E-01
838	4449-67_3	GREM1	O60565	GREM1	6.85E-01	0.0038	8.86E-01	-0.0006	8.24E-01
839	4450-26_2	hnRNP A/B	Q99729	HNRNPAB	2.60E-02	-0.0125	7.14E-02	-0.0043	8.90E-01
840	4452-9_2	LRRT1	Q86UE6	LRRTM1	5.77E-01	-0.0057	3.81E-01	-0.0038	9.61E-01
841	4453-83_3	LRRT3	Q86VH5	LRRTM3	9.68E-01	0.0004	1.74E-01	-0.0051	3.09E-01
842	4455-89_2	MFGM	Q08431	MFGE8	2.58E-01	-0.0082	8.03E-01	-0.0008	3.14E-01
843	4459-68_2	PCSK7	Q16549	PCSK7	2.04E-01	0.0070	4.73E-02	0.0046	4.15E-01
844	4460-8_2	PDPK1	O15530	PDPK1	6.64E-02	-0.0148	5.43E-02	-0.0065	8.67E-01
845	4464-10_2	Sialoadhesin	Q9BZZ2	SIGLEC1	9.57E-01	0.0006	4.78E-01	-0.0031	9.90E-01
846	4467-49_2	SPARCL1	Q14515	SPARCL1	4.65E-01	0.0076	8.78E-01	0.0007	5.13E-01
847	4468-21_2	SPHK2	Q9NRA0	SPHK2	2.24E-01	-0.0128	1.24E-01	-0.0068	5.04E-01
848	4469-78_2	ST4S6	Q7LFX5	CHST15	9.27E-01	0.0009	2.92E-01	-0.0045	7.17E-01
849	4471-50_2	TGM3	Q08188	TGM3	5.51E-01	-0.0070	3.84E-01	-0.0043	6.61E-01
850	4472-5_2	Tropomyosir	P07951	TPM2	6.59E-03	-0.0221	3.65E-03	-0.0099	3.88E-02
851	4474-19_2	Ubiquitin	P62979	RPS27A	3.63E-01	-0.0068	7.43E-01	-0.0010	4.47E-01
852	4476-22_2	ZAP70	P43403	ZAP70	3.25E-03	-0.0278	4.65E-03	-0.0113	1.85E-02
853	4479-14_2	C1-Esterase	P05155	SERPING1	5.01E-01	-0.0072	2.79E-01	-0.0049	8.88E-01
854	4480-59_2	C3b	P01024	C3	3.53E-02	0.0170	3.12E-02	0.0073	8.86E-02
855	4481-34_2	C4	P0C0L4, P0C	C4A C4B	6.96E-01	-0.0047	7.26E-01	-0.0018	6.20E-02
856	4482-66_2	C5b, 6 Comp	P01031,P136	C5 C6	1.43E-01	0.0171	4.25E-02	0.0100	7.65E-01
857	4487-1_1	FGF7	P21781	FGF7	5.37E-01	-0.0056	2.72E-01	-0.0042	1.20E-01
858	4490-65_2	IL-3 Ra	P26951	IL3RA	3.16E-01	-0.0059	2.12E-01	-0.0031	3.00E-01
859	4491-4_2	IL-5 Ra	Q01344	IL5RA	4.50E-01	-0.0062	2.89E-01	0.0037	9.86E-01
860	4493-92_1	IL-11	P20809	IL11	4.25E-01	-0.0078	9.62E-01	0.0002	3.41E-01
861	4495-33_2	Kininogen, H	P01042	KNG1	9.77E-01	-0.0003	5.08E-01	0.0032	5.79E-01
862	4496-60_2	MMP-12	P39900	MMP12	1.84E-01	-0.0139	2.92E-01	-0.0046	3.55E-01
863	4498-62_2	NCAM-120	P13591	NCAM1	1.90E-08	-0.0580	3.19E-06	-0.0205	4.63E-01
864	4499-21_1	PDGF-AA	P04085	PDGFA	6.61E-02	0.0192	3.68E-02	0.0092	7.97E-02
865	4500-50_2	SCGF-alpha	Q9Y240	CLEC11A	3.57E-01	-0.0106	2.48E-01	-0.0056	9.78E-01
866	4533-76_2	ATS15	Q8TE58	ADAMTS15	2.41E-03	0.0286	5.92E-03	0.0109	3.50E-01
867	4534-10_2	BSSP4	Q9GZN4	PRSS22	8.40E-02	-0.0169	5.11E-01	-0.0027	2.80E-02
868	4535-50_2	BST1	Q10588	BST1	8.66E-01	0.0017	8.67E-01	0.0007	1.61E-01
869	4540-11_2	CBX5	P45973	CBX5	8.31E-01	0.0019	5.99E-01	-0.0019	4.37E-01
870	4541-49_2	CDON	Q4KMG0	CDON	4.01E-01	-0.0083	3.56E-01	-0.0038	1.13E-01
871	4542-24_2	Clusterin	P10909	CLU	2.47E-01	-0.0129	2.45E-02	-0.0105	2.95E-01
872	4543-65_2	CONA1	Q86Y22	COL23A1	3.04E-01	-0.0106	8.78E-02	-0.0074	1.76E-01
873	4544-4_3	CTAP-III	P02775	PPBP	5.53E-02	0.0193	7.97E-02	0.0074	9.66E-02
874	4545-53_3	DnaJ homolc	Q96DA6	DNAJC19	3.57E-01	-0.0089	4.22E-01	-0.0033	9.80E-01
875	4546-27_3	EMR2	Q9UHX3	ADGRE2	2.18E-01	-0.0134	3.77E-01	-0.0040	6.12E-01
876	4547-59_2	FLRT1	Q9NZU1	FLRT1	5.09E-03	-0.0219	1.32E-03	-0.0105	1.39E-01
877	4548-4_2	Fucosyltrans	P21217	FUT3	4.69E-01	-0.0079	5.04E-01	-0.0031	1.51E-01
878	4549-78_2	FUT5	Q11128	FUT5	2.54E-02	0.0242	6.20E-02	0.0085	9.37E-01
879	4551-72_3	GP114	Q8IZF4	ADGRG5	3.20E-01	-0.0095	1.03E-01	-0.0066	9.35E-01
880	4553-65_3	HDGR2	Q7Z4V5	HDGFRP2	5.82E-01	0.0037	5.03E-01	0.0019	9.41E-01
881	4556-10_2	IL-34	Q6ZMJ4	IL34	5.81E-01	-0.0044	4.98E-02	-0.0065	4.62E-01
882	4557-61_2	KIRR3	Q8IZU9	KIRREL3	6.00E-01	0.0056	6.51E-01	0.0020	5.41E-01
883	4559-64_2	KYNU	Q16719	KYNU	5.21E-01	0.0072	4.87E-01	0.0033	6.41E-01
884	4561-65_2	Livin B	Q96CA5	BIRC7	6.81E-01	0.0041	9.92E-01	0.0000	1.88E-01
885	4562-1_2	NXPH1	P58417	NXPH1	8.62E-02	0.0186	6.33E-02	0.0085	7.25E-01
886	4563-61_2	PLCG1	P19174	PLCG1	3.10E-03	-0.0328	6.78E-03	-0.0127	4.57E-01
887	4564-2_2	PLXC1	O60486	PLXNC1	9.23E-02	-0.0181	1.60E-01	-0.0064	6.38E-02
888	4566-24_3	RSPO2	Q6UXX9	RSPO2	2.04E-01	0.0085	3.11E-03	0.0083	5.24E-01
889	4567-82_2	SH21A	O60880	SH2D1A	6.10E-02	-0.0131	1.71E-01	-0.0040	5.85E-01
890	4568-17_2	SLIK5	O94991	SLITRK5	2.74E-03	-0.0149	1.08E-02	-0.0053	1.70E-03
891	4569-52_2	SORC2	Q96PQ0	SORCS2	5.06E-01	0.0061	2.04E-01	-0.0049	2.45E-01
892	4588-1_2	PH	P01298	PPY	9.93E-01	-0.0001	4.87E-01	0.0030	4.52E-02
893	4673-13_2	IL-6	P05231	IL6	1.21E-02	0.0254	1.99E-02	0.0099	6.20E-01
894	4693-72_2	3HIDH	P31937	HIBADH	9.03E-01	0.0012	5.87E-01	0.0023	7.89E-02
895	4697-59_2	GM-CSF	P04141	CSF2	1.44E-01	-0.0158	3.61E-01	-0.0042	5.06E-01
896	4703-87_2	TNF-b	P01374	LTA	7.07E-01	0.0030	8.83E-01	-0.0005	1.53E-01
897	4706-17_2	41	P11171	EPB41	4.54E-01	-0.0048	6.87E-01	-0.0011	3.44E-01
898	4708-3_2	17-beta-HSD	P14061	HSD17B1	5.86E-01	-0.0047	4.00E-01	-0.0031	7.42E-01
899	4712-28_2	Apo D	P05090	APOD	1.19E-01	-0.0110	8.33E-02	-0.0051	4.36E-01
900	4717-55_2	IL-3	P08700	IL3	3.38E-01	-0.0095	6.60E-01	-0.0018	5.57E-01
901	4718-5_2	PPIB	P23284	PPIB	6.01E-01	0.0037	8.94E-01	0.0004	7.36E-01
902	4719-58_2	Protein disul	P30101	PDIA3	7.38E-01	0.0024	5.17E-01	-0.0020	6.30E-01
903	4721-54_2	TFF3	Q07654	TFF3	1.88E-03	-0.0266	2.10E-03	-0.0111	9.91E-01
904	4763-31_3	Afamin	P43652	AFM	1.62E-01	0.0146	4.74E-02	0.0087	2.17E-01
905	4769-10_3	Olfactomedi	Q6UX06	OLFM4	1.07E-01	0.0178	1.55E-01	0.0066	7.83E-01
906	4771-10_3	ASM3A	Q92484	SMPDL3A	8.03E-01	-0.0021	2.08E-01	-0.0044	6.40E-01
907	4774-62_3	FAM107B	Q9H098	FAM107B	9.12E-05	0.0190	1.81E-05	0.0087	3.81E-01
908	4775-34_3	Gelsolin	P06396	GSN	3.54E-01	-0.0108	7.38E-02	-0.0088	5.16E-01
909	4785-30_3	CBG	P08185	SERPINA6	3.29E-01	-0.0079	8.40E-01	-0.0007	9.86E-01
910	4786-58_3	Cytidylate ki	P30085	CMPK1	5.00E-02	-0.0137	1.40E-01	-0.0044	5.13E-01
911	4792-51_2	C34 gp41 HI\	Q70626	Human-virus	1.09E-01	-0.0184	1.37E-01	-0.0072	7.99E-01
912	4801-13_3	PERL	P22079	LPO	7.65E-02	-0.0149	7.33E-02	-0.0064	3.54E-01
913	4807-13_3	CO8A1	P27658	COL8A1	5.53E-01	-0.0062	7.86E-02	-0.0077	2.64E-01
914	4811-33_3	ITI heavy cha	Q14624	ITIH4	4.90E-01	0.0079	6.58E-01	0.0021	2.91E-01
915	4815-25_3	TXD12	O95881	TXNDC12	6.39E-01	0.0055	9.98E-01	0.0000	8.84E-01
916	4829-43_6	STRATIFIN	P31947	SFN	5.38E-01	0.0061	3.83E-01	-0.0036	4.97E-01
917	4831-4_2	sL-Selectin	P14151	SELL	9.56E-04	-0.0371	1.38E-03	-0.0151	2.67E-02
918	4832-75_2	TRAIL R1	O00220	TNFRSF10A	9.36E-01	-0.0009	6.96E-01	-0.0018	4.86E-01
919	4834-61_2	Epithelial ce	P29317	EPHA2	5.43E-01	-0.0036	3.53E-01	-0.0023	1.00E-01
920	4840-73_1	G-CSF	P09919	CSF3	8.45E-01	-0.0016	7.67E-01	0.0010	6.76E-01
921	4842-62_2	Glypican 3	P51654	GPC3	9.30E-02	-0.0153	5.51E-03	-0.0106	4.59E-01
922	4851-25_1	IL-1a	P01583	IL1A	3.19E-01	0.0101	6.63E-01	0.0019	9.39E-01
923	4859-6_1	BMPR1A	P36894	BMPR1A	4.69E-05	-0.0442	6.62E-06	-0.0205	7.57E-01
924	4862-63_1	BMP RII	Q13873	BMPR2	1.36E-01	-0.0161	6.07E-02	-0.0085	8.29E-01
925	4866-59_2	TrkB	Q16620	NTRK2	2.98E-01	0.0103	9.74E-01	0.0001	7.00E-02
926	4867-15_2	VEGF121	P15692	VEGFA	9.05E-02	0.0196	2.50E-03	0.0146	2.27E-01
927	4874-3_1	Angiogenin	P03950	ANG	9.64E-01	0.0005	4.30E-01	-0.0037	1.08E-01
928	4876-32_1	Coagulation	P00740	F9	1.33E-06	0.0548	8.50E-08	0.0254	1.24E-03
929	4878-3_1	Coagulation	P00742	F10	1.05E-02	0.0295	4.35E-04	0.0169	7.94E-02
930	4880-21_1	GDF2	Q9UK05	GDF2	1.75E-03	-0.0333	2.09E-04	-0.0165	1.39E-01
931	4883-56_2	Insulin	P01308	INS	8.64E-06	0.0441	2.35E-08	0.0230	2.85E-01
932	4886-3_1	MCP-3	P80098	CCL7	1.72E-02	0.0184	2.09E-02	0.0075	9.05E-02

[illegible]

933	4889-82_1	WNT7A	O00755	WNT7A	1.06E-01	0.0160	1.87E-01	0.0055	8.74E-01	0.0007	2.30E-01	-0.0068	7.52E-01	-0.0589	1.67E-03	0.2221	5.58E-01	-0.0407	4.51E-01	-0.0359
934	4890-10_1	ACTH	P01189	POMC	5.53E-02	0.0217	2.65E-02	0.0105	6.85E-01	0.0021	3.38E-01	0.0062	3.70E-01	-0.1904	6.95E-01	0.0320	2.02E-01	0.1010	4.94E-01	0.0372
935	4891-50_1	Glucagon	P01275	GCG	6.11E-01	-0.0051	6.49E-01	0.0019	9.82E-01	0.0001	4.64E-01	0.0041	7.13E-01	-0.0685	1.71E-01	-0.0976	5.06E-02	0.1350	6.45E-03	0.1285
936	4900-8_1	C3a	P01024	C3	5.06E-03	0.0265	3.96E-03	0.0115	3.05E-02	0.0093	2.46E-01	0.0063	8.94E-02	-0.3027	4.60E-01	0.0507	8.86E-02	0.1133	8.77E-02	0.0779
937	4903-72_1	Calcineurin	Q08209 P63	PPP3CA PPP3	5.26E-01	-0.0059	1.36E-01	-0.0059	4.23E-01	-0.0034	2.82E-01	-0.0057	1.14E-01	0.2751	6.34E-03	0.1816	2.80E-01	-0.0704	1.14E-01	-0.0706
938	4904-7_1	Caspase-2	P42575	CASP2	2.19E-01	0.0123	6.15E-01	0.0021	4.92E-01	0.0031	4.88E-01	0.0040	3.94E-03	0.5372	7.02E-01	0.0277	3.11E-01	-0.0711	6.51E-02	0.0886
939	4905-63_1	Coactosin-lik	Q14019	COTL1	6.25E-01	-0.0034	6.91E-01	-0.0012	6.28E-01	0.0015	7.47E-01	0.0013	9.17E-01	-0.0137	9.70E-01	-0.0019	9.50E-01	0.0031	7.91E-01	-0.0089
940	4906-35_1	Coagulation	P12259	F5	1.66E-01	-0.0151	4.49E-01	-0.0035	5.10E-01	0.0033	2.55E-01	0.0071	8.37E-01	0.0420	6.22E-01	0.0386	7.59E-02	0.1346	5.74E-01	0.0293
941	4907-56_1	D-dimer	P02671 P026	FGA FGB FG	1.09E-01	0.0153	4.66E-01	0.0029	6.37E-01	-0.0021	1.61E-02	-0.0130	3.59E-01	-0.1640	6.01E-01	0.0360	3.46E-01	-0.0629	7.32E-01	-0.0157
942	4908-6_1	Endoglin	P17813	ENG	1.55E-01	-0.0055	1.50E-01	-0.0024	8.28E-01	-0.0004	6.74E-01	0.0009	8.59E-01	0.0129	2.70E-01	-0.0308	4.17E-01	0.0220	2.00E-01	-0.0239
943	4909-68_1	Galectin-8	O00214	LGALS8	1.53E-01	0.0147	1.58E-02	0.0104	4.01E-01	0.0039	5.47E-01	0.0035	6.86E-02	-0.3497	5.78E-02	0.1399	9.44E-02	0.1200	8.72E-02	0.0842
944	4910-21_1	GIB	P04054	PLA2G1B	3.54E-02	-0.0188	1.81E-02	-0.0089	9.98E-01	0.0000	6.04E-01	0.0027	7.38E-01	0.0562	4.54E-01	0.0484	7.02E-01	0.0240	4.69E-01	0.0312
945	4911-49_2	Glutathione	P09211	GSTP1	8.98E-01	0.0009	5.78E-01	-0.0017	3.97E-02	0.0067	5.25E-01	0.0026	1.47E-01	-0.1949	6.39E-01	-0.0243	8.36E-01	-0.0104	5.88E-02	-0.0649
946	4912-17_1	GOT1	P17174	GOT1	1.65E-01	0.0125	3.55E-02	0.0080	3.97E-01	0.0035	6.15E-01	-0.0026	1.18E-01	-0.2632	1.21E-01	0.1005	8.77E-01	0.0097	1.64E-01	0.0601
947	4913-78_1	HCC-4	O15467	CCL16	1.32E-03	0.0331	6.93E-04	0.0147	4.08E-03	0.0135	2.14E-02	0.0136	2.79E-01	-0.2113	4.26E-01	0.0597	2.56E-02	0.1618	5.44E-01	-0.0303
948	4914-10_1	HCG	P01215,P012	CGA CGB	1.37E-01	-0.0145	1.65E-01	-0.0057	4.75E-01	0.0032	8.28E-01	0.0012	5.73E-02	0.3477	1.71E-01	0.0964	3.32E-01	0.0665	6.22E-01	-0.0232
949	4915-64_2	Hemoglobin	P69905, P68	HBA1 HBB	4.12E-04	-0.0273	1.25E-02	-0.0082	2.48E-01	0.0041	6.30E-01	0.0022	7.78E-01	-0.0416	6.51E-01	-0.0256	8.58E-02	0.0940	1.63E-01	-0.0525
950	4916-2_1	IgD	P01880	IGHD IGK@	3.80E-01	-0.0103	6.32E-01	-0.0024	6.94E-01	0.0021	8.64E-01	-0.0012	9.09E-02	0.3714	7.06E-01	-0.0319	1.40E-01	-0.1212	1.25E-01	-0.0863
951	4917-62_1	Integrin aVb	P06756, P18	ITGAV ITGB!	9.58E-01	0.0005	6.61E-01	-0.0018	8.71E-02	-0.0075	1.97E-01	-0.0071	3.45E-01	0.1711	3.36E-01	0.0670	7.96E-02	-0.1181	8.68E-01	0.0077
952	4920-10_1	Lysozyme	P61626	LYZ	7.45E-01	-0.0034	4.75E-01	-0.0032	6.41E-01	-0.0022	2.20E-01	-0.0074	1.58E-03	-0.6183	1.66E-01	-0.1051	3.92E-01	-0.0632	1.28E-02	-0.1254
953	4922-13_1	MIP-3b	Q99731	CCL19	4.60E-01	0.0068	4.22E-01	0.0031	5.18E-01	-0.0027	8.61E-01	-0.0009	3.64E-01	-0.1553	1.64E-01	-0.0913	1.20E-01	-0.0991	5.71E-02	0.0831
954	4923-79_1	MIS	P03971	AMH	7.25E-02	0.0133	2.96E-02	0.0068	8.99E-01	0.0004	6.42E-01	-0.0020	1.03E-02	-0.3546	1.57E-01	0.0756	7.35E-01	-0.0176	3.24E-01	-0.0352
955	4924-32_1	MMP-1	P03956	MMP1	2.33E-01	0.0132	1.48E-02	0.0113	2.79E-01	0.0054	3.42E-01	0.0060	7.44E-01	-0.0679	7.48E-01	0.0257	8.63E-02	0.1324	2.37E-03	0.1597
956	4925-54_2	MMP-13	P45452	MMP13	4.57E-01	-0.0075	7.68E-01	-0.0013	8.89E-01	0.0006	3.87E-01	0.0050	9.32E-01	0.0161	4.38E-01	0.0560	9.40E-01	0.0053	6.08E-01	-0.0247
957	4929-55_1	SHBG	P04278	SHBG	8.21E-07	-0.0503	8.46E-10	-0.0259	4.73E-01	-0.0034	6.70E-01	-0.0026	6.02E-10	1.1619	2.92E-01	-0.0797	2.59E-03	-0.2191	7.05E-02	-0.0910
958	4930-21_1	Stanniocalci	P52823	STC1	7.05E-01	0.0042	4.66E-01	0.0034	9.29E-01	0.0005	8.01E-01	0.0016	2.49E-01	0.2389	5.32E-01	0.0498	1.54E-01	-0.1103	3.20E-01	-0.0528
959	4931-59_1	TF	P13726	F3	7.34E-01	0.0031	8.97E-01	-0.0005	5.11E-01	0.0027	9.14E-01	-0.0006	1.34E-01	-0.2524	5.23E-01	-0.0414	4.03E-01	0.0526	3.68E-01	0.0388
960	4956-2_1	EPI	O14944	EREG	3.32E-01	0.0104	5.93E-01	0.0024	8.73E-01	-0.0008	7.05E-01	0.0023	9.80E-01	-0.0051	2.23E-01	0.0936	8.78E-01	0.0114	7.05E-01	-0.0194
961	4957-1_2	40S ribosom	P08865	RPSA	7.42E-01	-0.0032	4.32E-01	-0.0032	5.57E-01	-0.0026	4.66E-02	-0.0110	5.56E-01	-0.1071	3.55E-01	0.0645	6.59E-02	0.1243	9.06E-01	-0.0055
962	4959-2_1	AGR2	O95994	AGR2	6.72E-01	0.0035	8.75E-01	0.0005	7.02E-01	-0.0014	6.24E-01	-0.0023	9.87E-01	0.0026	3.30E-01	-0.0575	7.81E-01	-0.0159	2.09E-03	0.1197
963	4960-72_1	annexin I	P04083	ANXA1	5.01E-01	-0.0057	4.31E-01	-0.0028	9.21E-01	0.0004	9.37E-01	-0.0004	7.94E-01	-0.0410	2.46E-01	0.0701	4.13E-01	-0.0480	5.57E-01	-0.0237
964	4961-17_1	annexin II	P07355	ANXA2	5.61E-01	-0.0042	4.85E-01	-0.0021	1.46E-01	0.0048	8.89E-01	0.0006	5.18E-03	0.3770	4.68E-01	-0.0380	1.56E-02	-0.1221	8.28E-01	0.0076
965	4962-52_1	ARMEL	Q49AH0	CDNF	4.04E-03	-0.0309	5.46E-03	-0.0126	5.22E-01	0.0032	9.66E-01	0.0003	3.87E-01	-0.1756	8.80E-01	0.0118	5.85E-01	0.0413	7.42E-02	-0.0925
966	4963-19_1	ARP19	P56211	ARPP19	6.48E-01	0.0036	3.96E-01	0.0028	2.83E-01	-0.0038	4.33E-01	-0.0035	6.07E-01	-0.0760	9.75E-02	0.0937	1.75E-01	0.0745	9.35E-01	0.0031
967	4964-67_1	ARTS1	Q9NZ08	ERAP1	8.09E-01	-0.0016	8.29E-01	-0.0006	4.96E-02	-0.0058	5.15E-02	-0.0072	9.23E-01	0.0118	4.52E-01	0.0353	8.87E-01	-0.0065	4.08E-01	-0.0259
968	4965-27_1	ATP synthase	P06576	ATP5B	1.23E-01	-0.0167	2.52E-01	-0.0052	2.28E-01	-0.0059	9.87E-02	-0.0102	2.27E-01	0.2447	4.00E-01	-0.0655	8.32E-01	0.0160	4.75E-02	0.1024
969	4967-1_1	C1QBP	Q07021	C1QBP	9.55E-01	-0.0006	9.37E-01	0.0003	4.82E-01	0.0033	3.52E-01	-0.0055	5.22E-01	0.1247	1.60E-01	0.1049	3.80E-01	0.0638	9.69E-01	0.0019
970	4968-50_1	CAPG	P40121	CAPG	4.47E-02	0.0223	1.05E-01	0.0076	2.29E-01	0.0061	6.49E-01	0.0029	6.90E-01	-0.0833	3.62E-01	0.0730	8.85E-01	-0.0112	7.87E-01	-0.0145
971	4969-2_1	Carbonic anh	P00915	CA1	1.75E-02	-0.0197	2.78E-01	-0.0038	1.41E-02	0.0092	9.08E-03	0.0123	3.30E-02	-0.3311	8.98E-01	-0.0077	8.17E-02	0.1011	6.54E-01	-0.0179
972	4970-55_1	carbonic anh	P00918	CA2	3.15E-01	0.0101	2.30E-01	0.0051	9.76E-01	-0.0001	9.65E-01	-0.0003	6.54E-01	-0.0847	8.34E-01	-0.0153	2.48E-01	0.0814	6.60E-01	-0.0213
973	4971-1_1	CATZ	Q9UBR2	CTSZ	8.37E-02	0.0188	3.35E-01	0.0044	1.67E-01	0.0068	7.78E-02	0.0109	3.04E-01	0.2092	8.14E-01	0.0184	1.28E-02	0.1880	3.50E-03	0.1508

[illegible]

1168	5457-5_2	COLEC12	Q5KU26	COLEC12	5.45E-03	-0.0201	1.42E-02	-0.0075	8.76E-01	0.0005	7.29E-01	-0.0014	5.48E-01	0.0822	3.18E-01	0.0525	7.98E-01	0.0131	9.94E-01	-0.0003
1169	5459-33_3	CYTN	P01037	CST1	1.39E-02	-0.0268	8.48E-03	-0.0121	1.20E-01	-0.0077	5.17E-02	-0.0121	8.50E-01	0.0388	7.56E-02	0.1396	8.51E-01	-0.0144	4.05E-01	-0.0437
1170	5460-60_3	DEAD-box pr	Q9UMR2	DDX19B	7.71E-01	-0.0026	6.13E-01	-0.0019	8.18E-01	0.0009	9.67E-01	0.0002	5.40E-01	0.1023	4.26E-01	0.0511	4.42E-01	0.0480	1.92E-01	0.0558
1171	5462-62_3	Ficolin-3	O75636	FCN3	9.14E-02	0.0199	2.99E-01	0.0052	1.45E-01	-0.0078	7.33E-01	-0.0023	7.99E-01	-0.0564	2.31E-01	0.1019	8.01E-01	-0.0208	3.04E-01	0.0583
1172	5463-22_3	GAS1	P54826	GAS1	5.33E-02	-0.0200	3.94E-02	-0.0090	5.56E-01	-0.0028	2.54E-01	0.0068	6.31E-02	0.3600	2.44E-01	-0.0870	3.63E-01	-0.0660	8.00E-01	0.0126
1173	5464-52_3	GRB2 adapt	P62993	GRB2	8.90E-01	-0.0006	3.64E-01	-0.0016	2.52E-01	-0.0022	1.18E-01	-0.0038	2.03E-01	0.1006	1.05E-01	0.0492	6.40E-02	-0.0545	2.67E-01	-0.0225
1174	5465-32_3	H6ST1	O60243	HS6ST1	6.79E-01	-0.0039	4.53E-01	-0.0029	1.49E-01	-0.0061	6.71E-01	-0.0023	1.42E-01	0.2552	2.32E-01	-0.0799	6.84E-01	-0.0265	3.14E-01	-0.0449
1175	5467-15_3	HSP 90b	P08238	HSP90AB1	8.28E-01	0.0015	4.93E-01	-0.0020	5.27E-01	0.0020	1.22E-01	0.0062	6.22E-01	0.0655	8.17E-01	-0.0118	7.50E-02	0.0878	7.75E-01	-0.0097
1176	5468-67_3	IL-17 RC	Q8NAC3	IL17RC	3.15E-01	0.0108	5.57E-01	0.0027	5.47E-01	0.0029	6.04E-01	0.0032	2.95E-02	-0.4359	6.40E-01	-0.0362	1.19E-01	0.1169	2.68E-01	0.0571
1177	5470-69_2	MED-1	Q15648	MED1	1.19E-01	-0.0150	2.13E-01	-0.0050	2.36E-02	0.0098	2.99E-02	0.0118	5.60E-01	0.1050	9.94E-01	-0.0005	1.93E-01	0.0872	7.82E-01	0.0128
1178	5475-10_3	PKC-B-II	P05771	PRKCB	3.45E-01	-0.0064	2.45E-01	-0.0033	1.71E-03	-0.0096	6.20E-02	-0.0072	1.24E-01	0.1959	3.40E-01	0.0468	1.41E-01	-0.0700	5.58E-01	-0.0192
1179	5476-66_3	PKC-G	P05129	PRKCG	8.63E-01	0.0017	5.55E-01	0.0025	1.60E-01	-0.0063	1.82E-01	-0.0076	2.05E-01	-0.2353	2.64E-01	0.0798	5.82E-01	0.0382	9.08E-01	0.0055
1180	5478-50_2	PSMA	Q04609	FOLH1	5.06E-01	0.0075	5.97E-01	0.0025	7.98E-01	0.0013	8.42E-01	-0.0013	1.18E-02	0.5269	1.51E-01	0.1160	7.47E-01	0.0254	7.35E-01	-0.0182
1181	5480-49_3	RANTES	P13501	CCL5	3.33E-02	0.0231	6.61E-02	0.0084	1.96E-01	0.0064	6.66E-01	0.0027	2.50E-01	0.2340	8.96E-02	0.1325	1.30E-01	0.1149	1.54E-02	0.1255
1182	5481-16_3	RASA1	P20936	RASA1	4.24E-02	0.0193	1.41E-01	0.0059	2.75E-01	0.0047	5.91E-01	0.0029	7.56E-01	-0.0558	5.86E-01	0.0375	3.00E-01	0.0692	9.25E-01	0.0043
1183	5482-61_3	RBP	P02753	RBP4	8.37E-02	-0.0189	1.04E-01	-0.0075	3.37E-03	0.0145	1.86E-02	0.0147	5.21E-02	0.3977	6.51E-01	0.0357	4.98E-02	0.1499	9.47E-01	-0.0035
1184	5483-1_3	RGMA	Q96B86	RGMA	1.29E-01	-0.0157	3.18E-01	-0.0044	6.68E-01	-0.0020	7.58E-01	-0.0018	9.39E-01	0.0150	7.09E-01	-0.0279	2.13E-01	0.0902	7.93E-01	-0.0131
1185	5484-63_3	RS3A	P61247	RPS3A	9.27E-01	0.0009	6.90E-01	0.0016	1.19E-01	-0.0067	1.08E-01	-0.0086	8.43E-01	0.0352	9.13E-01	-0.0074	3.07E-01	-0.0675	6.32E-01	0.0217
1186	5486-73_3	sICAM-2	P13598	ICAM2	1.79E-01	-0.0132	1.95E-01	-0.0054	2.62E-01	-0.0050	9.90E-01	0.0001	1.22E-01	-0.2846	1.83E-01	-0.0943	2.06E-01	-0.0869	5.87E-01	0.0257
1187	5487-7_3	SLAF7	Q9NQ25	SLAMF7	4.03E-01	0.0090	9.73E-01	0.0002	8.09E-01	0.0012	1.61E-01	0.0086	7.12E-01	-0.0741	2.73E-01	-0.0846	8.43E-01	-0.0149	1.34E-01	0.0769
1188	5488-74_3	SRCN1	P12931	SRC	2.77E-02	-0.0176	4.04E-02	-0.0069	2.79E-04	-0.0131	5.42E-02	-0.0088	1.70E-01	0.2060	1.70E-01	0.0792	2.26E-01	-0.0678	5.53E-01	-0.0228
1189	5489-18_3	Stress-induc	P31948	STIP1	1.80E-01	-0.0078	7.41E-01	-0.0008	3.25E-02	0.0057	3.84E-02	0.0069	6.81E-02	-0.1993	3.31E-01	-0.0409	6.52E-02	0.0752	3.51E-01	-0.0262
1190	5490-53_3	Testican-1	Q08629	SPOCK1	5.82E-01	0.0044	1.76E-01	0.0046	4.12E-01	0.0030	6.56E-01	0.0020	4.28E-01	0.1194	7.15E-01	-0.0212	7.75E-01	0.0161	2.85E-02	0.0841
1191	5491-12_3	Testican-2	Q92563	SPOCK2	1.23E-01	0.0171	4.05E-01	0.0039	9.99E-01	0.0000	8.41E-02	-0.0109	4.82E-01	-0.1462	5.54E-01	0.0473	7.49E-01	-0.0249	5.63E-01	-0.0308
1192	5493-17_3	WNK3	Q9BYP7	WNK3	6.35E-01	-0.0027	9.63E-01	0.0001	2.42E-03	0.0078	3.68E-03	0.0094	4.03E-03	-0.3043	1.04E-01	-0.0666	1.85E-02	0.0933	8.81E-02	-0.0465
1193	5494-52_3	RUXF	P62306	SNRPF	1.46E-01	-0.0154	2.57E-01	-0.0051	7.41E-01	-0.0016	8.20E-01	0.0014	6.33E-01	-0.0947	3.84E-01	0.0664	2.01E-02	0.1712	4.97E-01	-0.0346
1194	5508-62_3	Cathepsin D	P07339	CTSD	7.75E-01	-0.0032	6.33E-01	-0.0023	7.11E-02	0.0092	1.98E-01	0.0082	7.19E-01	-0.0755	6.83E-01	-0.0329	5.02E-03	0.2179	7.13E-01	0.0198
1195	5509-7_3	EGF	P01133	EGF	1.02E-01	-0.0182	4.15E-01	-0.0038	4.36E-02	-0.0102	3.52E-01	-0.0059	5.96E-01	0.1110	4.62E-01	0.0591	8.44E-01	0.0153	5.14E-01	-0.0350
1196	5526-53_3	GITR	Q9Y5U5	TNFRSF18	1.42E-04	0.0331	6.98E-05	0.0145	8.07E-01	0.0010	5.73E-01	-0.0028	4.54E-01	-0.1240	4.72E-01	-0.0458	5.58E-01	0.0363	9.83E-01	0.0009
1197	5532-53_3	bFGF-R	P11362	FGFR1	1.46E-03	-0.0371	8.49E-04	-0.0164	6.09E-02	-0.0100	5.69E-01	-0.0038	4.37E-01	0.1720	3.94E-02	-0.1742	3.52E-02	-0.1729	5.89E-01	-0.0306
1198	5534-49_2	TRAIL R2	O14763	TNFRSF10B	9.28E-01	0.0008	8.25E-01	-0.0008	4.46E-01	0.0031	8.01E-02	0.0089	1.39E-01	0.2471	1.51E-01	0.0922	1.49E-01	-0.0900	9.64E-01	-0.0020
1199	5542-22_2	NRP1	O14786	NRP1	5.52E-02	-0.0219	2.95E-03	-0.0142	8.29E-01	-0.0011	4.89E-01	-0.0045	7.78E-02	0.3779	1.95E-01	-0.1070	1.65E-06	-0.3734	2.86E-01	-0.0587
1200	5792-8_2	AFP	P02771	AFP	7.06E-01	-0.0040	7.67E-01	0.0013	5.44E-01	0.0029	6.10E-01	-0.0031	7.58E-01	0.0612	3.76E-01	0.0674	3.31E-01	0.0719	3.07E-01	0.0518
1201	5798-3_3	BID	P55957	BID	9.48E-01	-0.0006	9.81E-01	-0.0001	5.41E-02	0.0086	7.09E-01	0.0021	1.32E-01	0.2769	4.08E-01	0.0587	8.30E-01	0.0148	6.09E-01	0.0242
1202	5801-72_3	b-NGF	P01138	NGF	6.92E-01	0.0041	2.50E-01	0.0050	9.59E-02	0.0079	1.30E-02	0.0147	5.19E-01	-0.1259	4.56E-01	0.0559	1.97E-01	0.0937	8.00E-01	-0.0126
1203	5803-24_3	C3d	P01024	C3	1.25E-03	0.0213	2.24E-03	0.0085	6.25E-01	0.0015	8.00E-01	0.0010	1.96E-01	-0.1612	2.98E-01	0.0500	5.93E-01	-0.0249	1.82E-01	-0.0427
1204	5807-77_3	CD70	P32970	CD70	4.75E-01	-0.0074	2.84E-01	-0.0047	3.93E-02	0.0097	3.89E-02	0.0122	8.73E-02	0.3318	9.93E-02	0.1230	5.08E-01	0.0481	8.52E-01	0.0093
1205	5810-25_3	Cripto	P13385	TDGF1	6.93E-01	0.0045	7.48E-01	-0.0015	7.58E-01	0.0016	5.31E-01	0.0041	6.77E-02	-0.3873	8.72E-01	-0.0132	9.28E-01	0.0072	2.02E-01	-0.0695
1206	5813-58_3	Epo	P01588	EPO	6.69E-03	0.0223	2.76E-03	0.0104	3.36E-01	-0.0036	2.44E-01	-0.0055	7.12E-01	0.0574	2.41E-01	-0.0701	6.15E-01	-0.0292	4.30E-01	0.0315
1207	5822-22_3	GDNF	P39905	GDNF	5.29E-01	-0.0062	5.47E-01	-0.0025	5.09E-01	-0.0029	1.79E-01	-0.0075	1.97E-02	0.4263	6.94E-03	0.1892	6.30E-01	0.0331	8.02E-01	-0.0118
1208	5825-49_3	IFN-g R1	P15260	IFNGR1	7.44E-01	0.0027	5.99E-01	0.0019	4.51E-01	-0.0029	4.75E-01	0.0034	9.04E-01	0.0190	9.15E-01	0.0064	8.22E-01	-0.0132	4.71E-01	0.0290
1209	5834-18_3	IL-9	P15248	IL9	9.87E-01	-0.0002	9.27E-01	0.0004	6.56E-01	0.0020	5.01E-01	0.0039	7.20E-02	0.3394	9.58E-01	-0.0038	7.84E-01	-0.0194	8.86E-01	0.0070
1210	5837-49_3	LIF sR	P42702	LIFR	2.14E-01	-0.0112	2.42E-01	-0.0044	9.85E-01	-0.0001	2.73E-01	0.0056	6.19E-01	0.0836	7.24E-01	0.0228	7.15E-01	0.0229	1.73E-01	0.0587
1211	5843-60_3	NG36	Q96KQ7	EHMT2	1.58E-02	-0.0285	3.47E-03	-0.0145	4.24E-02	-0.0109	3.24E-03	-0.0197	1.24E-05	0.9479	7.77E-01	-0.0242	4.58E-03	-0.2329	4.69E-01	-0.0412
1212	5846-24_3	Noggin	Q13253	NOG	7.37E-01	0.0040	5.78E-01	0.0028	5.88E-01	0.0029	6.68E-01	0.0029	7.30E-01	0.0765	2.83E-01	0.0911	8.24E-01	0.0184	7.70E-01	0.0166
1213	5852-6_3	S100A12	P80511	S100A12	8.08E-01	-0.0024	8.00E-01	-0.0010	2.36E-01	0.0052	7.57E-02	0.0098	5.02E-01	0.1218	7.30E-01	-0.0241	3.35E-01	-0.0652	5.76E-01	-0.0260
1214	5854-60_3	tau	P10636	MAPT	6.80E-01	-0.0043	8.07E-01	0.0011	9.85E-01	-0.0001	6.13E-01	-0.0030	7.83E-01	-0.0539	9.59E-01	0.0038	7.90E-01	-0.0194	2.58E-01	0.0564
1215	5858-6_5	14-3-3 prote	P63104	YWHAZ	4.09E-01	-0.0053	5.95E-02	-0.0051	1.65E-03	-0.0090	2.53E-02	-0.0081	3.20E-03	0.3496	6.22E-01	0.0227	2.95E-01	-0.0468	8.72E-01	-0.0049
1216	5861-78_3	3HAO	P46952	HAAO	8.48E-02	-0.0189	2.57E-01	-0.0052	9.77E-01	-0.0001	1.42E-01	-0.0092	4.14E-01	-0.1682	9.21E-02	-0.1328	6.28E-01	0.0373	7.11E-01	0.0195
1217	5864-10_3	aldolase A	P04075	ALDOA	4.44E-01	-0.0066	3.17E-01	-0.0037	4.54E-01	-0.0030	3.72E-01	-0.0044	1.83E-01	-0.2161	6.13E-01	-0.0316	6.17E-01	0.0304	4.66E-01	-0.0303
1218	5867-60_3	ARGI1	P05089	ARG1	2.27E-01	-0.0117	2.81E-01	-0.0044	1.98E-01	0.0057	5.48E-01	0.0033	7.28E-01	-0.0634	6.72E-01	-0.0296	3.86E-01	0.0589	5.18E-01	-0.0301
1219	5870-23_2	BAD	Q92934	BAD	5.62E-02	-0.0109	7.66E-02	-0.0043	8.31E-01	0.0006	3.86E-01	0.0028	9.89E-01	-0.0015	7.63E-01	-0.0124	3.94E-01	0.0342	8.48E-02	-0.0473
1220	5879-51_3	Dynactin sub	Q13561	DCTN2	5.96E-01	0.0058	9.66E-01	-0.0002	2.87E-01	0.0053	2.30E-01	0.0075	8.92E-01	-0.0280	9.03E-01	0.0096	8.96E-01	0.0100	1.33E-01	0.0793
1221	5882-34_2	EF-1-beta	P24534	EEF1B2	9.72E-03	-0.0179	1.81E-02	-0.0069	7.36E-01	-0.0011	9.16E-01	-0.0004	9.06E-01	-0.0155	1.29E-01	-0.0760	8.30E-02	0.0842	6.15E-01	-0.0168
1222	5885-55_3	eIF-4H	Q15056	EIF4H	4.00E-01	0.0043	6.00E-01	0.0011	3.73E-02	-0.0048	3.00E-01	-0.0030	1.91E-01	0.1241	2.76E-01	0.0398	3.27E-01	-0.0347	7.18E-01	-0.0088
1223	5888-29_3	eIF-5A-1	P63241	EIF5A	9.42E-01	-0.0005	6.06E-01	0.0015	2.99E-01	0.0033	8.16E-01	0.0009	5.47E-01	-0.0778	9.86E-01	0.0009	3.36E-01	0.0464	3.58E-01	-0.0304
1224	5897-58_3	Gastrin-rele	P07492	GRP	8.75E-01	-0.0017	7.95E-01	0.0012	2.79E-01	-0.0052	9.20E-01	0.0006	7.57E-01	0.0612	6.62E-01	0.0332	8.98E-01	-0.0095	2.97E-02	0.1093
1225	5900-11_2	HINT1	P49773	HINT1	8.39E-01	-0.0019	9.08E-01	-0.0005	6.92E-01	-0.0017	9.48E-01	0.0003	9.72E-02	-0.2863	5.32E-01	-0.0416	3.67E-01	0.0582	2.00E-01	-0.0567
1226	5903-91_2	HSP70 prote	P11142	HSPA8	3.80E-01	0.0071	8.36E-01	-0.0007	2.47E-01	-0.0043	3.87E-01	-0.0040	9.85E-01	-0.0029	8.79E-02	0.0991	8.03E-01	-0.0141	7.40E-01	-0.0129
1227	5909-51_3	Nucleoside c	P15531	NME1	5.54E-02	0.0184	7.52E-02	0.0072	4.36E-01	0.0034	8.29E-01	0.0012	7.77E-01	0.0511	1.83E-01	0.0922	6.02E-01	0.0352	4.73E-01	0.0332
1228	5915-58_3	PEX5	P50542	PEX5	2.89E-01	-0.0108	2.74E-01	-0.0047	1.91E-01	-0.0060	2.78E-01	-0.0063	8.72E-01	0.0309	2.78E-02	0.1603	9.32E-02	-0.1191	5.48E-01	-0.0293
1229	5918-5_3	PSME1	Q06323	PSME1	5.71E-01	-0.0033	7.30E-01	-0.0009	1.42E-02	0.0065	1.43E-01	0.0049	3.26E-01	-0.1080	2.08E-02	-0.0971	2.36E-02	0.0925	1.59E-01	-0.0397
1230	5921-58_3	S100A7	P31151	S100A7	1.54E-02	0.0237	6.58E-02	0.0076	4.23E-01	-0.0036	6.64E-01	-0.0024	4.41E-01	0.1422	1.50E-01	0.1018	7.86E-01	0.0187	7.62E-01	-0.0144
1231	5927-4_3	WISP-3	O95389	WISP3	1.91E-01	-0.0138	2.33E-02	-0.0100	8.51E-01	0.0009	5.79E-01	-0.0033	4.03E-01	0.1652	3.36E-01	0.0731	5.87E-01	-0.0401	8.71E-02	-0.0864
1232	5934-1_3	Ferritin	P02794 P027	FTH1 FTL	6.45E-01	0.0040	9.14E-01	0.0004	8.64E-03	0.0104	1.47E-02	0.0121	4.44E-01	-0.1256	8.24E-01	0.0141	3.62E-01	0.0558	4.73E-02	-0.0831
1233	5936-53_3	TNF-a	P01375	TNF	3.67E-01	-0.0078	3.49E-01	-0.0034	9.65E-01	-0.0002	6.23E-01	-0.0024	5.34E-01	-0.1009	9.52E-01	-0.0037	9.96E-01	-0.0003	7.43E-01	0.0137
1234	5939-42_3	TWEAK	O43508	TNFSF12	9.02E-01	0.0011	9.10E-01	0.0004	5.37E-01	-0.0025	6.62E-01	-0.0023	6.81E-01	-0.0694	6.58E-01	0.0287	7.26E-01	0.0221	7.36E-01	0.0146
1235	5947-90_3	Tpo	P40225	THPO	7.14E-01	0.0036	4.85E-01	0.0029	5.06E-02	0.0086	2.29E-01	0.0067	5.60E-01	-0.1062	1.34E-02	0.1716	2.48E-01	0.0783	3.50E-01	0.0435
1236	5954-62_3	PTH	P01270	PTH	8.49E-01	0.0021	9.42E-01	0.0003	5.07E-02	-0.0098	2.61E-01	-0.0071	7.13E-02	-0.3742	8.59E-01	0.0142	3.40E-01	0.0741	1.50E-01	0.0765
1237	5957-30_3	Somatostati	P61278	SST	4.84E-01	-0.0070	8.13E-01	0.0010	6.96E-01	-0.0018	6.70E-01	-0.0024	9.25E-01	0.0177	6.89E-02	0.1306	2.75E-01	0.0763	1.73E-01	0.0653
1238	6151-18_3	MP2K3	P46734	MAP2K3	5.60E-01	-0.0043	6.70E-01	0.0013	4.57E-01	0.0025	4.62E-01	0.0031	5.07E-01	0.0911	5.26E-01	-0.0335	9.69E-01	0.0020	9.16E-01	-0.0037
1239	6152-111_3	p53	P04637	TP53	8.38E-01	0.0022	9.05E-01	0.0005	2.16E-01	-0.0060	3.65E-02	-0.0126	1.49E-01	0.2865	4.85E-01	0.0534	8.19E-01	-0.0170	8.43E-01	-0.0101
1240	6641-60_3	PolyUbiquiti	P0CG47	UBB	1.02E-01	-0.0123	5.53E-01	-0.0019	5.95E-01	0.0018	1.88E-02	0.0100	8.52E-02	-0.2423	2.72E-01	-0.0596	1.46E-01	0.0764	7.17E-01	0.0131
1241	6647-55_3	PolyUbiquiti	P0CG48	UBC	9.43E-01	0.0007	4.98E-01	-0.0027	1.06E-02	0.0110	4.05E-01	0.0045	5.09E-01	0.1182	9.99E-01	-0.0001	7.11E-01	0.0248	3.10E-01	-0.0464
1242	6649-51_3	NET1	O95631	NTN1	5.21E-01	0.0068	8.80E-01	0.0007	7.92E-02	0.0085	3.14E-01	0.0061	7.63E-01	0.0602	1.41E-01	0.1126	8.68E-01	-0.0124	8.03E-01	-0.0127
1243	6653-58_3	CD47	Q08722	CD47	1.38E-01	-0.0100	1.94E-01	-0.0037	6.80E-01	0.0013	5.54E-01	0.0023	2.50E-01	0.1446	5.89E-02	-0.0910	2.89E-01	-0.0498	3.91E-01	-0.0277
1244	7624-19_3	ANK2	Q01484	ANK2	8.79E-01	-0.0014	5.90E-01	0.0021	1.53E-02	-0.0099	8.49E-01	-0.0010	9.50E-01	0.0106	5.34E-01	-0.0405	8.53E-01	0.0118	3.80E-02	0.0896
1245	7625-27_3	14-3-3 prote	P27348	YWHAQ	8.02E-01	0.0022	4.69E-01	-0.0027	5.37E-01	-0.0025	7.74E-02	-0.0089	2.12E-01	0.2064	9.45E-01	0.0044	6.86E-02	-0.1122	1.44E-02	-0.1031
1246	7628-40_3	CREL1	Q96HD1	CRELD1	4.29E-01	0.0090	5.25E-01	0.0031	6.23E-01	0.0025	5.06E-01	0.0043	5.98E-01	0.1125	3.68E-01	0.0737	9.88E-01	0.0012	6.36E-01	-0.0259
1247	7638-30_3	Lectin, manr	Q12907	LMAN2	3.41E-02	0.0241	3.38E-02	0.0102	2.05E-01	0.0066	6.58E-01	-0.0029	6.74E-01	-0.0900	7.39E-01	-0.0274	2.69E-03	0.2373	1.05E-02	0.1394
1248	7640-29_3	LRP1B	Q9NZR2	LRP1B	2.37E-01	0.0137	2.18E-01	0.0060	5.20E-01	0.0034	8.59E-01	-0.0012	3.06E-02	-0.4669	5.49E-23	0.7291	1.29E-07	0.4129	6.91E-01	0.0221
1249	7648-9_3	MYPC1	Q00872	MYBPC1	1.66E-02	0.0261	1.02E-02	0.0118	1.43E-02	0.0121	7.32E-01	0.0021	9.82E-01	-0.0047	2.71E-02	0.1730	4.63E-01	0.0562	1.16E-01	0.0823
1250	7655-11_3	N-terminal p	P16860	NPPB	7.82E-03	-0.0263	9.44E-03	-0.0108	5.51E-02	-0.0087	1.78E-03	-0.0176	4.74E-02	0.3686	6.16E-01	0.0360	3.18E-03	-0.2034	7.33E-02	-0.0853
1251	7660-21_3	Tropomyosir	P67936	TPM4	8.14E-01	-0.0019	3.59E-01	-0.0031	2.56E-05	-0.0150	2.79E-03	-0.0135	3.32E-02	0.3180	8.24E-02	0.0998	1.18E-01	-0.0873	7.60E-01	-0.0117
1252	8446-4_3	PACAP-27	P18509	ADCYAP1	9.90E-01	-0.0001	9.90E-01	-0.0001	4.37E-01	0.0039	8.19E-01	0.0014	8.31E-01	0.0439	8.17E-01	0.0182	9.12E-01	-0.0084	8.78E-01	0.0081
1253	8447-11_3	ghrelin	Q9UBU3	GHRL	1.38E-02	-0.0229	1.74E-02	-0.0093	3.60E-01	-0.0039	3.02E-01	-0.0055	3.23E-01	0.1729	6.51E-01	-0.0304	7.29E-01	-0.0227	6.67E-01	-0.0193
1254	8450-36_3	PACAP-38	P18509	ADCYAP1	1.36E-01	0.0149	2.83E-01	0.0045	7.78E-02	0.0080	7.51E-01	0.0018	7.07E-01	0.0707	7.75E-02	0.1270	4.19E-01	-0.0566	1.83E-02	-0.1128
1255	8458-111_3	a-Synuclein	P37840	SNCA	9.21E-03	-0.0206	5.81E-02	-0.0064	1.38E-01	0.0054	2.82E-01	0.0049	3.84E-02	-0.3082	5.74E-01	0.0323	2.08E-02	0.1282	1.58E-02	-0.0919
1256	8459-10_3	BMP-6	P22004	BMP6	1.35E-01	-0.0153	1.40E-01	-0.0064	4.98E-01	0.0032	8.22E-01	-0.0013	3.63E-01	0.1750	1.37E-01	-0.1095	5.00E-01	0.0484	7.31E-01	-0.0170
1257	8462-18_3	HGH	P01241	GH1	6.00E-01	0.0056	8.06E-01	0.0011	7.21E-01	0.0017	7.07E-01	-0.0023	8.91E-01	0.0275	3.13E-01	0.0776	2.26E-01	0.0904	2.60E-01	0.0577
1258	8463-2_3	SOD3	P08294	SOD3	4.99E-01	-0.0067	6.94E-01	-0.0016	8.90E-01	-0.0006	4.14E-01	-0.0046	1.16E-01	0.2910	6.98E-01	0.0277	5.83E-01	-0.0380	9.48E-01	-0.0031
1259	8464-31_3	RSPO4	Q2I0M5	RSPO4	6.66E-01	0.0043	3.05E-01	0.0043	9.41E-01	0.0003	9.21E-01	-0.0006	5.28E-01	-0.1171	3.55E-01	0.0660	9.77E-01	0.0020	4.59E-01	0.0352
1260	8465-52_3	Cathepsin H	P09668	CTSH	9.78E-01	0.0002	7.14E-01	0.0011	5.07E-01	-0.0022	6.45E-01	-0.0019	7.46E-01	-0.0440	4.46E-01	0.0398	5.71E-01	-0.0287	7.11E-01	0.0129
1261	8467-9_3	Activin AB	P08476 P09	INHBA INHB	6.64E-01	-0.0036	6.99E-01	-0.0014	2.18E-01	-0.0047	2.03E-01	-0.0061	8.86E-01	-0.0225	3.22E-01	0.0596	3.03E-01	-0.0602	6.08E-01	-0.0206
1262	8468-19_3	PSA	P07288	KLK3	8.30E-02	-0.0115	4.60E-02	-0.0056	5.79E-01	0.0017	4.74E-01	0.0027	5.42E-01	0.0759	4.76E-01	-0.0340	6.91E-01	0.0184	1.13E-01	0.0503
1263	8469-41_3	IGFBP-2	P18065	IGFBP2	5.13E-07	-0.0571	6.72E-09	-0.0275	3.20E-02	-0.0114	2.31E-01	-0.0080	2.98E-03	0.6453	5.08E-01	-0.0559	1.19E-02	-0.2047	2.66E-01	-0.0625
1264	8470-213_3	RNase H1	O60930	RNASEH1	6.73E-01	-0.0041	9.89E-01	-0.0001	6.81E-01	-0.0018	1.41E-01	-0.0081	3.60E-01	-0.1664	3.91E-01	0.0599	1.52E-01	-0.0971	8.50E-01	-0.0088
1265	8474-6_3	ROR1	Q01973	ROR1	7.62E-04	-0.0338	2.12E-03	-0.0130	3.32E-01	-0.0045	9.10E-01	0.0007	2.65E-01	0.2125	2.95E-01	-0.0768	2.67E-01	-0.0790	3.87E-01	-0.0422
1266	8476-11_3	CgA	P10645	CHGA	9.80E-04	-0.0366	5.14E-03	-0.0132	8.13E-01	-0.0012	2.00E-01	0.0082	7.37E-04	0.7017	8.59E-02	-0.1387	1.80E-01	-0.1053	6.35E-02	0.0998
1267	8479-4_3	MMP-10	P09238	MMP10	4.77E-02	-0.0161	9.24E-02	-0.0058	5.47E-02	-0.0071	7.27E-03	-0.0124	3.91E-01	-0.1313	7.71E-01	0.0171	4.84E-01	0.0400	3.95E-01	-0.0333
1268	8480-29_3	FBLN3	Q12805	EFEMP1	1.71E-02	0.0257	1.89E-01	0.0060	1.69E-01	-0.0068	2.32E-01	-0.0074	2.54E-01	0.2308	4.93E-01	-0.0535	1.63E-01	-0.1054	5.54E-01	0.0307
1269	8484-24_3	Leptin	P41159	LEP	6.21E-27	0.0742	1.55E-24	0.0301	4.15E-01	0.0030	4.21E-01	0.0037	3.02E-02	-0.3215	3.56E-01	0.0529	9.24E-02	0.0934	5.40E-02	0.0733
1270	8485-7_3	KEAP1	Q14145	KEAP1	7.14E-01	-0.0039	9.35E-01	0.0004	9.72E-01	-0.0002	7.02E-01	0.0023	1.34E-01	-0.2947	6.54E-01	-0.0339	2.01E-02	0.1697	6.03E-01	-0.0262
1271	8488-33_3	gpIIbIIIa	P08514 P05	ITGA2B ITGE	9.84E-02	-0.0155	1.99E-01	-0.0051	2.66E-01	0.0048	4.44E-01	0.0041	1.54E-01	0.2509	8.07E-01	0.0166	9.42E-01	-0.0048	6.54E-01	-0.0203
1272	9168-31_3	Eotaxin-3	Q9Y258	CCL26	7.63E-01	-0.0027	4.36E-01	-0.0030	3.60E-01	-0.0038	3.11E-01	-0.0052	3.55E-02	0.3551	5.67E-01	0.0374	2.39E-01	-0.0745	8.32E-01	-0.0092
1273	9169-14_3	SUMO3	P55854	SUMO3	1.66E-01	0.0068	1.35E-01	0.0031	3.92E-01	0.0019	8.49E-01	0.0005	2.34E-01	-0.1098	5.58E-01	-0.0208	5.09E-01	0.0227	3.88E-02	-0.0486
1274	9170-24_3	IL-17	Q16552	IL17A	5.18E-01	-0.0069	7.36E-01	0.0015	4.94E-01	0.0033	7.58E-01	-0.0019	9.22E-01	0.0195	3.20E-01	0.0759	1.23E-01	0.1141	5.87E-01	0.0276
1275	9171-11_3	CSRP3	P50461	CSRP3	2.37E-02	0.0260	6.14E-02	0.0091	1.19E-01	0.0082	6.88E-01	0.0027	9.55E-01	-0.0123	1.36E-01	0.1235	2.57E-01	0.0914	7.63E-02	0.0978
1276	9172-69_3	MMP-8	P22894	MMP8	3.50E-01	0.0099	1.13E-01	0.0070	4.82E-01	-0.0034	7.67E-01	-0.0018	3.45E-01	-0.1868	2.72E-01	0.0835	6.86E-01	-0.0299	5.75E-01	-0.0285
1277	9173-21_3	PGM1	P36871	PGM1	1.39E-02	0.0265	1.74E-01	0.0062	4.73E-01	0.0035	6.51E-01	-0.0028	2.95E-01	0.2125	4.69E-01	0.0565	8.54E-01	0.0139	9.43E-01	0.0037
1278	9175-48_3	DSCAM	O60469	DSCAM	9.12E-01	-0.0010	9.43E-01	0.0003	2.73E-01	-0.0046	4.58E-01	-0.0039	6.22E-01	0.0856	7.32E-01	-0.0229	3.58E-01	-0.0597	8.45E-01	0.0087
1279	9176-3_3	MUC1	P15941	MUC1	1.20E-01	-0.0161	1.71E-01	-0.0060	8.08E-01	0.0011	2.96E-01	-0.0062	6.10E-01	0.0992	9.34E-01	0.0061	8.61E-01	0.0127	2.42E-01	0.0581
1280	9177-6_3	FAM3B	P58499	FAM3B	2.64E-01	-0.0129	4.42E-01	-0.0037	5.98E-01	-0.0028	6.83E-01	0.0027	7.79E-01	0.0606	3.59E-01	0.0760	3.02E-02	0.1734	9.43E-01	0.0039
1281	9178-30_3	NEUREGULII	Q02297	NRG1	5.92E-01	0.0051	3.50E-01	0.0038	8.87E-02	0.0074	8.81E-01	-0.0008	9.20E-01	-0.0179	9.34E-01	0.0057	2.57E-01	0.0755	2.23E-01	-0.0556
1282	9180-6_3	INGR2	P38484	IFNGR2	8.61E-01	-0.0019	4.90E-01	0.0032	6.56E-01	0.0022	8.36E-01	0.0013	6.32E-01	0.0973	2.08E-01	0.0982	1.31E-01	0.1142	1.04E-02	0.1323
1283	9182-3_3	sLRP1	Q07954	LRP1	1.54E-01	-0.0114	2.30E-01	-0.0040	8.29E-02	-0.0063	7.41E-01	-0.0015	2.60E-01	0.1685	5.70E-01	0.0327	5.77E-01	0.0311	5.04E-01	0.0256
1284	9183-7_3	IFN-a/b R1	P17181	IFNAR1	2.05E-01	-0.0078	1.21E-01	-0.0040	3.52E-01	-0.0026	9.09E-01	-0.0004	6.19E-01	-0.0577	6.56E-01	0.0199	5.99E-02	0.0811	6.85E-01	-0.0121
1285	9185-15_3	TFF1	P04155	TFF1	4.69E-03	-0.0317	1.57E-02	-0.0115	4.77E-01	-0.0037	4.10E-01	-0.0053	3.82E-02	0.4377	7.46E-01	0.0264	2.86E-02	-0.1723	7.17E-02	-0.0975
1286	9187-2_3	HMGN1	P05114	HMGN1	3.36E-02	0.0195	2.34E-02	0.0087	2.63E-01	-0.0047	5.53E-01	-0.0031	8.41E-01	-0.0346	7.82E-02	0.1163	2.20E-01	0.0788	5.15E-01	-0.0288
1287	9188-119_3	MIG	Q07325	CXCL9	6.90E-03	-0.0267	7.19E-02	-0.0075	9.11E-01	0.0005	1.53E-01	0.0081	8.30E-01	-0.0402	4.82E-01	-0.0505	3.88E-02	-0.1432	1.07E-01	-0.0769
1288	9190-7_3	CD63	P08962	CD63	3.74E-01	0.0080	5.41E-01	0.0023	8.27E-02	0.0070	5.53E-01	-0.0030	9.15E-01	0.0180	8.57E-01	0.0117	5.76E-02	0.1185	7.49E-01	0.0137
1289	9191-8_3	Trefoil facto	Q03403	TFF2	1.59E-03	-0.0255	3.00E-02	-0.0074	6.06E-01	-0.0019	6.06E-01	0.0024	3.07E-01	0.1562	7.06E-01	-0.0221	3.05E-01	0.0584	9.44E-01	0.0028
1290	9196-8_3	Galectin-7	P47929	LGALS7	2.57E-01	0.0117	1.70E-01	0.0060	6.80E-01	0.0019	8.56E-01	-0.0011	8.62E-01	0.0336	1.34E-01	0.1110	3.17E-01	0.0722	1.12E-01	0.0784
1291	9197-4_3	LEG9	O00182	LGALS9	1.96E-01	0.0136	2.12E-01	0.0055	7.20E-01	-0.0017	9.75E-01	-0.0002	6.30E-02	-0.3655	7.51E-01	0.0241	5.82E-01	0.0405	7.99E-01	-0.0129
1292	9199-6_3	UB2G2	P60604	UBE2G2	3.15E-03	0.0336	2.62E-03	0.0144	3.92E-02	0.0107	1.75E-03	0.0203	2.61E-03	-0.6414	4.75E-01	0.0591	9.60E-05	0.3077	1.77E-03	0.1704
1293	9201-13_3	Transgelin-2	P37802	TAGLN2	7.16E-01	0.0036	6.08E-01	0.0021	6.09E-01	-0.0023	3.41E-01	-0.0053	5.10E-02	0.3542	5.85E-01	0.0383	7.57E-01	-0.0210	3.70E-02	0.0969
1294	9202-309_3	ATPO	P48047	ATP5O	4.07E-01	-0.0081	9.61E-02	-0.0068	3.45E-01	-0.0042	1.96E-01	-0.0072	5.35E-01	0.1137	2.96E-02	0.1522	8.06E-01	0.0168	8.22E-01	0.0105
1295	9204-33_3	Corticotropir	P01189	POMC	7.26E-01	-0.0034	6.19E-01	0.0020	4.12E-01	0.0036	5.30E-02	0.0105	5.20E-02	-0.3474	2.53E-01	-0.0788	1.15E-01	0.1054	9.30E-01	-0.0040
1296	9207-60_3	QORL1	O95825	CRYZL1	3.67E-01	0.0105	3.19E-01	0.0049	3.05E-01	0.0054	3.99E-01	0.0056	3.90E-02	-0.4479	4.19E-06	0.3753	3.93E-19	0.6577	2.85E-01	0.0597
1297	9211-19_3	PEDF	P36955	SERPINF1	5.15E-02	0.0218	1.49E-02	0.0114	5.90E-04	0.0173	3.76E-04	0.0224	1.90E-01	-0.2750	9.01E-03	-0.2092	4.15E-02	0.1592	1.33E-01	0.0808
1298	9212-22_3	CATF	Q9UBX1	CTSF	7.47E-02	-0.0203	6.52E-02	-0.0088	8.31E-01	-0.0011	9.32E-01	0.0006	5.54E-03	0.5881	7.64E-02	0.1452	1.53E-01	0.1138	2.33E-01	0.0652
1299	9213-24_3	FTCD	O95954	FTCD	7.44E-02	0.0205	7.42E-02	0.0087	7.66E-01	-0.0016	9.49E-01	0.0004	1.72E-01	-0.2943	3.85E-02	0.1710	5.86E-03	0.2201	9.13E-01	-0.0061
1300	9215-117_3	UBP25	Q9UHP3	USP25	9.16E-01	0.0012	8.99E-01	-0.0006	4.70E-01	0.0036	2.09E-01	-0.0079	2.56E-01	0.2348	8.96E-01	-0.0104	5.42E-01	0.0471	7.80E-01	0.0148
1301	9216-100_3	PLXB2	O15031	PLXNB2	9.45E-02	0.0171	7.78E-02	0.0076	2.74E-01	-0.0051	8.44E-01	0.0012	8.06E-03	0.5043	9.89E-01	-0.0010	4.18E-02	-0.1452	2.34E-05	0.2033
